# Liensinine reshapes the immune microenvironment and enhances immunotherapy by reprogramming metabolism through the AMPK-HIF-1α axis in hepatocellular carcinoma

**DOI:** 10.1186/s13046-025-03477-6

**Published:** 2025-07-15

**Authors:** Jiahao Liu, Xuan Zhang, Xiaofei Fan, Peng Liu, Ze Mi, Hongpei Tan, Pengfei Rong

**Affiliations:** 1 Department of Radiology, Third Xiangya Hospital, Central South University, Changsha, 410000 China; 2 Shandong Medical College, Jinan, Shandong 250002 China; 3 The Second Affiliated Hospital, School of Medicine, The Chinese University of Hong Kong, Shenzhen (CUHK- Shenzhen), Longgang District People’s Hospital of Shenzhen, Guangdong, 518172 China

**Keywords:** Liensinine, Metabolic reprogramming, Immunotherapy, HCC, AMPK, Hif-1α, Angiogenesis, Macrophage polarization

## Abstract

**Background:**

Hepatocellular carcinoma (HCC) is a leading cause of cancer-related mortality, with limited treatment options in advanced stages. Liensinine, a natural alkaloid derived from *Nelumbo nucifera*, has shown promise as an anticancer agent. However, its underlying mechanisms, particularly in modulating tumor metabolism and immune responses, remain poorly understood. This study aimed to investigate the antitumor effects of Liensinine in HCC, focusing on its ability to modulate metabolic pathways, immune responses, and the tumor microenvironment.

**Methods:**

HCC cell lines (HUH7 and Hep1-6) were treated with Liensinine in vitro to assess cell viability, migration, proliferation, and apoptosis. Metabolic reprogramming was analyzed through RNA sequencing, Seahorse metabolic assays, and glucose/lactate measurements. The effects on immune cells were studied by treating THP-1 macrophages and peripheral blood mononuclear cells (PBMCs) with conditioned media from Liensinine-treated cells. In vivo, subcutaneous xenograft and orthotopic liver cancer models were used to evaluate the therapeutic efficacy of Liensinine combination with radiotherapy and immunotherapy.

**Results:**

Liensinine inhibited HCC cell viability, migration, and proliferation, promoting apoptosis and shifting metabolism from glycolysis to oxidative phosphorylation. This metabolic reprogramming was linked to the activation of the AMPK-HIF-1α axis and increased ROS production. Furthermore, Liensinine induced Endoplasmic reticulum (ER) stress, as evidenced by elevated levels of CHOP and ATF4, which contributed to AMPK activation and suppression of HIF-1α. Liensinine reduced PD-L1 expression, enhanced M1 macrophage polarization, and promoted CD8 + T cell infiltration into tumors. In vivo, Liensinine significantly suppressed tumor growth, reduced vascular density, and reshaped the immune microenvironment by promoting M1 macrophage polarization. Combination therapy with Liensinine, radiotherapy, and immunotherapy resulted in synergistic effects, including enhanced tumor cell apoptosis, increased immune cell infiltration, and improved therapeutic efficacy.

**Conclusion:**

Liensinine exerts potent antitumor effects in HCC by reprogramming tumor metabolism, inducing ER stress, enhancing immune responses, and modulating the TME. The combination of Liensinine with immunotherapy and radiotherapy significantly improves therapeutic efficacy, suggesting its potential as a novel treatment strategy for HCC.

**Graphical abstract:**

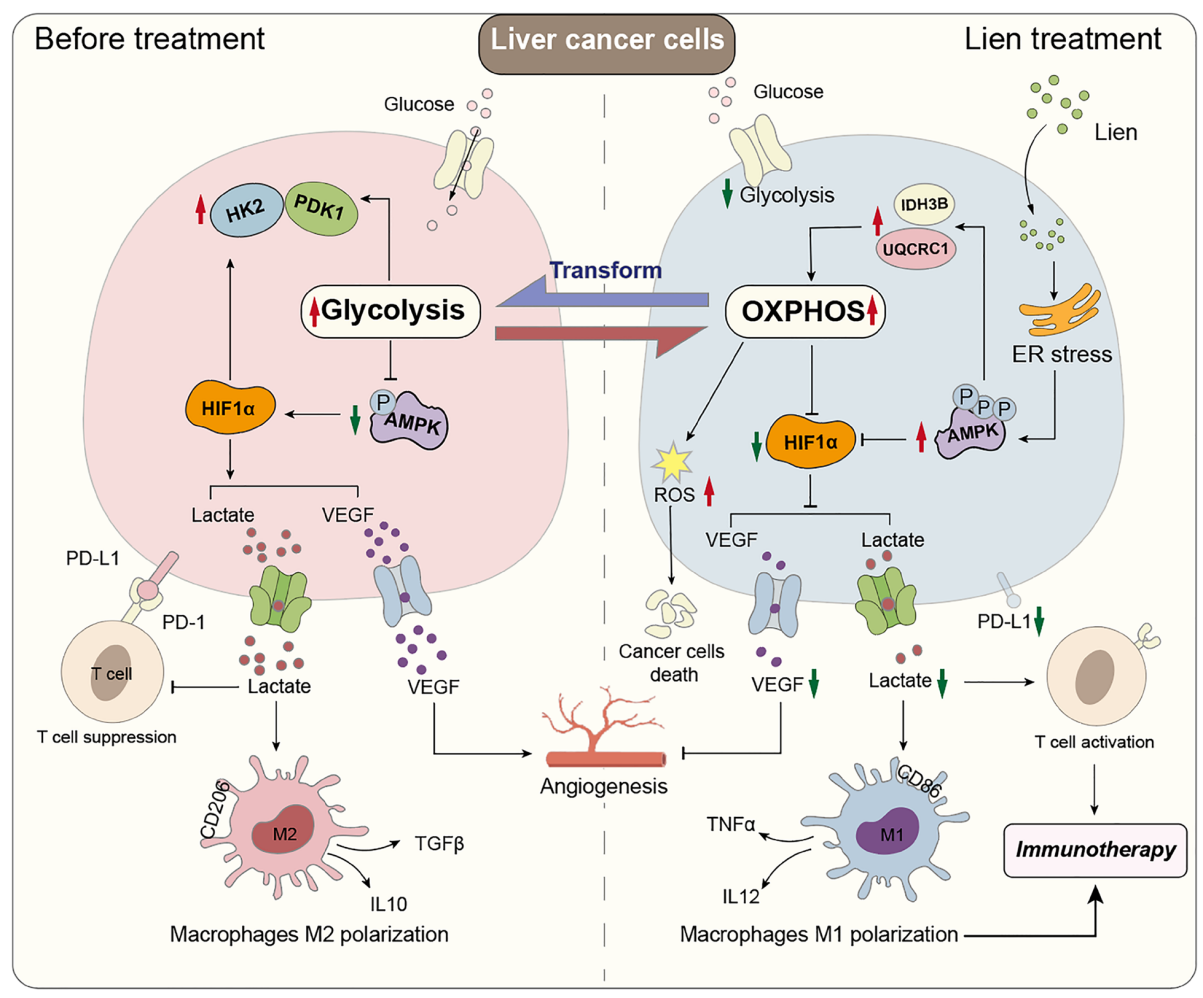

**Supplementary Information:**

The online version contains supplementary material available at 10.1186/s13046-025-03477-6.

## Introduction

Hepatocellular carcinoma (HCC) represents one of the most prevalent and deadly malignancies worldwide, contributing significantly to cancer-related mortality [1]. Despite advancements in treatment, such as surgical resection, liver transplantation, and targeted therapies, the prognosis for patients with advanced HCC remains poor [2, 3]. The high incidence of recurrence and the resistance to conventional therapeutic approaches necessitate the development of novel strategies targeting the underlying molecular mechanisms that drive HCC progression [4].

A hallmark of cancer, including HCC, is metabolic reprogramming [5, 6], in which tumor cells shift their energy production from oxidative phosphorylation to aerobic glycolysis, a phenomenon known as the Warburg effect [7]. This metabolic shift supports the rapid proliferation of tumor cells by providing the necessary biosynthetic precursors [8]. This metabolic switch not only fuels tumor growth but also contributes to the establishment of a tumor microenvironment (TME) that promotes immune evasion, angiogenesis, and metastasis [9–12]. As such, targeting key metabolic pathways has emerged as a promising approach in cancer therapy [13].

In recent years, natural compounds with anticancer properties have garnered increasing attention as potential therapeutic agents [14–16]. Liensinine, a bisbenzylisoquinoline alkaloid extracted from the seed embryo of Nelumbo nucifera (lotus), has demonstrated antitumor properties in several cancers [17, 18]. Recent studies suggest that Liensinine can inhibit the Kv10.1 channel and can reduce the expression of HIF-1α protein [18–20]. Which suggests the potential of Liensinine in tumor treatment, but its specific regulatory mechanism and the impact on tumor metabolism and immune microenvironment are still poorly understood. This study aims to explore the therapeutic potential of Liensinine in HCC, with a focus on its ability to modulate metabolic pathways and immune response within the TME.

## Methods

### Cell culture and reagents

Human umbilical vein endothelial cells (HUVEC), Hepatocellular carcinoma (HCC) cell lines HUH7 and Hep1-6 were purchased from the Shanghai Institutes for Biological Sciences, Chinese Academy of Sciences (Shanghai, China) and cultured in Dulbecco’s Modified Eagle Medium (DMEM) supplemented with 10% fetal bovine serum (FBS), 1% penicillin-streptomycin, and maintained at 37 °C in a humidified atmosphere with 5% CO2. Liensinine was purchased from [manufacturer name] and dissolved in dimethyl sulfoxide (DMSO) to prepare a stock solution. AZD3965 (MCT1 inhibitor), Dorsomorphin (Compound C), an AMPK inhibitor, was also obtained from [MCE] and dissolved in DMSO. The cultured cell lines were tested to be out of mycoplasma contamination. For details on reagents and antibodies, please refer to the supplementary materials.

### Cell viability assay (CCK8)

To assess the effects of Liensinine on cell viability, a Cell Counting Kit-8 (CCK-8, Dojindo) assay was used. HUH7 and Hep1-6 cells were seeded in 96-well plates at a density of 3,000 cells per well and allowed to adhere overnight. Cells were treated with different concentrations of Liensinine (0, 20, 40, 60, 80 µM) for 24 h. After treatment, 10 µL of CCK-8 reagent was added to each well, and the plates were incubated at 37 °C for 2 h. Absorbance was measured at 450 nm using a microplate reader (Bio-Rad). Experiments were performed in triplicate, and results were expressed as the percentage of cell viability relative to untreated controls.

### Colony formation assay

To evaluate the long-term effects of Liensinine on cell proliferation, a colony formation assay was performed. HUH7 and Hep1-6 cells were seeded at a low density (500 cells per well) in 6-well plates and treated with 30 µM Liensinine. After 10–14 days of culture, cells were fixed with 4% paraformaldehyde for 20 min and stained with 0.5% crystal violet. Colonies containing more than 50 cells were counted manually under a microscope. The experiment was performed in triplicate.

### Apoptosis assay

Apoptosis was measured using the Annexin V-FITC/Propidium Iodide (PI) apoptosis detection kit (BD Biosciences). HUH7 and Hep1-6 cells were treated with Liensinine (30 µM) for 24 h. Cells were then harvested, washed with cold PBS, and stained with Annexin V-FITC and PI according to the manufacturer’s protocol. Stained cells were analyzed using flow cytometry (BD FACSCalibur), and the percentage of apoptotic cells (Annexin V positive) was quantified. Data were analyzed using FlowJo software (TreeStar).

### Transwell migration and invasion assay

To assess the effects of Liensinine on the migration and invasion abilities of HCC cells, Transwell assays were performed. For the migration assay, Huh7 and Hep1-6 cells (5 × 10^4 cells/well) were suspended in serum-free medium and seeded into the upper chamber of 24-well Transwell inserts (8 μm pore size). The lower chamber was filled with DMEM containing 10% FBS as a chemoattractant. After 24 h of incubation at 37 °C, non-migrated cells on the upper surface of the membrane were removed with a cotton swab. Migrated cells on the lower surface were fixed with 4% paraformaldehyde for 15 min, stained with 0.1% crystal violet, and counted under a microscope.

### EdU cell proliferation assay

Cell proliferation was assessed using the EdU (5-ethynyl-2’-deoxyuridine) assay kit (RiboBio) according to the manufacturer’s instructions. HUH7 and Hep1-6 cells were seeded in 96-well plates at a density of 5,000 cells per well and treated with Liensinine (40 µM) for 24 h. Cells were then incubated with 50 µM EdU for 2 h at 37 °C. After fixation with 4% paraformaldehyde, cells were permeabilized with 0.3% Triton X-100, followed by reaction with Apollo^®^ staining solution. Nuclei were counterstained with DAPI (Sigma). The percentage of EdU-positive cells was determined by fluorescence microscopy. The experiment was conducted in triplicate.

### RNA sequencing (RNA-seq)

Total RNA was extracted from Liensinine-treated and untreated HUH7 cells using TRIzol reagent (Invitrogen) following the manufacturer’s instructions. RNA quality and integrity were assessed using the Agilent 2100 Bioanalyzer. High-quality RNA samples were used to prepare RNA-seq libraries using the Illumina TruSeq RNA Library Prep Kit. Sequencing was performed on the Illumina HiSeq platform. Differentially expressed genes (DEGs) were identified using DESeq2 software, with a fold change ≥ 1 and p-value ≤ 0.05 considered significant. Gene Set Enrichment Analysis (GSEA) was performed to identify pathways significantly enriched in Liensinine-treated cells.

### Western blot, immunohistochemistry (IHC), quantitative real-time PCR (qRT-PCR) and Immunofluorescence (IF)

The detailed protocols for Western Blot, Immunohistochemistry (IHC), Quantitative Real-Time PCR (qRT-PCR) and Immunofluorescence (IF) are described in the Supplementary information.

### Tube formation assay

To assess the effect of Liensinine on angiogenesis in vitro, a tube formation assay was performed using human umbilical vein endothelial cells (HUVECs) cultured in tumor-conditioned medium (TCM). HUH7 cells were treated with Liensinine (30 µM) or vehicle (Dimethylsulfoxide (DMSO)) for 24 h. Remove the supernatant, wash and add the corresponding culture medium for 24 h, the cell culture supernatants were collected, centrifuged at 3000 rpm for 10 min to remove debris, and used as TCM.

HUVECs were cultured in 6-well plates until they reached 80% confluence and then serum-starved for 6 h. For the tube formation assay, 50 µL of growth factor-reduced Matrigel (BD Biosciences) was added to each well of a 96-well plate and allowed to solidify at 37 °C for 30 min. HUVECs (2 × 10^4^ cells per well) were resuspended in the TCM and seeded onto the Matrigel-coated wells. Cells were incubated at 37 °C for 6–8 h, during which time tube-like structures formed. Tube formation was visualized using an inverted microscope, and images were captured at 5 random fields per well.

### Seahorse metabolic assay

The Seahorse XF Glycolysis Stress Test and Mito Stress Test (Agilent Technologies) were used to measure the extracellular acidification rate (ECAR) and oxygen consumption rate (OCR), respectively. HUH7 cells were treated with 40 µM Liensinine for 24 h before being seeded into XF96 microplates. After equilibration, ECAR and OCR were measured using the Seahorse XF96 analyzer following the manufacturer’s protocols. Results were normalized to cell number.

### In vivo tumor xenograft model

Male BALB/c nude mice (6–8 weeks old) were used for in vivo experiments. Huh7 cells (5 × 10^6^) were subcutaneously injected into the flanks of mice. Once tumors reached approximately 100 mm³, mice were randomly divided into control and treatment groups (*n* = 6 per group). Liensinine was administered intraperitoneally at a dose of 20 mg/kg daily, while the control group received vehicle (DMSO). Tumor size was measured with calipers every 3 days, and tumor volume was calculated using the formula: Volume = (length × width²)/2. After 21 days of treatment, tumors were harvested for further analysis.

To evaluate the in vivo antitumor effects of Liensinine in a more clinically relevant model, an orthotopic hepatocellular carcinoma (HCC) mouse model was established. Male C57BL/6 mice (6–8 weeks old) were used for the study. Hep1-6 cells were first transduced with luciferase-expressing lentivirus to enable in vivo bioluminescence imaging. For the orthotopic implantation, Hep1-6-Luc cells (2 × 10^6^ cells in 30 µL of PBS) were injected into the liver lobe of anesthetized mice through a small laparotomy under sterile conditions. Mice were randomly assigned to treatment groups once tumor establishment was confirmed via bioluminescence imaging (typically 14 days post-implantation). Liensinine (20 mg/kg) was administered intraperitoneally daily, while the control group received vehicle. Tumor growth was monitored weekly using in vivo bioluminescence imaging (IVIS Spectrum, PerkinElmer) and small animal MRI (where applicable). For the combination therapy of immunotherapy and radiotherapy, a radiation dose of 4 Gy is given on the 0th and 4th day of radiotherapy, and immunotherapy is administered on the 3rd and 5th day via intraperitoneal administration (anti-PD-L1 100ug), as shown in our previous study [21]. After 16 days of treatment, the mice were sacrificed, and tumors were harvested for histological and immunohistochemical analysis.

All procedures involving animals were conducted in accordance with institutional ethical guidelines and approved by the Animal Care and Use Committee.

### Flow cytometry analysis of tumor-infiltrating immune cells

Tumor tissues were harvested from mice treated with Liensinine or control treatment and were mechanically dissociated into single-cell suspensions using a tumor dissociation kit (Miltenyi) following the manufacturer’s protocol. The cell suspensions were filtered through a 70 μm cell strainer to remove debris. Followed by staining with fluorescently labeled antibodies against CD45 (total leukocytes), CD8 (T cells), CD86 (M1 macrophages), and CD206 (M2 macrophages) (all antibodies from BioLegend). After staining, cells were washed and resuspended in FACS buffer for analysis using a BD FACSCanto II flow cytometer (BD Biosciences). Data were analyzed using FlowJo software. The experiment was repeated in triplicate.

### Statistical analysis

All experiments were performed at least in triplicate. Data are expressed as mean ± standard deviation (SD). Statistical analysis was performed using GraphPad Prism 10.0 (GraphPad Software). Differences between groups were assessed using Student’s t-test or one-way ANOVA followed by Tukey’s post-hoc test, as appropriate. P-values ≤ 0.05 were considered statistically significant.

## Results

### Liensinine inhibits hepatocellular carcinoma cell viability, proliferation, and migration

**Fig. 1:**
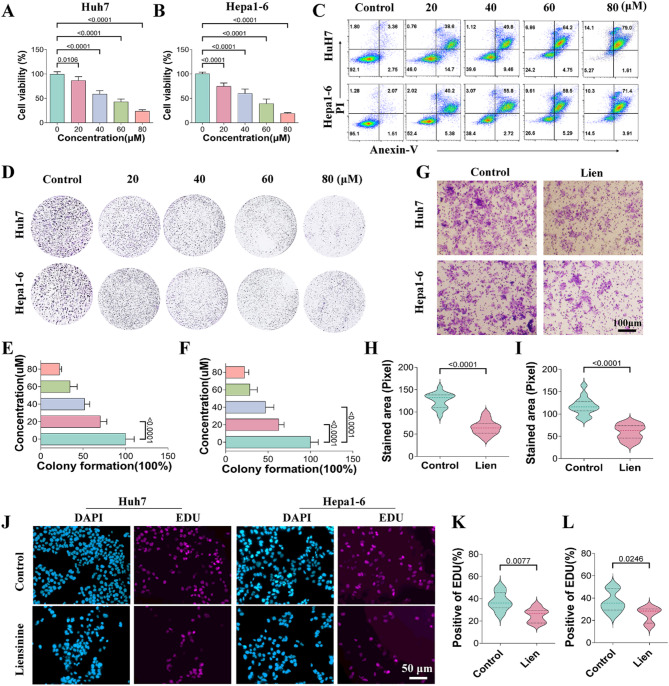
(**A**-**B**) Changes in the viability of Huh7 and Hep1-6 cells under different concentrations of Lien. (**C**) Apoptosis of Huh7 and Hep1-6 cells under different concentrations of Lien, detected by flow cytometry. (**D**-**F**) Clonogenic assay and quantification showing colony formation of Huh7 and Hep1-6 cells under various concentrations of liensinine. (**G**-**I**) Cell migration assay and quantification of Huh7 and Hep1-6 cells treated with 40 µM of liensinine. (**J**-**L**) EDU staining and quantification of proliferative Huh7 and Hep1-6 cells at a concentration of 40 µM liensinine. For the analyses in (**A**, **B**, **E**, **F**, **H**, **I**, **K**, **L**) (*n* = 5 independent samples), Student′s t test was conducted

The effects of Liensinine on HCC cell viability, proliferation, and migration were evaluated in Hep1-6 and HUH7 cell lines. Liensinine treatment resulted in a significant dose-dependent reduction in cell viability, as determined by the Cell Counting Kit-8 (CCK-8) assay (Fig. 1A-B). Further investigation using apoptosis assays and colony formation assays confirmed that Liensinine promoted apoptosis and inhibited the clonogenic potential of HCC cells (Fig. 1C-F). Based on these findings, a concentration of 40 µM Liensinine was selected for subsequent experiments. To assess the impact of Liensinine on cellular migration, Transwell assays were performed. The results demonstrated that Liensinine significantly reduced the migratory ability of Hep1-6 and HUH7 cells (Fig. 1G-I). Additionally, the EdU incorporation assay, which measures cell proliferation, showed a marked reduction in the percentage of proliferating cells following Liensinine treatment (Fig. 1J-L). These findings suggest that Liensinine exerts strong inhibitory effects on HCC cell viability, proliferation, and migration, while promoting apoptosis.

### Liensinine modulates metabolic reprogramming in hepatocellular carcinoma cells

**Fig. 2:**
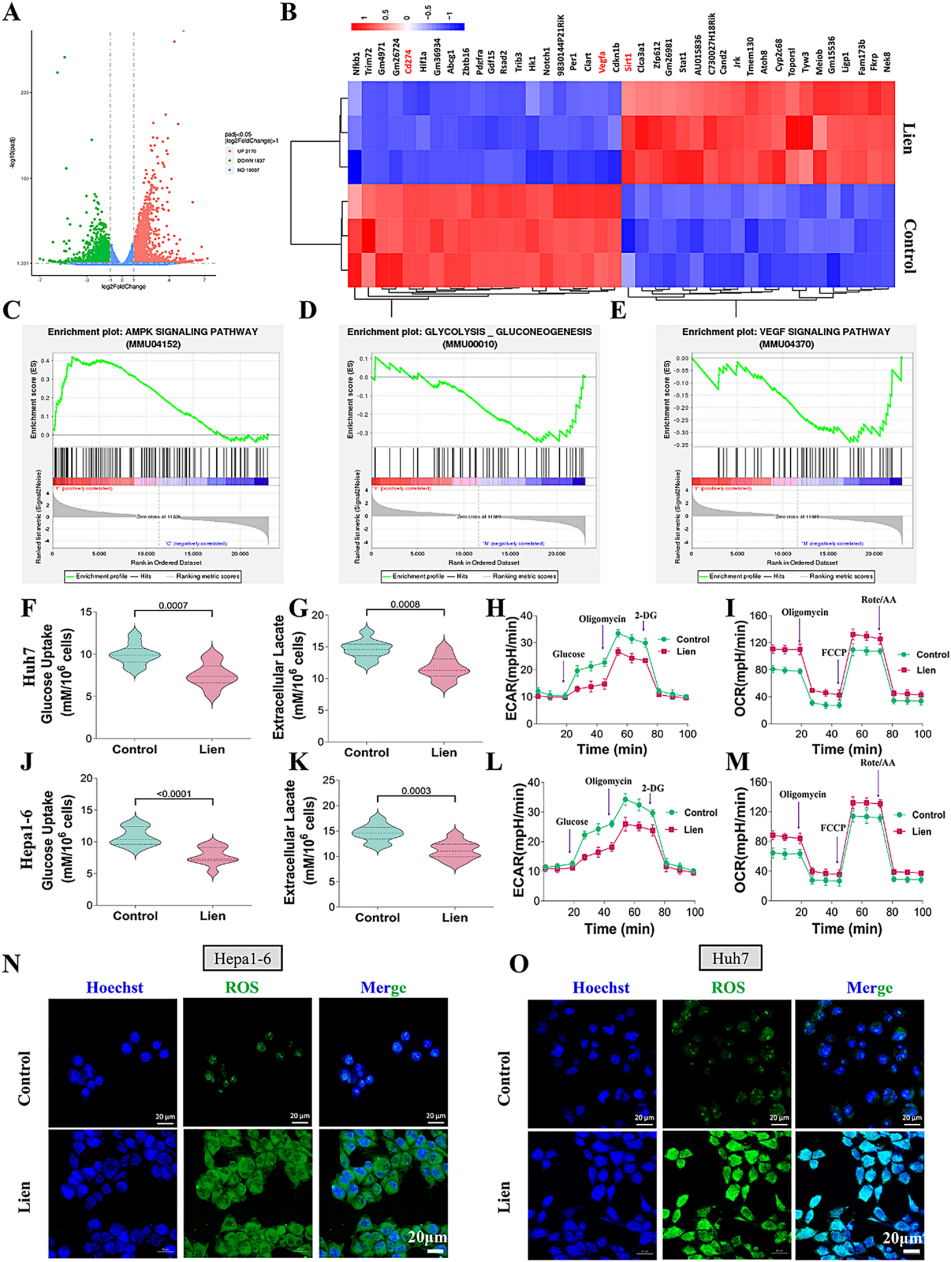
(**A**) Volcano plot showing the differentially expressed genes between liensinine-treated and untreated Huh7 cells. (**B**) Heatmap of differentially expressed genes from the clustering analysis of liensinine-treated and untreated groups. (**C**-**E**) GSEA analysis indicating that liensinine positively regulates the AMPK pathway, while negatively regulating the glycolysis and VEGF pathways. (**F**-**M**) Measurement of glucose uptake, extracellular lactate levels, ECAR, and OCR in HUH7 and Hep1-6 cells with and without liensinine treatment. (**N**-**O**) Fluorescence staining of intracellular ROS levels in HUH7 and Hep1-6 cells treated with or without liensinine. For the analyses in (**F**, **G**, **J**, **K**) (*n* = 10 independent samples), Student′s t test was conducted

To explore the effects of Liensinine on tumor metabolism, RNA sequencing (RNA-seq) was conducted on HUH7 cells treated with Liensinine. Clustering analysis of the top differentially expressed genes revealed 4,007 genes with significant changes (fold change ≥ 1, p-value ≤ 0.05), highlighting the substantial impact of Liensinine on gene expression (Fig. 2A-B). Gene set enrichment analysis (GSEA) revealed that Liensinine significantly upregulated the AMPK pathway, while concurrently downregulating glycolysis-related pathways and the VEGF signaling pathway (Fig. 2C-E). To further assess metabolic reprogramming, glucose uptake and lactate production assays were performed. Seahorse assays confirmed that Liensinine decreased extracellular acidification rates (ECRA) while increasing oxygen consumption rates (OCR) (Fig. 2F-M). Due to the fact that tumor cells typically provide necessary energy for their progression through glycolysis, and the conversion to oxidative phosphorylation promotes high levels of ROS production [22, 23], our results showed that Liensinine increased the level of ROS in liver cancer cells (Fig. 2N-O). These results support the hypothesis that Liensinine inhibits HCC cell growth by inducing metabolic reprogramming towards oxidative phosphorylation.

### Liensinine influences metabolic reprogramming and angiogenesis through AMPK and HIF-1α regulation

**Fig. 3:**
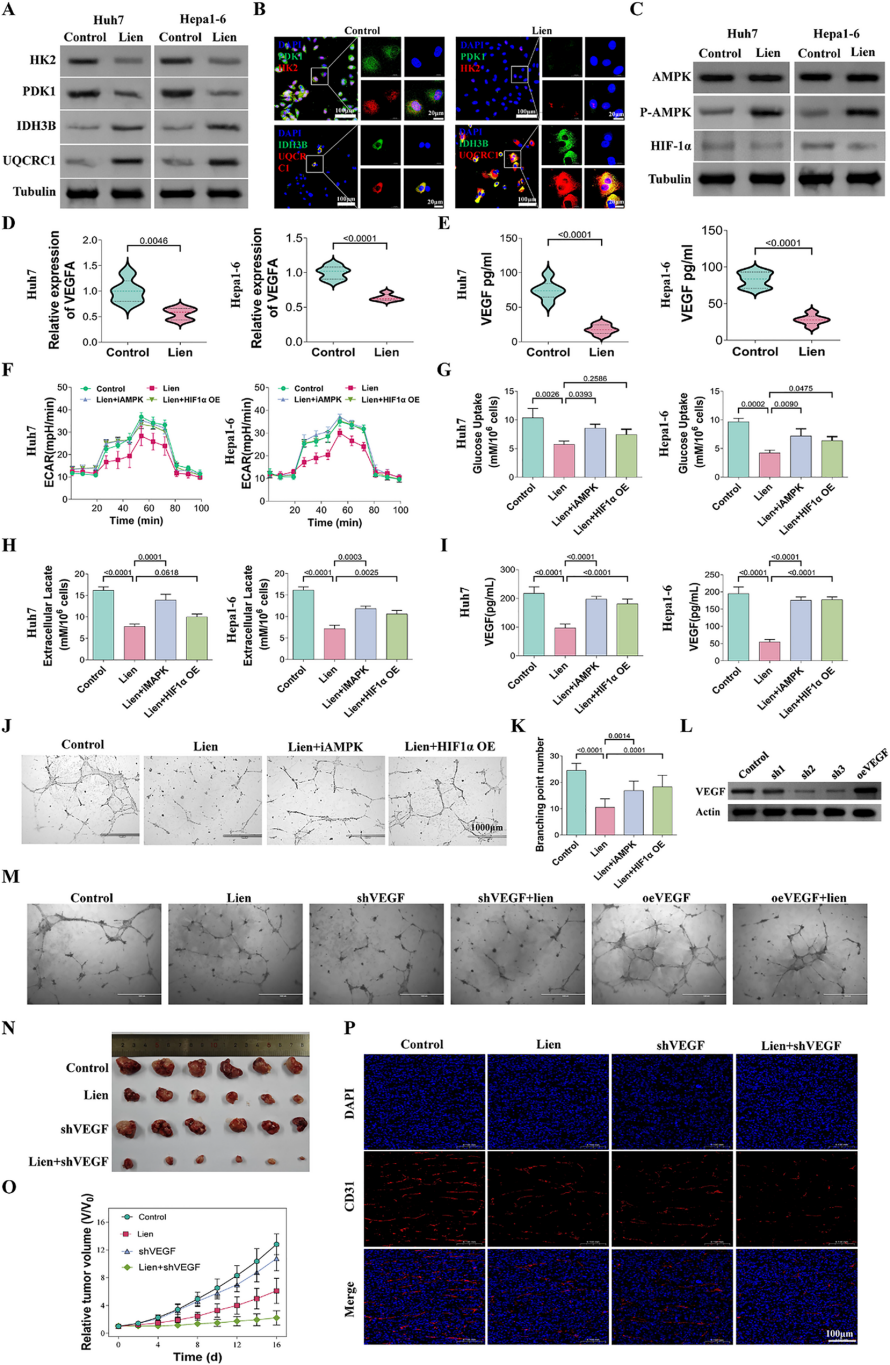
(**A**) Protein expression levels of HK2, PDK1, IDH3B, and UQCRC1 in HUH7 and Hep1-6 cells treated with or without liensinine. (**B**) Fluorescence staining of intracellular HK2, PDK1, IDH3B, and UQCRC1 in Huh7 cells treated with or without liensinine. (**C**) Protein expression levels of AMPK, p-AMPK, and HIF-1α in Huh7 and Hep1-6 cells with or without liensinine treatment. (**D**) RNA expression levels of VEGFA in Huh7 and Hep1-6 cells treated with or without liensinine. (**E**) VEGF levels in the supernatant of Huh7 and Hep1-6 cells treated with or without liensinine, as measured by ELISA. (**F**-**I**) Glucose uptake, extracellular lactate levels, and VEGF levels in the supernatant of Huh7 and Hep 1–6 cells under different treatment conditions. (**J**-**K**) Effect of the supernatant of Huh7 cells on HUVEC cell tube formation under different treatments and corresponding quantitative analysis. (**L**) Stable cell lines (Huh7) with VEGF knockdown and overexpression were constructed using lentivirus, and Western blotting was used to verify the VEGF expression levels. (**M**) Effect of the supernatant of Huh7 cells on HUVEC cell tube formation under different treatments. (**N**-**O**) The effect of Liensinine (Lien) and VEGF knockdown on tumor growth was assessed using a Huh7 xenograft mouse model. Corresponding tumor images and tumor growth curves were generated to evaluate the impact on tumor growth. (**P**) Immunofluorescence staining of CD31 was performed to assess the vascular formation in tumor tissues from different groups. For the analyses in (**D**, **E**, **G**, **H**, **I**, **K**) (*n* = 5 independent samples), Student′s t test was conducted

To investigate the molecular mechanisms underlying Liensinine’s effects on metabolism, the expression of key glycolytic and oxidative phosphorylation-related genes was analyzed. Liensinine treatment decreased the mRNA and protein levels of glycolysis-associated genes (HK2 and PDK1) and increased the expression of oxidative phosphorylation-related genes (IDH3B and UQCRC1) (Fig. 3A and Figure S1). These findings were corroborated by immunofluorescence staining, confirming the impact of Liensinine on cellular metabolism (Fig. 3B). Our RNA sequencing results showed that Liensinine promotes the AMPK pathway, while HIF-1α enhances glycolysis and the expression of VEGF. To understand how Liensinine regulates HIF-1α and AMPK signaling, we analyzed the expression and activation levels of HIF-1α and AMPK in liver cancer cells through Western blotting. Our results showed that Liensinine increased the relative level of AMPK phosphorylation and decreased HIF-1α expression, It also decreased the transcription of VEGFA and lowered the extracellular VEGF levels (Fig. 3C-E). Further experiments, including HIF-1α overexpression and the use of compound C (CC), an AMPK inhibitor, were performed to validate whether Liensinine acts through AMPK and HIF-1α. The results showed that overexpression of HIF-1α and AMPK inhibition partially reversed the metabolic changes and VEGF effects induced by Liensinine (Fig. 3F-I and Figure S2). The tube formation assay in HUEVC cells further confirmed these findings (Fig. 3J-K). To investigate whether Liensinine influences angiogenesis through VEGF, we knocked down and overexpressed VEGF in Huh7 cells (Fig. 3L). The tube formation assay showed no significant difference between the Liensinine-treated group and the VEGF knockdown group, but combining VEGF knockdown with Liensinine exhibited a more pronounced inhibition of tube formation. Overexpression of VEGF partially restored the tube formation inhibition induced by Liensinine (Fig. 3M and Figure S3A). In vivo experiments further demonstrated that both Liensinine and VEGF knockdown inhibited tumor growth, and their combination better suppressed tumor growth and angiogenesis (Fig. 3N-P). This suggests that Liensinine may affect vascular regeneration not only through VEGF. Many studies have mentioned that lactate can influence vascular regeneration [24]. After treating Huh7 cells with a lactate transporter inhibitor, we found that it also affected the tube formation of HUEVCs (Figure S3B). Our previous results indicated that Liensinine can inhibit lactate production, which may be one of the mechanisms by which it affects tumor vasculature.

### Liensinine regulates HIF-1α ubiquitination via AMPK activation through ER stress

**Fig. 4:**
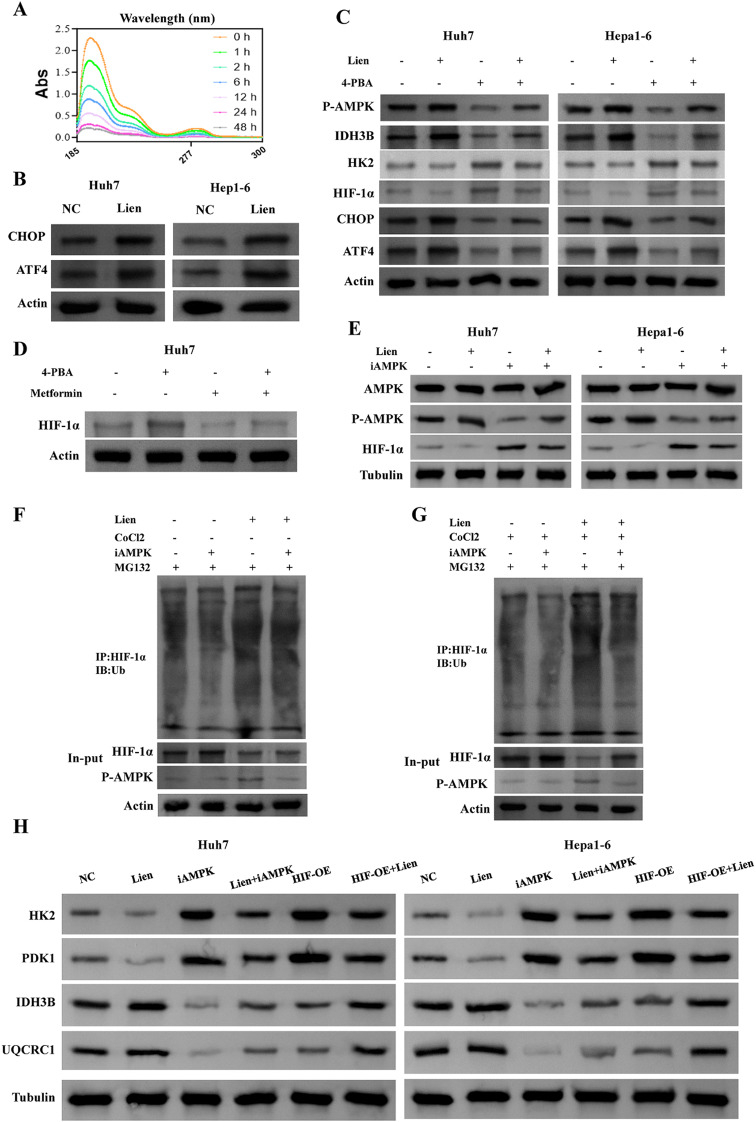
(**A**) The content of Liensinine in the extracellular medium was measured using UV spectroscopy. (**B**) Western blot analysis of endoplasmic reticulum stress-related proteins CHOP, ATF4 under Liensinine treatment. (**C**) Western blot analysis of CHOP, ATF4, P-AMPK, HIF-1a, IDH3B, HK2 under different treatment. (**D**) Western blot analysis of HIF-1a under different treatment. (**E**) Western blot analysis of AMPK, P-AMPK and HIF-1a under different treatment. (**F**-**G**) The effect of different treatments on HIF-1α ubiquitination under normoxic and hypoxic conditions was assessed, with MG132 (20 µM) treatment for 8 h prior to measurement. (**H**) Protein expression levels of HK2, PDK1, IDH3B, and UQCRC1 in Huh7 and Hep1-6 cells under different treatment conditions

The drug typically exerts its effects on cells by activating cell surface receptors or entering cells. Therefore, we evaluated the changes in Liensinine concentration in the extracellular medium. The results showed characteristic absorption peaks of Liensinine at 196 nm and 282 nm. Based on measurements of the medium at different time points, the intensity of these absorption peaks decreased in a time-dependent manner, indicating that the Liensinine concentration in the medium gradually decreased over time. This suggests that Huh7 cells absorbed the Liensinine from the culture medium. Compared to 0 h, the UV absorbance at 24 h and 48 h decreased by 87.1% and 90.9%, respectively, indicating efficient uptake of Liensinine by the Huh7 cells (Fig. 4A). These results confirm that Liensinine is efficiently absorbed by the HUH7 cells, allowing it to exert its intracellular effects. Next, we explored whether the entry of Liensinine into cells affects endoplasmic reticulum (ER) stress, as it has been shown that ER stress can activate AMPK [25]. Western blot analysis revealed that Liensinine treatment significantly increased the protein levels of CHOP and ATF4, two key markers of ER stress We therefore assessed the expression levels of ER stress-related proteins CHOP and ATF4, the results showed that Liensinine increased the protein levels of CHOP and ATF4 (Fig. 4B), Furthermore, the use of the ER stress inhibitor 4-PBA suppressed the Liensinine-induced activation of AMPK and its effects on glycolysis-related proteins, suggesting that ER stress plays a role in Liensinine’s mechanism of action (Fig. 4C). To further elucidate the link between ER stress and AMPK activation in Liensinine’s effects, we investigated the regulation of HIF-1α, a critical regulator of cancer metabolism. We found that the AMPK activator Metformin significantly reduced HIF-1α protein levels, and this effect was not reversed by the ER stress inhibitor 4-PBA (Fig. 4D), suggesting that AMPK activation regulates HIF-1α independently of ER stress. Additionally, the use of the AMPK inhibitor Compound C reversed the Liensinine-induced decrease in HIF-1α levels, further supporting the role of AMPK in regulating HIF-1α (Fig. 4E). We then examined whether Liensinine affects the ubiquitination of HIF-1α, as this post-translational modification is essential for the degradation of HIF-1α under normal and hypoxic conditions. Using CoCl₂ to simulate hypoxia, we found that Liensinine promoted the ubiquitination of HIF-1α in both normoxic and hypoxic conditions. Importantly, the use of the AMPK inhibitor significantly reduced HIF-1α ubiquitination, suggesting that Liensinine’s regulation of HIF-1α involves AMPK-mediated ubiquitination (Fig. 4F-G). Furthermore, overexpression of HIF-1α or inhibition of AMPK partially reversed the changes in glycolytic protein levels induced by Liensinine, reinforcing the involvement of the AMPK-HIF-1α axis in the metabolic effects of Liensinine (Fig. 4H). Therefore, these findings suggest that Liensinine exerts its metabolic regulatory effects by promoting AMPK activation, which leads to the ubiquitination and degradation of HIF-1α through ER stress.

### Liensinine modulates immune cell differentiation and activation in vitro

**Fig. 5:**
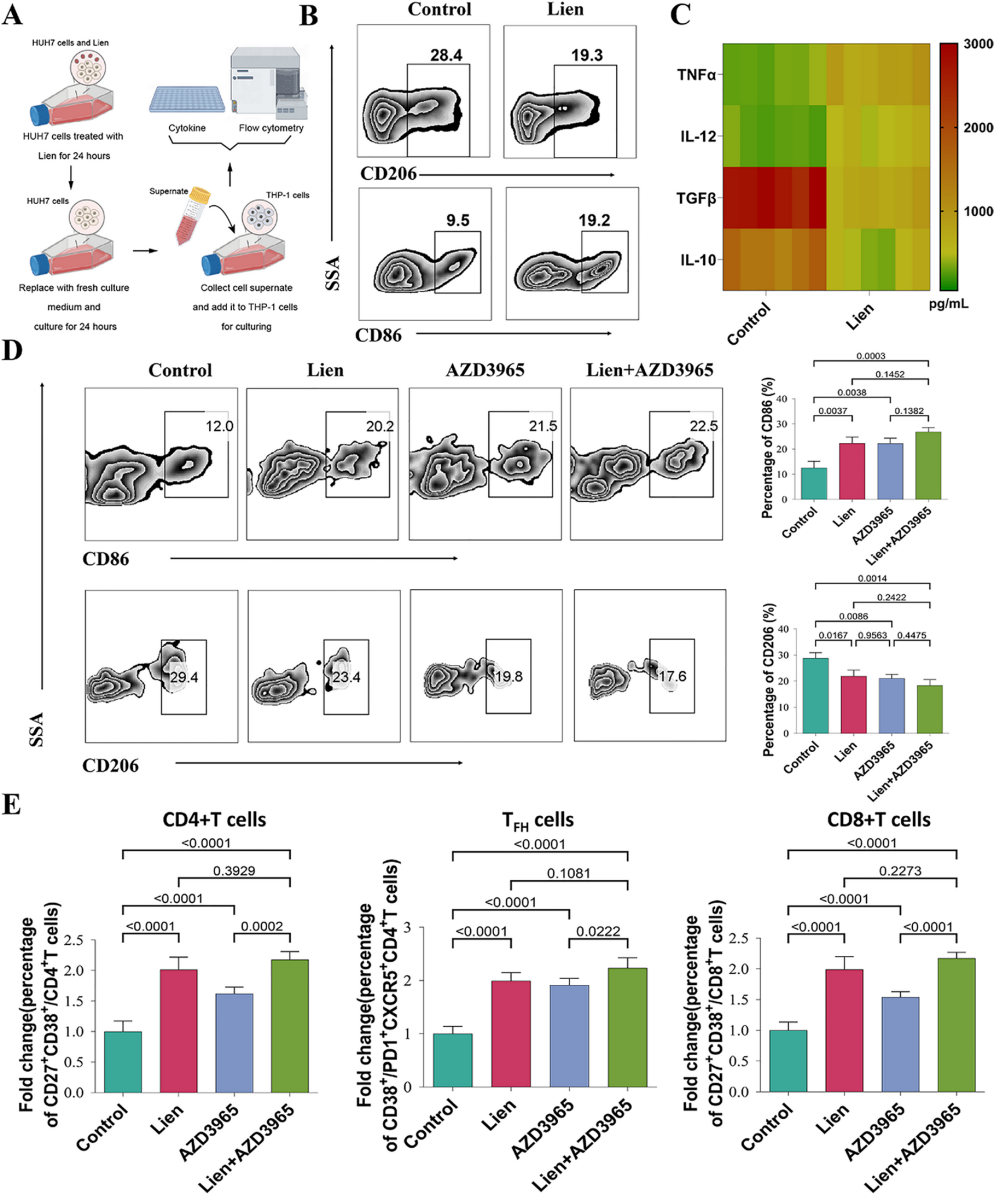
(**A**) Schematic of the macrophage polarization assay. (**B**) Effect of the supernatant from liensinine-treated Huh7 cells on THP-1 cell polarization. (**C**) Levels of IL-10, TGFβ, IL-12, and TNFα in the supernatant. (**D**) Effects of conditioned medium from Huh7 cells under different treatment on THP-1 cell polarization. (**E**) Flow cytometric analysis of the activation of human peripheral immune cells by conditioned medium from Huh7 cells under different treatment conditions. For the analyses in (**D**, **E**) (*n* = 5 independent samples), Student′s t test was conducted

Metabolic reprogramming in tumors often leads to alterations in the tumor microenvironment (TME), which can influence the differentiation and activity of immune cells. To assess the impact of Liensinine on immune cell function, we treated THP-1 macrophages with conditioned medium from Liensinine-treated HUH7 cells. The results showed that the conditioned medium from Liensinine-treated HUH7 cells promoted macrophage polarization toward the M1 phenotype, as evidenced by increased CD86 expression and reduced CD206 expression compared to controls (Fig. 5A-B). Further cytokine analysis revealed a significant reduction in the levels of immunosuppressive cytokines IL-10 and TGF-β, alongside an increase in pro-inflammatory cytokines IL-12 and TNF-α (Fig. 5C). Previous studies have suggested that lactate plays a key role in regulating macrophage polarization [26, 27]. To investigate whether Liensinine’s effects on macrophage polarization are mediated through lactate, we treated THP-1 macrophages with conditioned medium from Liensinine-treated HUH7 cells or from HUH7 cells treated with the lactate transporter inhibitor AZD3965. Both the lactate transporter inhibitor and Liensinine treated conditioned medium promoted M1 macrophage polarization, but there was no significant difference observed between the two treatments, and the combination treatment did not notably increase the proportion of M1 polarization (Fig. 5D). Suggesting that Liensinine influences macrophage polarization through lactate production.

In addition to macrophages, we further studied the effects of Liensinine on T cell activation. Peripheral blood mononuclear cells (PBMCs) from healthy volunteers were exposed to conditioned media from Liensinine-treated Huh7 cells or from Huh7 cells treated with the lactate transporter inhibitor. Flow cytometry analysis showed that both Liensinine and the lactate transporter inhibitor promoted the activation of CD4 + T cells and CD8 + T cells. Notably, Liensinine was more effective in enhancing the functionality of CD4 + and CD8 + T cells compared to the lactate transporter inhibitor. However, there was no significant difference between the two treatments in their effect on follicular helper T cells (Fig. 5E). These results suggest that Liensinine enhances T cell activation, particularly CD4 + and CD8 + T cells, potentially through lactate-mediated metabolic changes in the TME.

### In vivo antitumor effects of liensinine and its synergistic interaction with immunotherapy

**Fig. 6:**
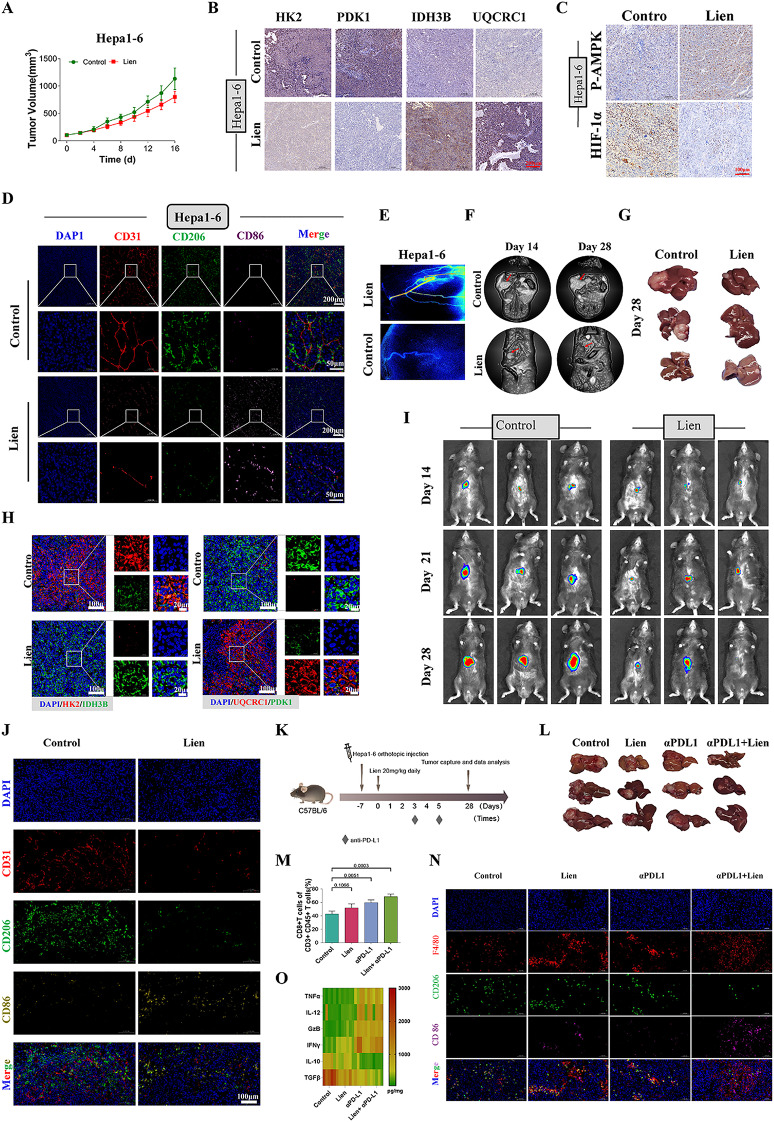
(**A**) Growth curves of Hepa1-6 xenograft tumors. (**B**) Immunohistochemical analysis of HK2, PDK1, IDH3B, and UQCRC1 expression in tumor tissues. (**C**) Immunohistochemical detection of P-AMPK and Hif-1a expression in HuH7 tumor tissues. (**D**) Immunofluorescence staining to assess the expression of CD31 (Red), CD206 (Green), and CD86 (purple) in tumor tissues. (**E**) In vivo angiogenesis of tumors observed through laser speckle imaging. (**F**) T2-weighted MRI images show liver tumors indicated by red arrows. (**G**) Liver tissues were collected and photographed on day 28 post-tumor implantation. (**H**) Immunofluorescence staining shows HK2 (red) and IDH3B (green) expression in liver tumor tissues, UQCRC1 (red) and PDK1 (green) expression were also detected via immunofluorescence. (**I**) Small animal fluorescence imaging was used to monitor liver tumor progression. (**J**) Additionally, CD31 (red), CD206 (green), and CD86 expression were observed in liver tumor tissues through immunofluorescence staining. (**K**) The dosing schedule diagram. (**L**) Liver tissues were collected and photographed on day 28 post-tumor treatment. (**M**) Flow cytometry analysis of the percentage of CD8 + T cells among CD3 + CD45 + T cells across treatment groups. (**N**) F4/80 (red), CD206 (green), and CD86 expression were observed in liver tumor tissues through immunofluorescence staining. (**O**) Cytokine levels in tumor tissue

To assess whether the antitumor effects of Liensinine observed in vitro could be replicated in vivo, we established subcutaneous xenograft models using Huh7 and Hepa1-6 cells. The results demonstrated that Liensinine significantly inhibited tumor growth in both xenograft models (Fig. 6A and Figure S4A). Immunohistochemical analysis of tumor tissues revealed that Liensinine treatment led to a marked reduction in the expression levels of HK2, PDK1, and HIF-1α, while increasing the expression of IDH3B, UQCRC1, and phosphorylated AMPK (P-AMPK) (Fig. 6B-C and Figure S4B-C). Immunofluorescent staining of tumor tissues for the vascular marker CD31 and macrophage markers CD86 (M1) and CD206 (M2) confirmed that Liensinine treatment reduced tumor vascular density, decreased M2 macrophage infiltration, and increased M1 macrophage polarization (Fig. 6D and Figure S4E). To further explore the effects of Liensinine on tumor vasculature, live tumor angiography was performed, which revealed a marked reduction in tumor vascular density in the Liensinine-treated group compared to controls (Fig. 6E and Figure S4D).

Additionally, we established an orthotopic liver cancer transplantation tumor model to further evaluate Liensinine’s antitumor effects in a more clinically relevant setting. Small animal MRI and live fluorescence imaging showed that Liensinine significantly suppressed tumor progression in the orthotopic liver cancer transplantation model (Fig. 6F-G, I). Immunofluorescent staining of tumor tissues from the orthotopic model revealed that Liensinine induced metabolic reprogramming in vivo, as evidenced by increased expression of oxidative phosphorylation-related markers (Fig. 6H). Further fluorescent staining of CD31, CD206, and CD86 confirmed the polarization of macrophages and provided additional evidence of reduced vascular density and altered macrophage populations within the tumor microenvironment (Fig. 6J). PD-L1, as an immune checkpoint, plays a crucial role in tumor immunity and is associated with immune therapy. Our RNA sequencing results showed that Liensinine significantly reduced the transcriptional levels of PD-L1. Studies have also suggested that AMPK and HIF-1α regulate PD-L1 [28–30], Our results demonstrated that Liensinine markedly decreased the protein levels of PD-L1. The use of an AMPK inhibitor or the overexpression of HIF-1α reversed the Liensinine-induced reduction in PD-L1 levels, suggesting that Liensinine modulates PD-L1 expression through the AMPK-HIF-1α axis (Figure S5A-B). To explore whether Liensinine can enhance the effects of immunotherapy, we investigated its combinatorial action with immunotherapy in an orthotopic model. Our results demonstrated that the combination of Liensinine with PD-L1 inhibitors effectively suppressed the growth of orthotopic hepatocellular carcinoma tumors (Fig. 6L). Furthermore, this combination therapy significantly increased the proportion of CD8 + T cells within tumor-infiltrating immune cells (Fig. 6M and Figure S6A). Immunofluorescence analysis further confirmed that the combined treatment promoted an increased proportion of M1 macrophages within the tumor microenvironment (Fig. 6N). ELISA analysis of tumor-associated cytokines revealed that the combination of Liensinine and PD-L1 inhibitors significantly reduced levels of pro-tumorigenic cytokines, while concurrently elevating the levels of cytokines associated with tumor suppression and cytotoxicity (Fig. 6O). Collectively, these results provide compelling evidence that Liensinine exerts potent antitumor effects in vivo by inhibiting tumor growth, reducing angiogenesis, and promoting macrophage polarization towards an anti-tumor M1 phenotype. Liensinine combined with immunotherapy can better inhibit tumor progression. This suggests that Liensinine holds significant potential as a therapeutic agent for liver cancer.

### Effects of liensinine combined with radiotherapy and immunotherapy on the tumor immune microenvironment

Recent studies have confirmed the important role of radiotherapy in the treatment of HCC [31, 32]. Previous studies have demonstrated that radiotherapy enhances immune cell infiltration into the tumor microenvironment, potentially augmenting the effectiveness of immunotherapy [33]. Based on this, we examined the synergistic effects of Liensinine, radiotherapy, and immunotherapy in a combined treatment regimen.

**Fig. 7:**
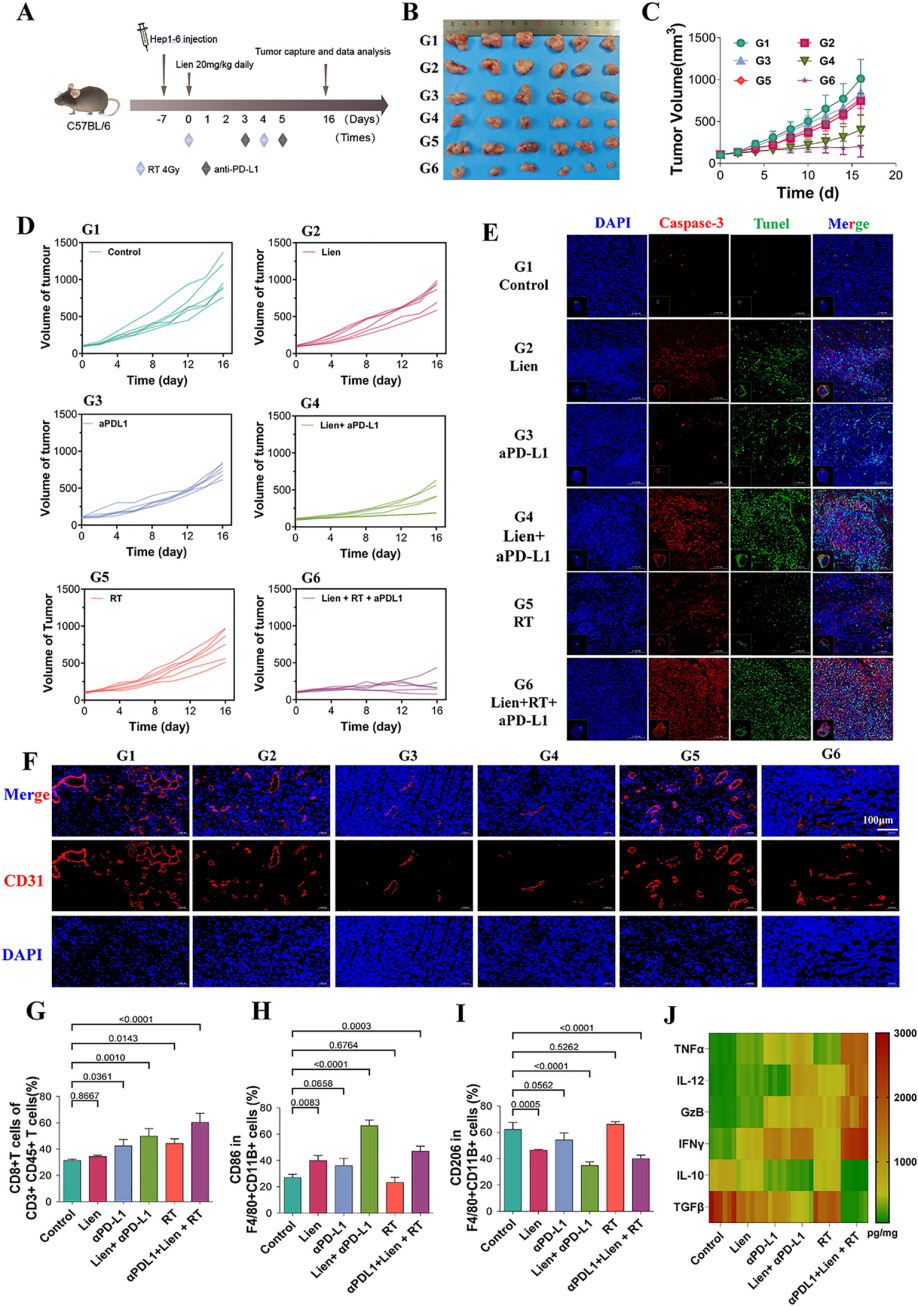
(**A**) The dosing schedule diagram. (**B**) Tumor tissues were harvested and photographed on day 16 post-treatment. (**C**, **D**) Tumor growth curves of mice in each treatment group. (**E**) Immunofluorescence staining of caspase-3 (red) and TUNEL (green) in tumor tissues. (**F**) Immunofluorescence staining for CD31 (red) expression across treatment groups. (**G**) Flow cytometry analysis of the percentage of CD8 + T cells among CD3 + CD45 + T cells across treatment groups. (**H**, **I**) Flow cytometry analysis of the percentage of CD86 + M1 macrophages and CD206 + M2 macrophages within F4/80 + CD11b + macrophages. (**J**) ELISA was used to measure cytokine levels in tumor tissue homogenates. For the analyses in (**G**, **H**, **I**) (*n* = 5 independent samples), Student′s t test was conducted

Our results showed that both Liensinine and immunotherapy alone were effective in inhibiting tumor growth. However, the combination of Liensinine, radiotherapy, and immunotherapy resulted in a more pronounced suppression of tumor growth compared to any single treatment (Fig. 7A-D). Immunofluorescence staining of tumor sections revealed that the combination of Liensinine, radiotherapy, and immunotherapy enhanced the expression of apoptotic markers, such as caspase-3 and TUNEL, indicating increased tumor cell apoptosis (Fig. 7E). Furthermore, CD31 staining showed that Liensinine combined with immunotherapy significantly reduced tumor vascular density, a reduction not observed with radiotherapy alone, suggesting that Liensinine influences tumor vasculature in a way that enhances immunotherapy efficacy (Fig. 7F). Flow cytometric analysis of tumor-infiltrating immune cells demonstrated that Liensinine, when combined with immunotherapy, promoted the infiltration of CD8 + T cells into the tumor. This effect was further amplified when combined with radiotherapy, resulting in a higher proportion of CD8 + T cells infiltrating the tumor (Fig. 7G and Figure S6B). Immunofluorescence staining also confirmed the increased infiltration of CD8 + T cells within the tumor tissue (Figure S7). In addition to T cells, Liensinine and immunotherapy together promoted macrophage polarization toward the M1 phenotype, whereas radiotherapy alone induced polarization toward the M2 phenotype (Fig. 7H-I and Figure S6C-D). Cytokine analysis of tumor tissues revealed that the combination of Liensinine and immunotherapy significantly elevated levels of pro-inflammatory cytokines, including TNF-α, IL-12, IFN-γ, and granzyme B, while reducing the levels of immunosuppressive cytokines, such as IL-10 and TGF-β (Fig. 7J). The biosafety of combined therapy is also evaluated (Figure S8). These findings highlight that Liensinine enhances the efficacy of immunotherapy by modulating PD-L1 expression and reshaping the tumor immune microenvironment. The addition of radiotherapy further potentiated these effects, providing a promising strategy to improve the therapeutic outcome of cancer treatment.

## Discussion

Metabolic reprogramming is a hallmark of cancer, influencing tumor progression, treatment resistance, and immune evasion [34, 35]. Tumor cells frequently shift from oxidative phosphorylation to glycolysis, a phenomenon known as the Warburg effect. Recent research underscores the pivotal role of metabolic pathways in regulating various aspects of tumor biology, including cell growth, migration, and angiogenesis [36]. This study explores how Liensinine, a promising anticancer compound, modulates these processes in hepatocellular carcinoma (HCC).

Our study demonstrates that Liensinine disrupts this glycolytic switch by promoting oxidative phosphorylation through the activation of the AMPK-HIF-1α axis. Liensinine-treated HCC cells showed reduced glucose uptake, decreased extracellular lactate production, and increased oxidative phosphorylation, resulting in heightened reactive oxygen species (ROS) generation. This metabolic reprogramming not only inhibits tumor cell growth but also induces oxidative stress that can lead to DNA damage and apoptosis, providing a mechanistic basis for Liensinine’s antitumor activity [37], these findings align with recent studies suggesting that targeting the metabolic dependencies of cancer cells is a promising therapeutic approach [38].

The AMPK-HIF-1α axis plays a central role in regulating cancer metabolism, particularly under metabolic stress [39, 40]. AMPK activation promotes catabolic processes such as oxidative phosphorylation while inhibiting anabolic processes like glycolysis through downregulation of HIF-1α and glycolysis related regulators of HK2, PKM [41–43]. Our experiments demonstrate that Liensinine activates AMPK, as evidenced by increased AMPK phosphorylation, and concurrently suppresses HIF-1α expression. The reversal of Liensinine’s effects by AMPK inhibition or HIF-1α overexpression confirms that Liensinine’s antitumor activity is mediated through the AMPK-HIF-1α pathway. Enhanced oxidative phosphorylation induced by Liensinine results in high ROS levels, which likely contribute to oxidative stress and damage DNA, thus impairing tumor cell proliferation and growth [44].

Metabolic reprogramming also profoundly affects the tumor microenvironment, particularly in terms of angiogenesis and immune cell function [45, 46]. Lactate, a key glycolytic byproduct, promotes angiogenesis by stimulating VEGF production and supporting endothelial cell proliferation [47]. Our data indicate that Liensinine treatment reduces VEGF levels in HCC cells, likely due to its inhibition of glycolysis and subsequent lactate production. In vivo, Liensinine significantly decreases tumor vascular density, reinforcing the notion that Liensinine suppresses angiogenesis through its impact on tumor metabolism. In addition to angiogenesis, lactate is crucial in regulating immune cell function, particularly the polarization of tumor-associated macrophages (TAMs) [26, 48]. Tumor cells often drive macrophages from the pro-inflammatory, antitumor M1 phenotype to the anti-inflammatory, protumor M2 phenotype [49]. Lactate mediates this transition, contributing to an immunosuppressive microenvironment that allows tumors to evade immune surveillance [50]. Our study shows that Liensinine treatment promotes M1 macrophage polarization and inhibits M2 polarization, both in vitro and in vivo. These results suggest that Liensinine, by modulating metabolic pathways, not only impacts tumor cell metabolism but also reshapes the immune environment to a more antitumor phenotype.

The ER plays a crucial role in maintaining cellular homeostasis, and under conditions of metabolic or oxidative stress, the accumulation of misfolded proteins in the ER triggers the unfolded protein response (UPR), which can activate key stress-related pathways, including AMPK [51]. Our study demonstrates that Liensinine treatment leads to significant ER stress, as evidenced by increased protein levels of CHOP and ATF4, markers of UPR activation. We also show that the ER stress-induced activation of AMPK plays a critical role in the suppression of HIF-1α and subsequent downregulation of glycolysis-related genes such as HK2 and PDK1. By promoting AMPK activation through ER stress, Liensinine induces a metabolic switch that favors oxidative phosphorylation and disrupts the Warburg effect.

One of the key findings of our study is the modulation of PD-L1 expression by Liensinine. PD-L1, an immune checkpoint protein, is often upregulated in cancer cells to inhibit the activity of cytotoxic T lymphocytes (CTLs) and evade immune surveillance [52]. Recent research indicates that metabolic pathways, including those regulated by AMPK, can influence PD-L1 expression [28, 53]. Consistent with these findings, we observed that Liensinine significantly reduced the transcriptional and protein levels of PD-L1 in HCC cells, which likely contributes to enhanced immune responses. The reduction in PD-L1 expression was shown to be mediated by AMPK activation, further linking Liensinine’s metabolic reprogramming effects to its immune modulatory activity. This suggests that Liensinine not only targets tumor metabolism but also acts as an immune checkpoint modulator, making it a promising adjunct to immunotherapy. When combined with PD-L1 inhibitors, Liensinine treatment results in increased CD8 + T cell infiltration and enhanced M1 macrophage polarization. The shift toward M1 polarization in the presence of Liensinine is a promising indication that Liensinine can reshape the immune microenvironment in favor of antitumor immunity. Moreover, radiotherapy, a common clinical treatment, often used in combination with immunotherapy, promotes immune cell infiltration into the tumor microenvironment [54]. Our study reveals that while radiotherapy increases CD8 + T cell infiltration, it also induces M2 macrophage polarization and does not reduce tumor vascular density. The combination of Liensinine with immunotherapy and radiotherapy markedly suppresses tumor progression, suggesting that Liensinine could be a valuable adjunct in HCC treatment by enhancing the effects of both immunotherapy and radiotherapy.

Despite the promising findings from our study, several limitations should be acknowledged. First, although we demonstrated Liensinine’s efficacy in both in vitro and in vivo models, further investigation is needed to determine its clinical applicability, including its pharmacokinetics, bioavailability, and potential toxicity in humans. Additionally, while we identified the AMPK-HIF-1α axis as a key regulator of Liensinine’s effects, the precise molecular interactions between Liensinine and these pathways remain to be elucidated. Further studies are required to dissect the downstream signaling events and to assess whether other pathways may also contribute to Liensinine’s antitumor activity. Lastly, while we focused on macrophages and CD8 + T cells in our analysis of the immune microenvironment, the effects of Liensinine on other immune cells, such as regulatory T cells or dendritic cells, warrant further exploration.

## Conclusion

In summary, Liensinine demonstrates potent antitumor effects in HCC by modulating metabolic reprogramming, inducing ER stress, enhancing immune responses, and reshaping the TME. The combination of Liensinine with immunotherapy and radiotherapy further enhances its therapeutic efficacy, making it a promising candidate for future clinical trials. Importantly, our study introduces ER stress as a novel mechanism underlying Liensinine’s anticancer effects, providing new insights into the complex interplay between metabolism, immune modulation, and cellular stress responses. Further research is needed to explore the clinical applicability of Liensinine, its pharmacokinetics, and its potential side effects in human patients.

## Electronic supplementary material

Below is the link to the electronic supplementary material.

Supplementary Material 1

Supplementary Material 2

## Data Availability

No datasets were generated or analysed during the current study.

## Abbreviations

HCC: Hepatocellular carcinoma
PBMCs: Peripheral blood mononuclear cells
ER: Endoplasmic reticulum
AMPK: Adenosine 5‘-monophosphate (AMP)-activated protein kinase
Hif-1a: Hypoxia Inducible Factor 1 Alpha
PD-L1: Programmed Cell Death-Ligand 1
TME: Tumor microenvironment
HUVEC: Human umbilical vein endothelial cells
DMEM: Dulbecco’s Modified Eagle Medium
CCK-8: Cell Counting Kit-8
PI: Propidium Iodide
RNA: seq-RNA Sequencing
WB: Western Blot
IHC: Immunohistochemistry
qRT-PCR: Quantitative Real-Time PCR
IF: Immunofluorescence
GSEA: Gene Set Enrichment Analysis
DEGs: Differentially expressed genes
DMSO: Dimethylsulfoxide
ECAR: Extracellular acidification rate
OCR: Oxygen consumption rate
VEGF: Vascular Endothelial Growth Factor
TNFa: Tumor Necrosis Factor alpha
UPR: Unfolded protein response
