# Supplementary material for: Liensinine reshapes the immune microenvironment and enhances immunotherapy by reprogramming metabolism through the AMPK-HIF-1α axis in hepatocellular carcinoma
Source: J Exp Clin Cancer Res. 2025 Jul 15;44:208. doi: 10.1186/s13046-025-03477-6 (PMC12261578; doi:10.1186/s13046-025-03477-6)
Supplement: Supplementary file 1 — Supplementary Material 1 [file 13046_2025_3477_MOESM1_ESM.docx]

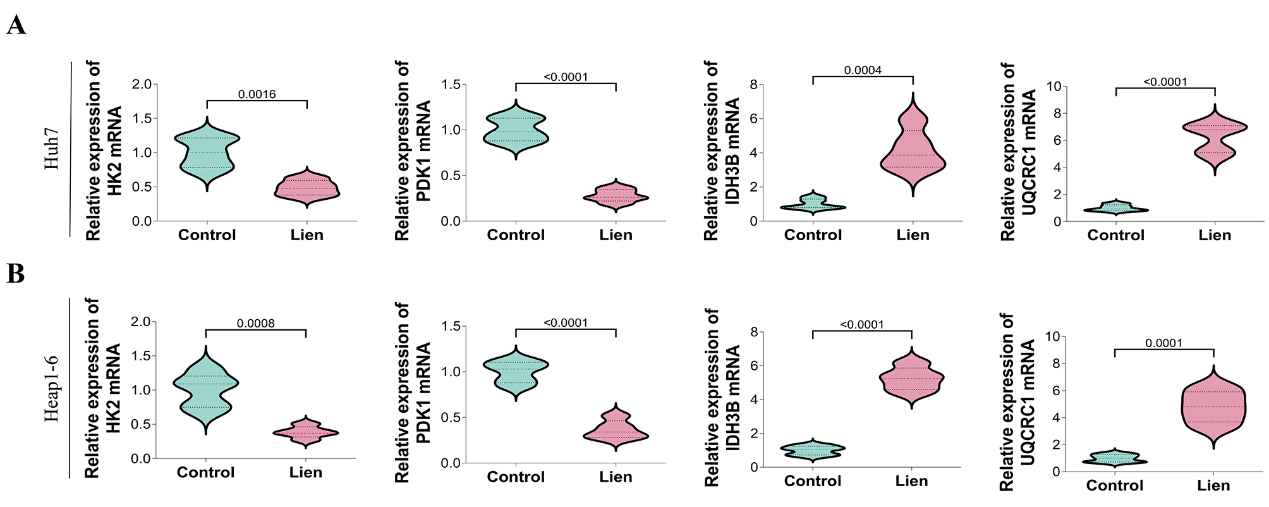


FigureS1: The effect of Liensinine on the RNA expression of glucose metabolism-related genes in two types of liver cancer cells.


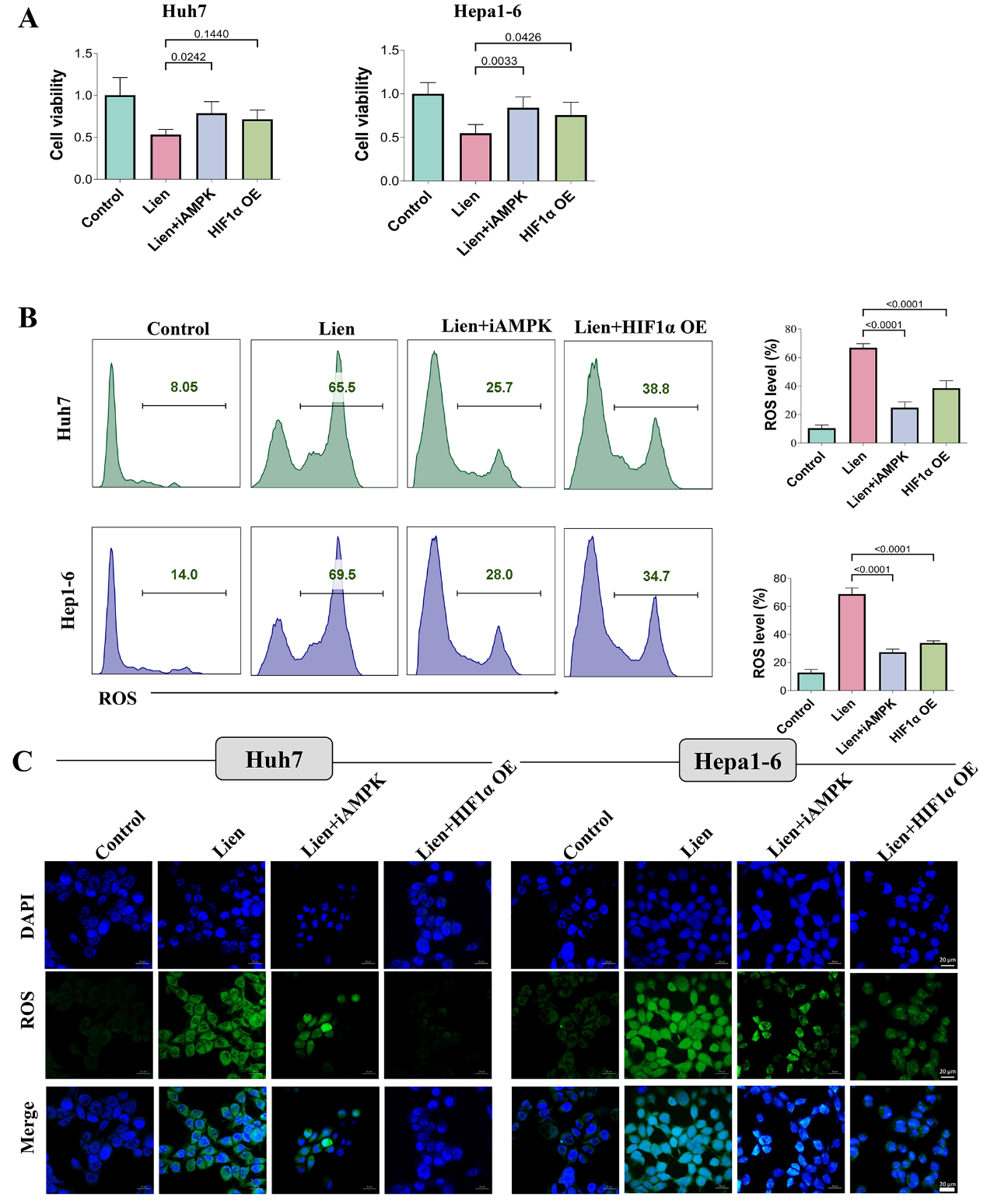


FigureS2: (A) Changes in the viability of liver cancer cells under different treatments, (B) Flow cytometric detect the levels of ROS in two types of liver cancer cells under different treatment. (C) Immunofluorescence staining detect the levels of ROS in two types of liver cancer cells under different treatment.


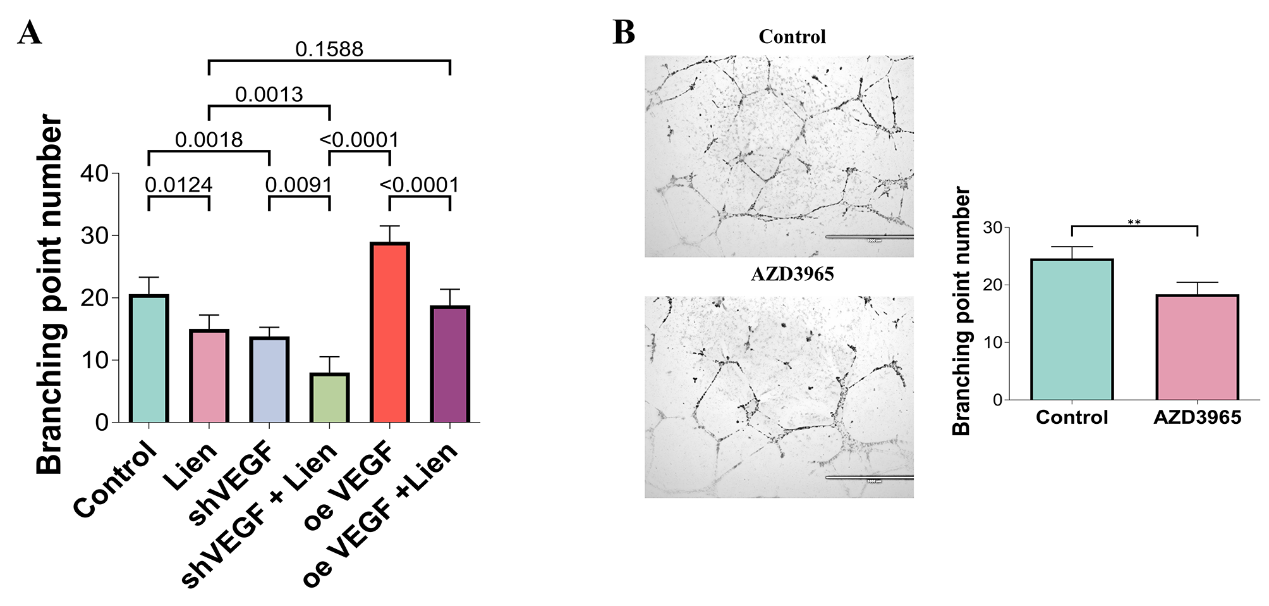


FigureS3: (A) Quantification of the tube formation assay (Figure3 M), (B) The effect of the lactate transporter inhibitor on tube formation and its quantification.


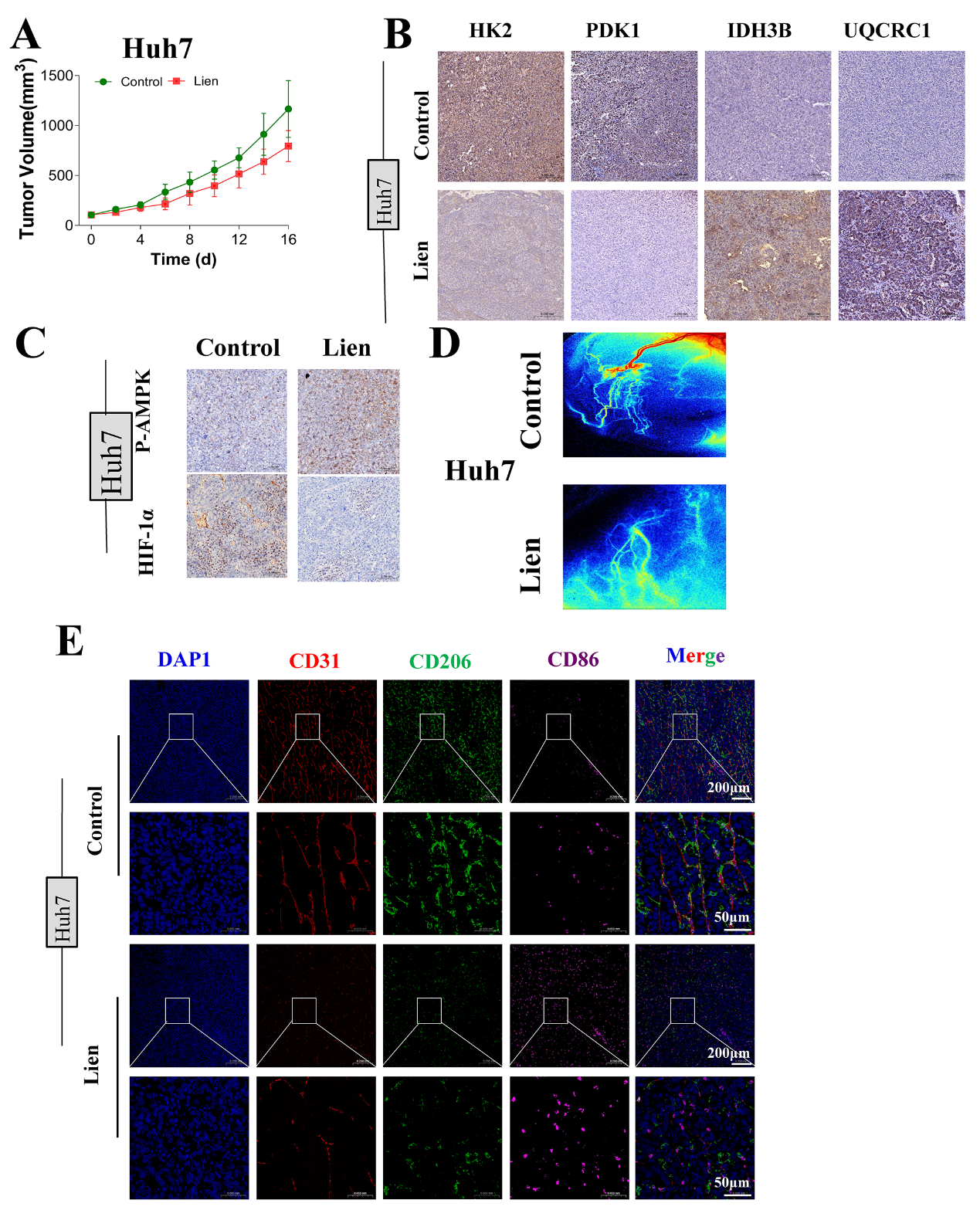


FigureS4: (A) Growth curves of Huh7 xenograft tumors. (B) Immunohistochemical analysis of HK2, PDK1, IDH3B, and UQCRC1 expression in tumor tissues. (C) Immunohistochemical detection of P-AMPK and Hif-1a expression in HuH7 tumor tissues. (D) Immunofluorescence staining to assess the expression of CD31 (Red), CD206 (Green), and CD86 (purple) in tumor tissues. (E) In vivo angiogenesis of tumors observed through laser speckle imaging.


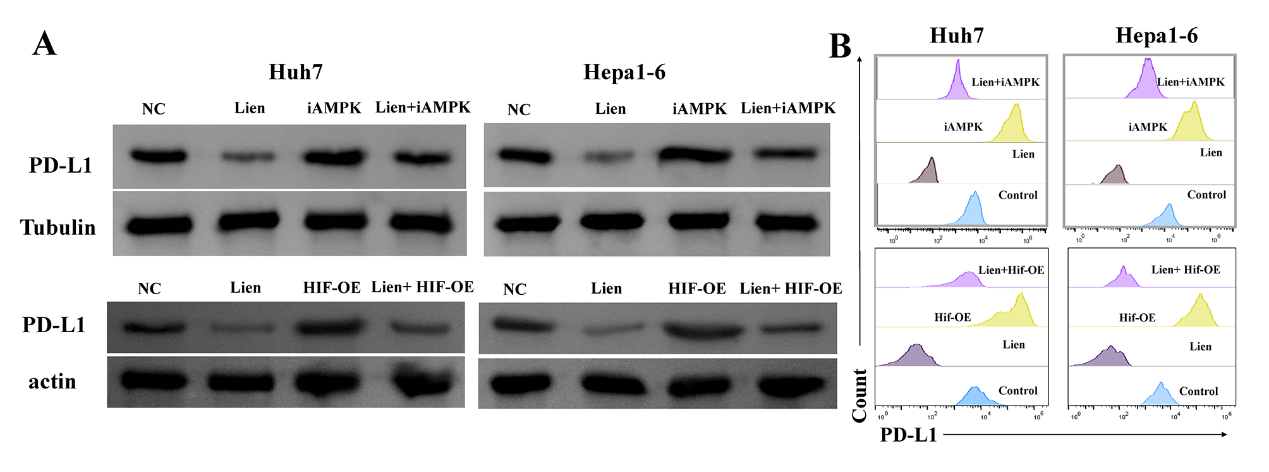


FigureS5: (A) Western blot and (B) flow cytometry was used to assess PDL1 expression in tumor cells after different treatments.


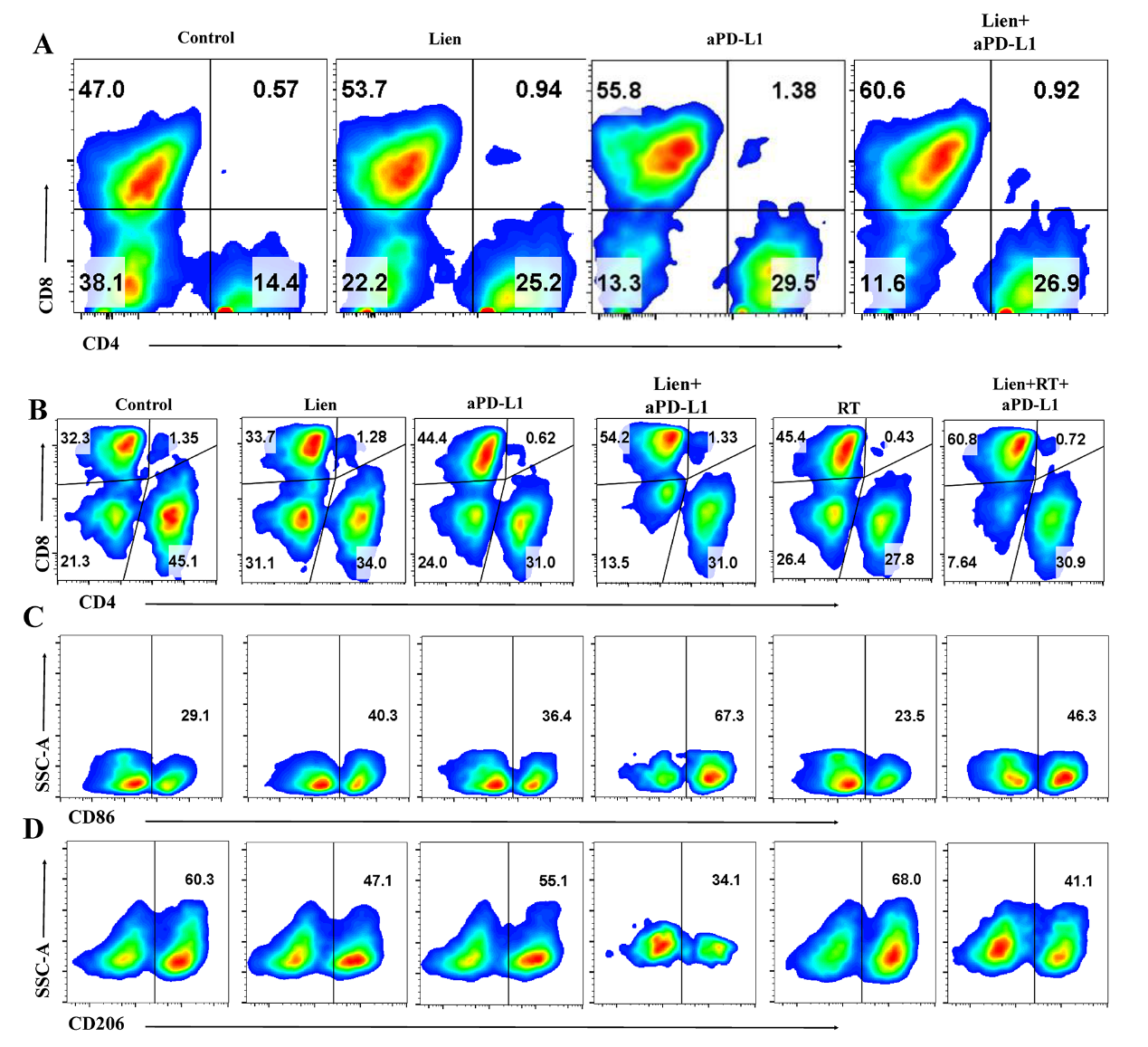


FigureS6: (A) Flow cytometry analysis of the percentage of CD8+ T cells among CD3+CD45+ T cells across treatment groups in orthotopic liver cancer transplantation tumor model. (B) Flow cytometry analysis of the percentage of CD8+ T cells among CD3+CD45+ T cells across treatment groups. (C, D) Flow cytometry analysis of the percentage of CD86+ M1 macrophages and CD206+ M2 macrophages within F4/80+CD11b+ macrophages.


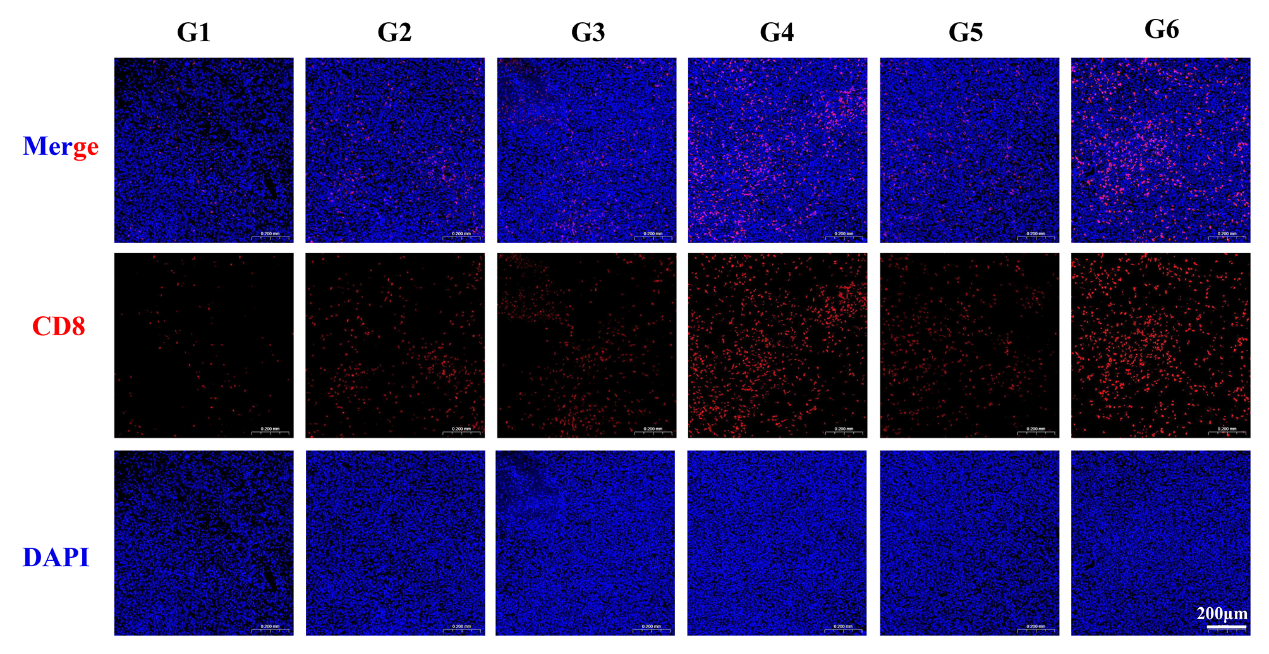


FigureS7: Fluorescence staining results of CD8 in tumor tissues under different treatment groups (Figure 7)


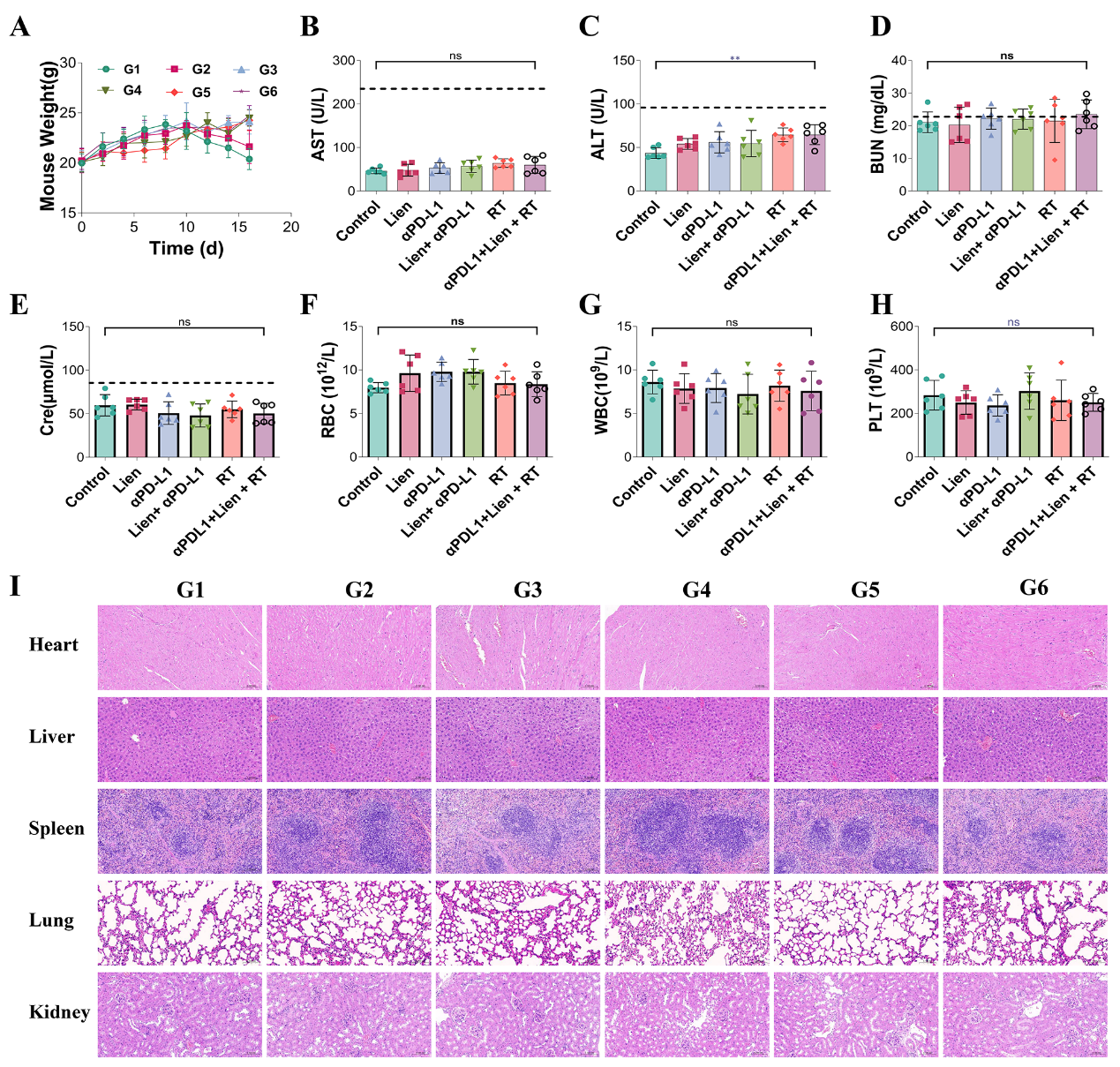


FigureS8: (A) Changes in body weight of mouse during treatment, (B-H) Liver and kidney function and blood cell status of mice in different treatment groups, (I) HE staining of heart, liver, spleen, lung, and kidney tissues in mouse of different treatment groups.
