# Supplementary material for: Liensinine reshapes the immune microenvironment and enhances immunotherapy by reprogramming metabolism through the AMPK-HIF-1α axis in hepatocellular carcinoma
Source: J Exp Clin Cancer Res. 2025 Jul 15;44:208. doi: 10.1186/s13046-025-03477-6 (PMC12261578; doi:10.1186/s13046-025-03477-6)

**Methods**

**Quantitative Real-Time PCR (qRT-PCR)**

To validate the RNA-seq results, qRT-PCR was performed for selected genes, including HK2, PDK1, IDH3B, and UQCRC1. Total RNA was reverse transcribed into cDNA using the PrimeScript RT reagent Kit (Takara). qRT-PCR was conducted using SYBR Green Master Mix (Thermo Fisher) on a StepOnePlus Real-Time PCR System (Applied Biosystems). The relative expression of target genes was normalized to GAPDH as an internal control and analyzed using the 2^-ΔΔCt method.

**Western Blot Analysis**

Protein expression levels were determined by Western blotting. Cells were lysed in RIPA buffer containing protease and phosphatase inhibitors (Sigma-Aldrich). Protein concentrations were determined using a BCA Protein Assay Kit (Thermo Fisher). Equal amounts of protein were separated by SDS-PAGE and transferred to PVDF membranes (Millipore). Membranes were blocked with 5% BSA in TBST and incubated overnight at 4°C with primary antibodies against AMPK, phosphorylated AMPK (P-AMPK), HIF-1α, PD-L1, HK2, PDK1, IDH3B, UQCRC1, and GAPDH (all from [specific manufacturers]). After washing, membranes were incubated with HRP-conjugated secondary antibodies and visualized using ECL substrate (Thermo Fisher). Band intensities were quantified using ImageJ software.

**Transduction and transfection**

The lentiviruses for VEGF knockdown, VEGF overexpression, and HIF-1α overexpression were purchased from Genepharma (Shanghai, China). After incubating the cells with the lentiviruses for 48 hours, total RNA and proteins were extracted to validate gene silencing or overexpression. Subsequently, we identified the cell clones using quantitative real-time PCR (RT-qPCR) and Western blot analysis.

**Immunohistochemistry (IHC)**

Tumor tissues were fixed in formalin, embedded in paraffin, and sectioned at 5 µm thickness. Sections were deparaffinized, rehydrated, and subjected to antigen retrieval using citrate buffer (pH 6.0). After blocking with 5% goat serum, sections were incubated overnight at 4°C with primary antibodies against HK2, PDK1, IDH3B, UQCRC1, HIF-1α, and PD-L1. Sections were then incubated with biotinylated secondary antibodies and developed using the DAB chromogen (Dako). Slides were counterstained with hematoxylin and imaged under a light microscope.

**Immunofluorescence for Cells**

Immunofluorescence staining was performed to evaluate the expression of specific proteins in HUH7 and Hep1-6 cells. Cells were seeded onto glass coverslips in 24-well plates at a density of 2 × 10^4 cells per well and allowed to adhere overnight. After treatment with Neferine (30 µM) or control, the cells were washed with phosphate-buffered saline (PBS) and fixed with 4% paraformaldehyde for 15 minutes at room temperature. After washing with PBS, the cells were permeabilized with 0.3% Triton X-100 for 10 minutes and blocked with 5% bovine serum albumin (BSA) for 1 hour at room temperature.

Primary antibodies against target proteins (p-AMPK, HIF-1α, HK2, PDK1, IDH3B, UQCRC1, CHOP, ATF4) were diluted in PBS with 1% BSA and incubated with the cells overnight at 4°C. The next day, the cells were washed with PBS and incubated with fluorophore-conjugated secondary antibodies (Alexa Fluor 488 or 594, Invitrogen) for 1 hour at room temperature in the dark. Nuclei were counterstained with DAPI (Sigma) for 5 minutes. Coverslips were mounted onto glass slides with an anti-fade mounting medium (Thermo Fisher) and examined under a fluorescence microscope (Leica). Images were captured, and protein expression levels were quantified using ImageJ software.

**Immunofluorescence for Tumor Tissue**

For tumor tissue immunofluorescence, tumors harvested from the orthotopic or subcutaneous HCC mouse models were fixed in 4% paraformaldehyde overnight and embedded in optimal cutting temperature (OCT) compound. Cryosections (8 µm thick) were prepared using a cryostat and placed on glass slides. Tissue sections were air-dried for 30 minutes and then fixed in cold acetone for 10 minutes. After fixation, sections were permeabilized with 0.3% Triton X-100 for 10 minutes and blocked with 5% BSA in PBS for 1 hour at room temperature. Primary antibodies against specific markers were diluted in PBS with 1% BSA and incubated with the tissue sections overnight at 4°C. After washing with PBS, the sections were incubated with appropriate fluorophore-conjugated secondary antibodies for 1 hour at room temperature in the dark. Nuclei were counterstained with DAPI for 5 minutes. Sections were mounted with anti-fade mounting medium and visualized under a fluorescence microscope.

**Intracellular ROS levels**

The contents of intracellular ROS in each group of cells were quantified using Reactive Oxygen Species Assay Kit (Byotime), per the manufacturer’s protocol. The frequency of fluorescence-positive cells was quantified by flow cytometry using CellQuest software.

**Measurement of glucose uptake and lactate production**

Glucose uptake and lactate production in each group was tested using the glucose uptake colorimetric assay kit and lactate colorimetric assay kit (Biovision, USA), following the manufacturer’s protocols.

**Reagents and antibodies**

**Information about the antibodies used in the experiments**

| index | Item No | COMPANY |
| --- | --- | --- |
| HK2 | 22915-1-AP | proteintech |
| PDK1 | 19677-1-AP | proteintech |
| IDH3B | ab25901 | abcam |
| UQCRC1 | AF300121 | AiFang biological |
| PD-L1 | 66248-1-Ig | proteintech |
| Tubulin | 66009-1-Ig | proteintech |
| CD11c-BV421 | 565452 | BD Pharmingen |
| CD40-PE | 5537911 | BD Pharmingen |
| CD80-APC | 104713 | Biolegend |
| CD86-BV650 | 564200 | BD Pharmingen |
| CD4-BV605 | 563151 | BD Pharmingen |
| CD8a-APC-Cy7 | 557654 | BD Pharmingen |
| CD274-PE | 558091 | BD Pharmingen |
| CD274-FITC | 558065 | BD Pharmingen |
| IFN-γ-APC | 17–7311-82 | Thermo |

**Information about the kit used in the experiments**

Liensinine SELLCK S9411

Tumor dissociation kit,mouse: Miltenyi, order No. 130-096-730

(TNF-α)ELISA KIT ，Cusabio，CSB-E04740h

(TNF-α)mouse，ELISA KIT ，Cusabio，CSB-E04741m

(IL-12/P40)ELISA Kit ，Cusabio，CSB-E04598h

(IL-12/P40)mouse，ELISA Kit ，Cusabio，CSB-E04600m

(TGF-β1)ELISA kit，Cusabio，CSB-E04725h

Human IL-10 ELISA Kit，abcam，ab46034

Human VEGF ELISA Kit，abcam，ab222510

Mouse TNF alpha ELISA Kit，abcam，ab208348

Mouse IFN gamma ELISA Kit，abcam，ab282874

Mouse IL-10 ELISA Kit，Proteintech，KE10103

Mouse VEGF ELISA Kit，Proteintech，KE10009

lactic acid，CheKine（Abbkine），KTB1100

**Uncropped gel and blot images of western blot**

**
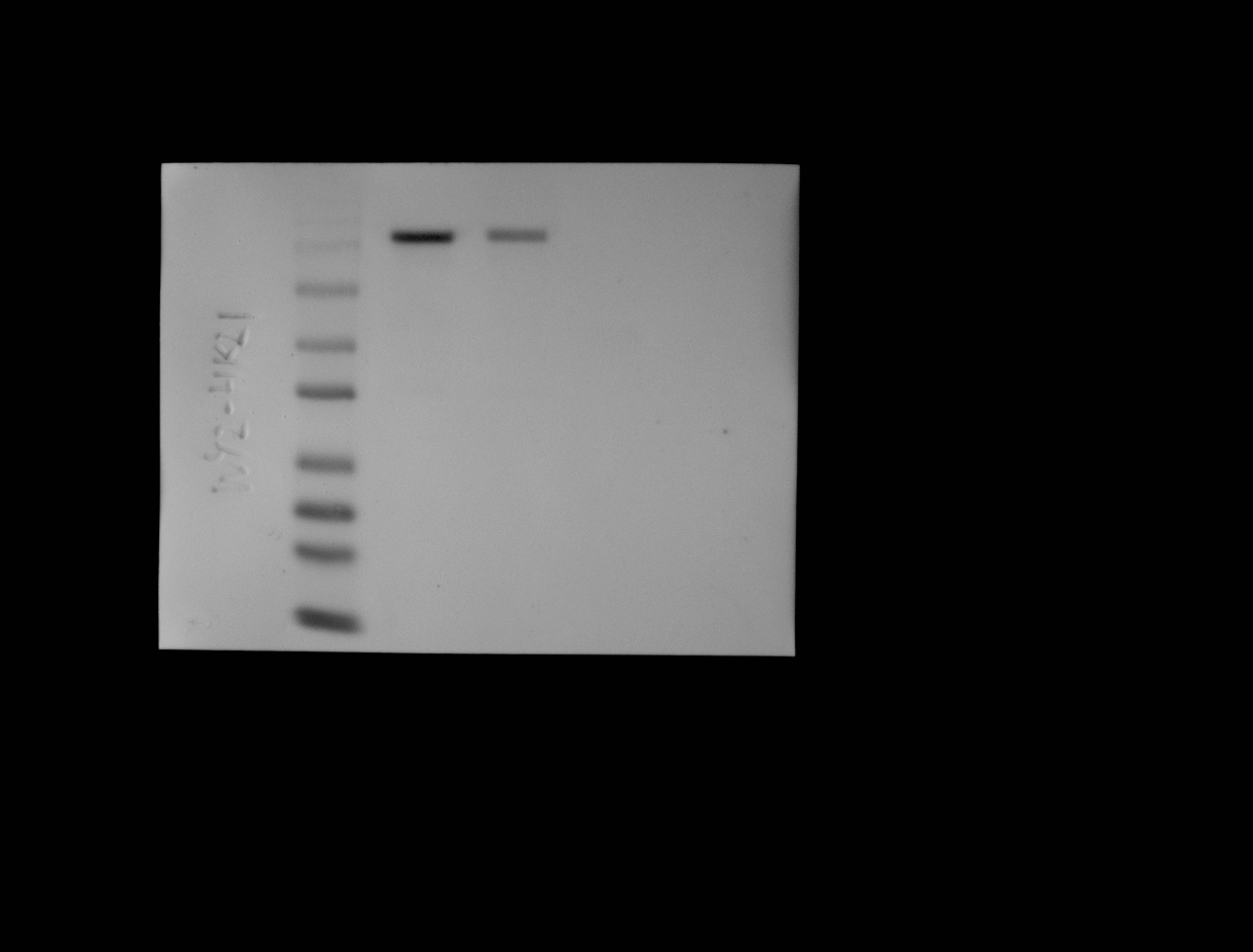
**

**
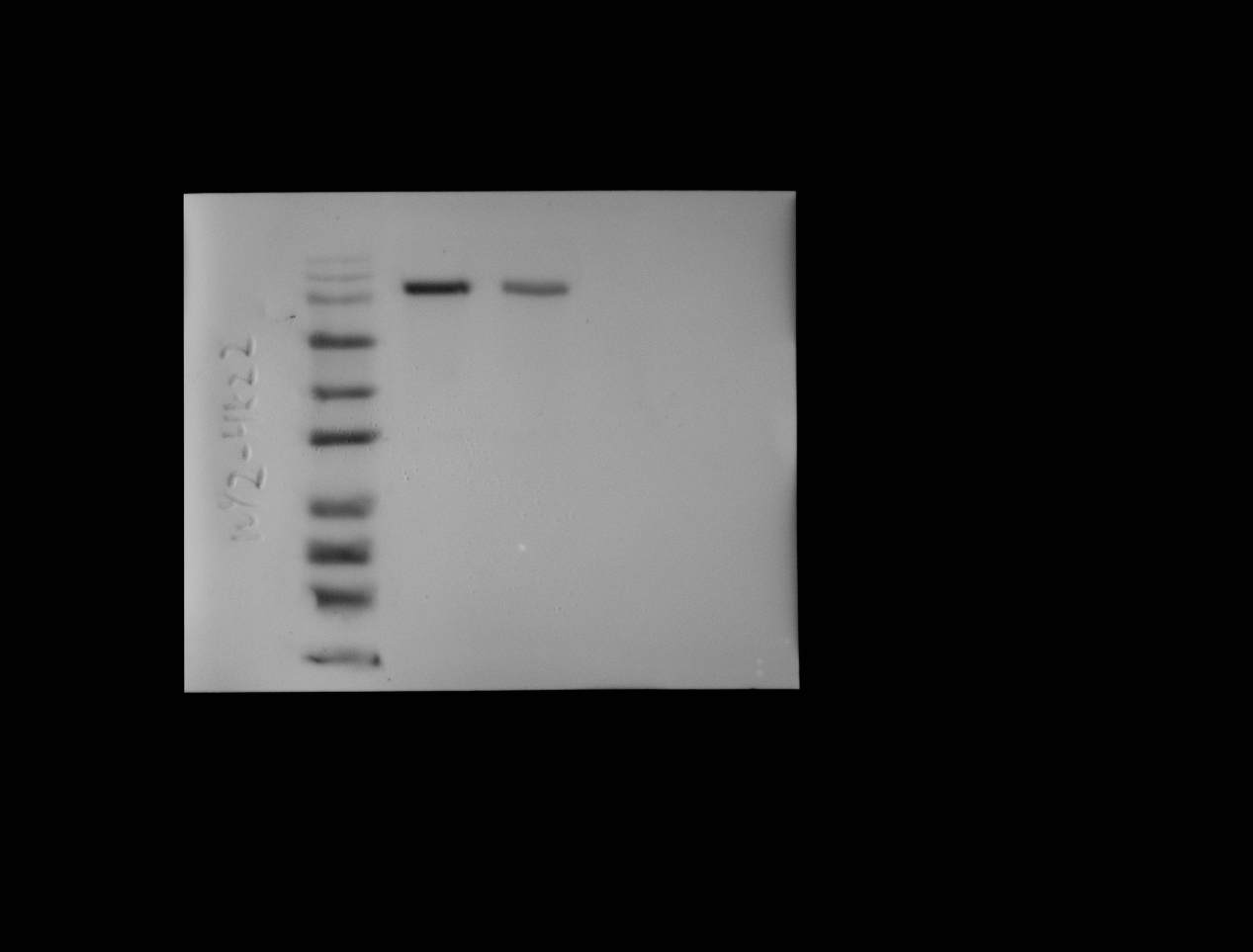
**

**
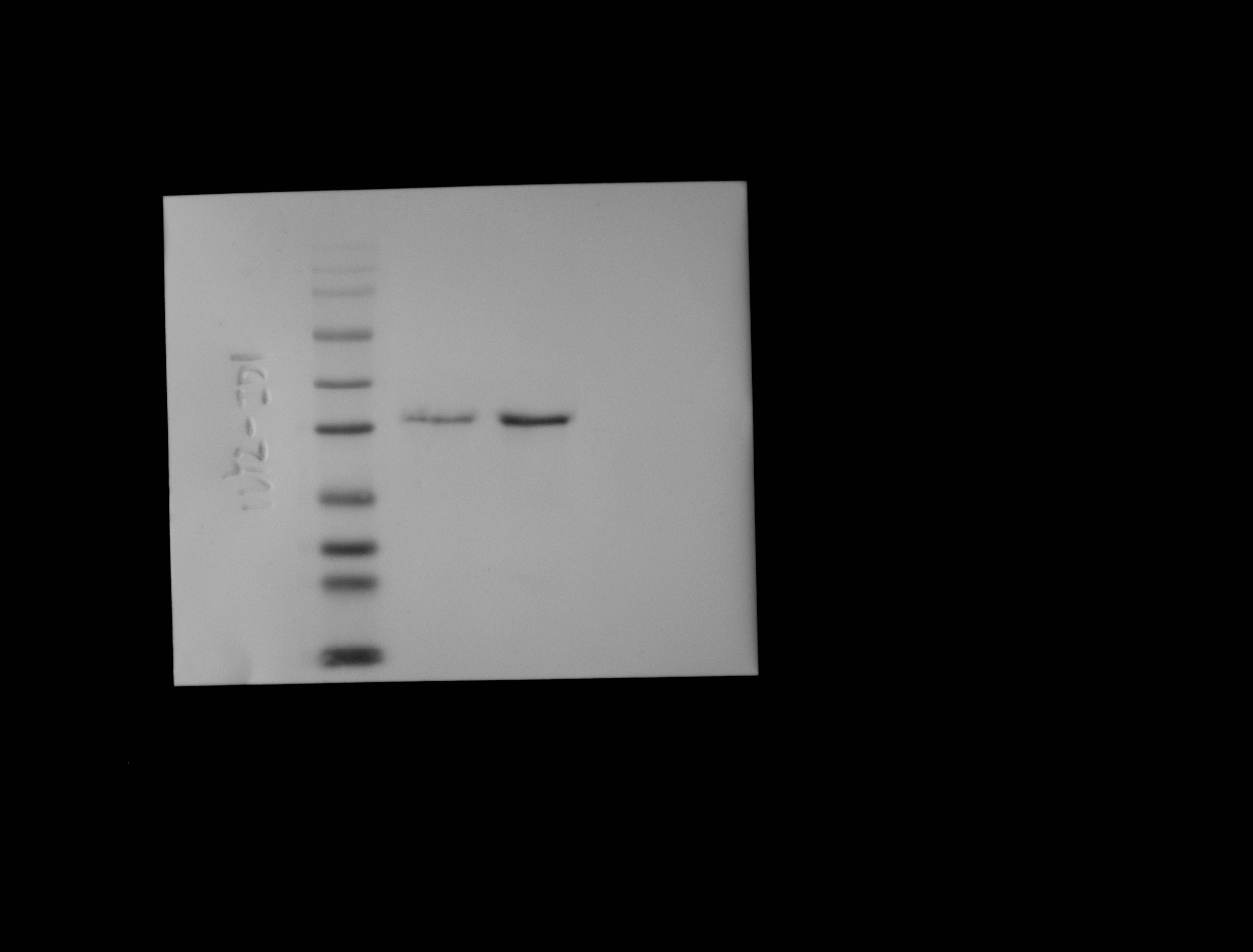
**

**
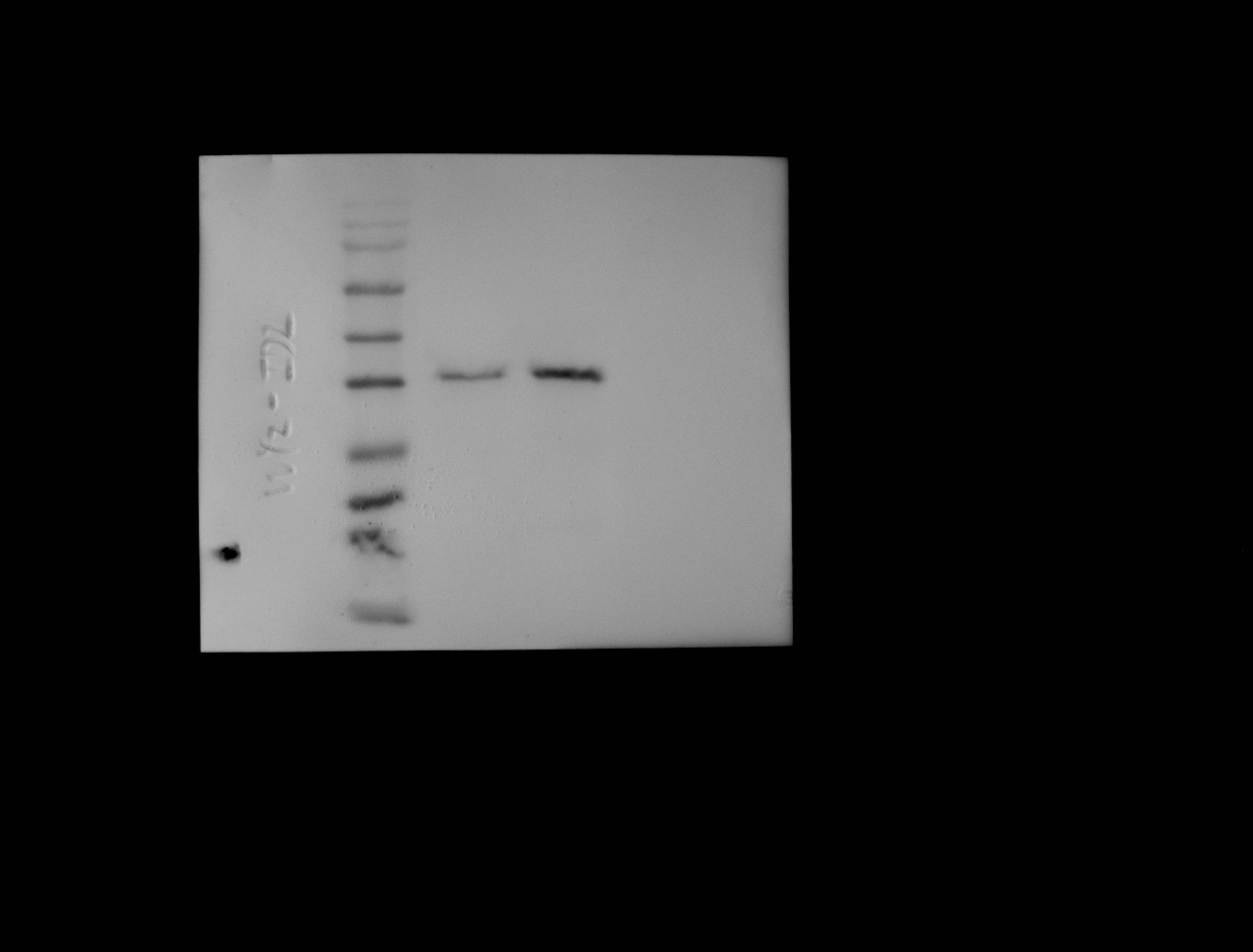
**

**
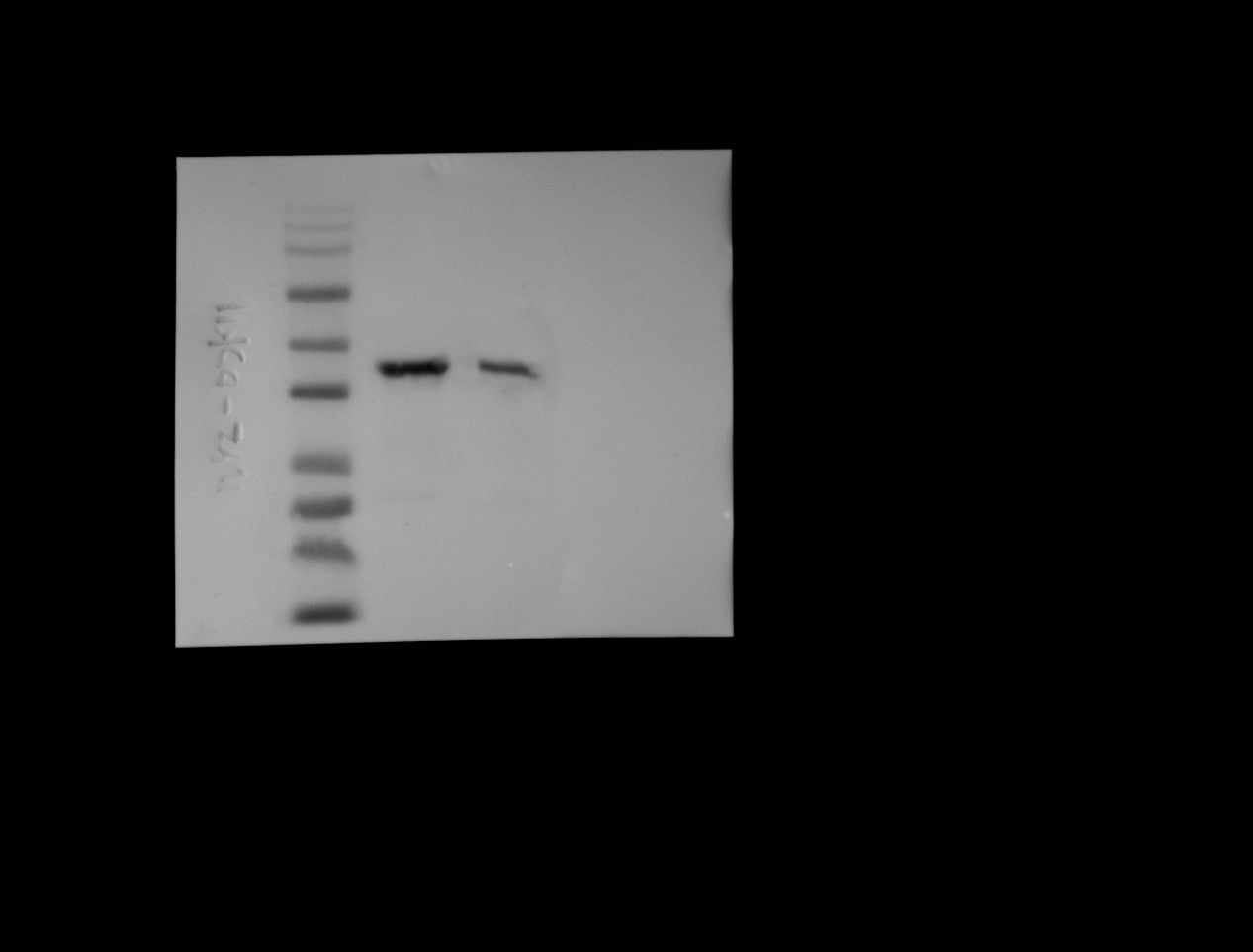
**

**
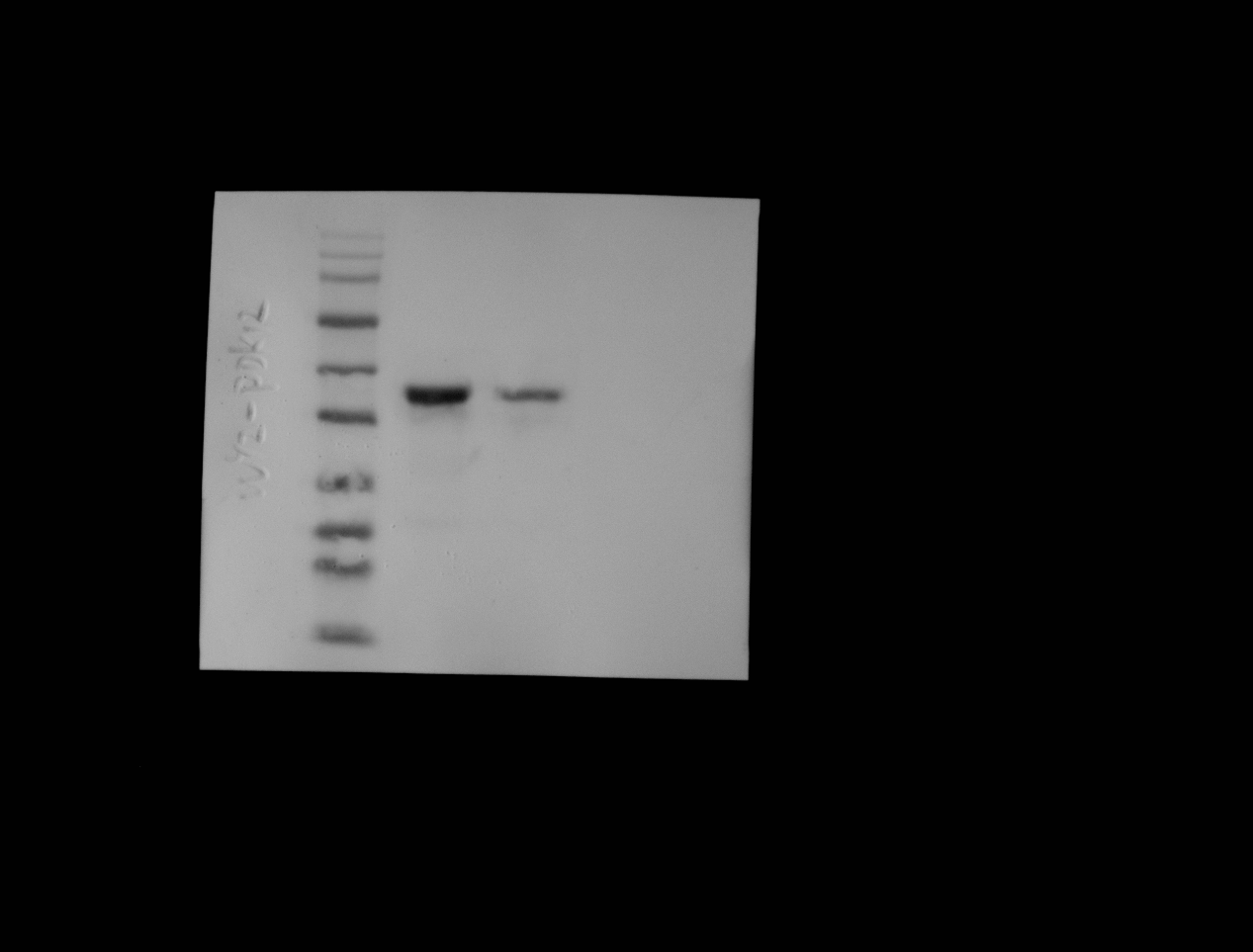
**

**
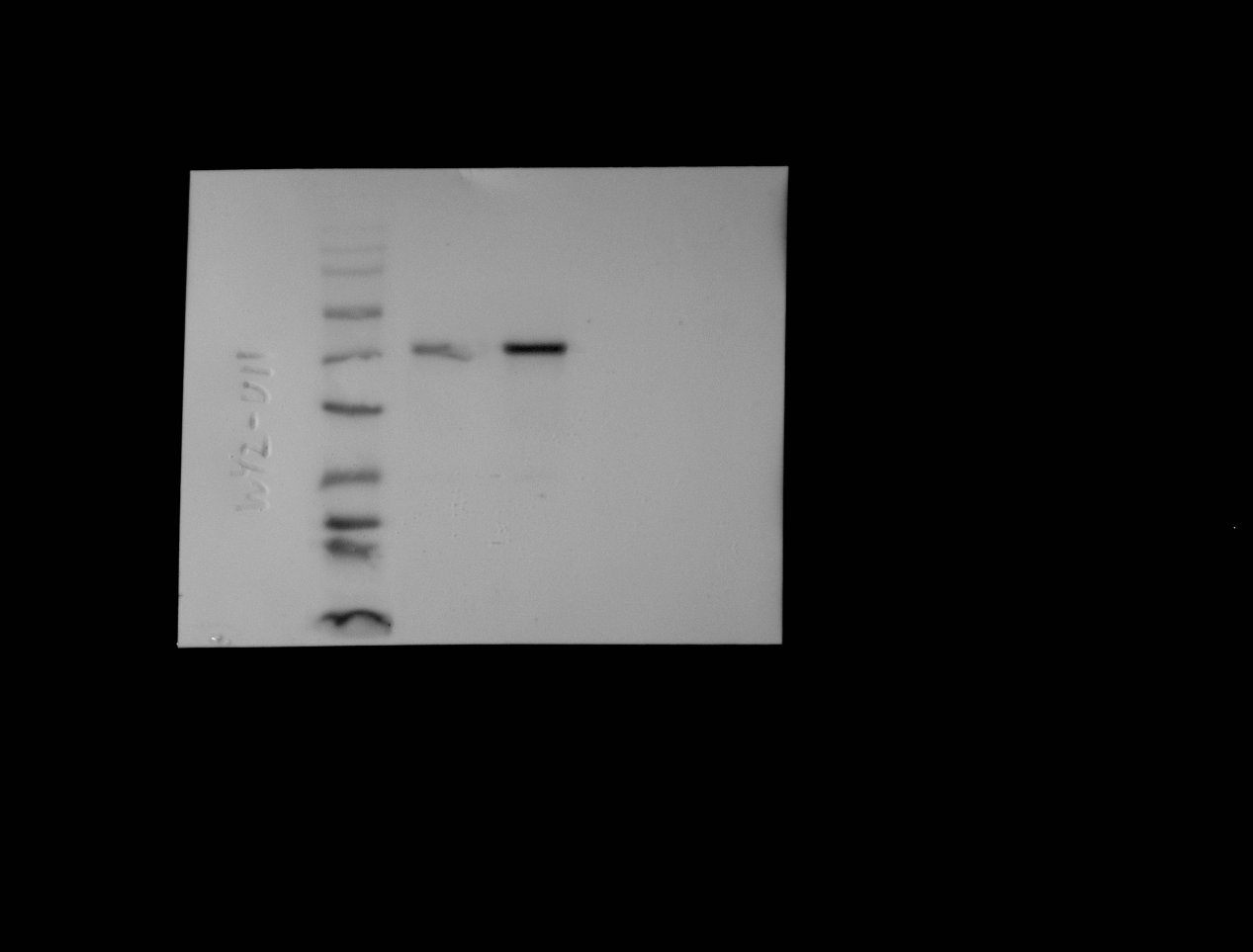
**

**
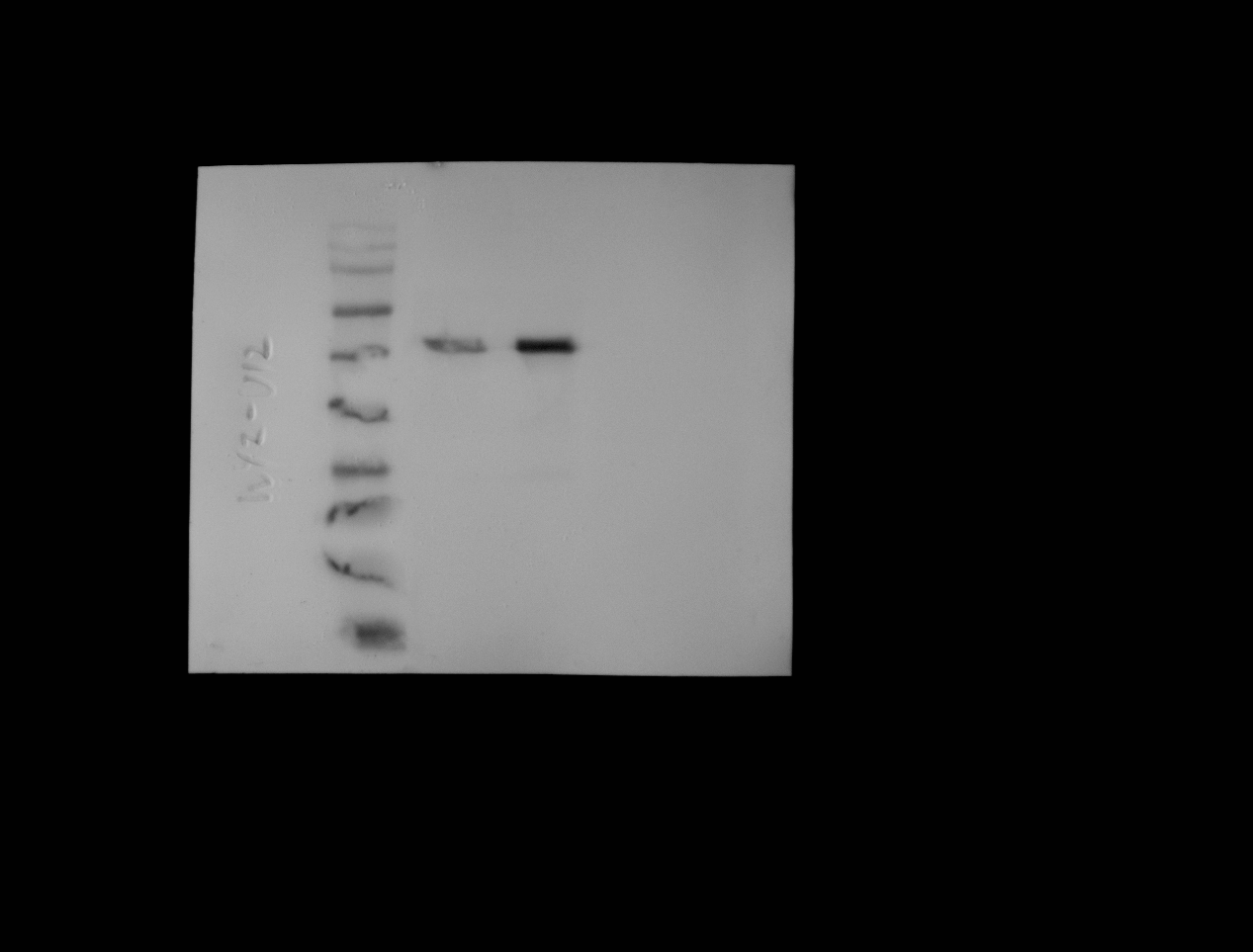
**

**
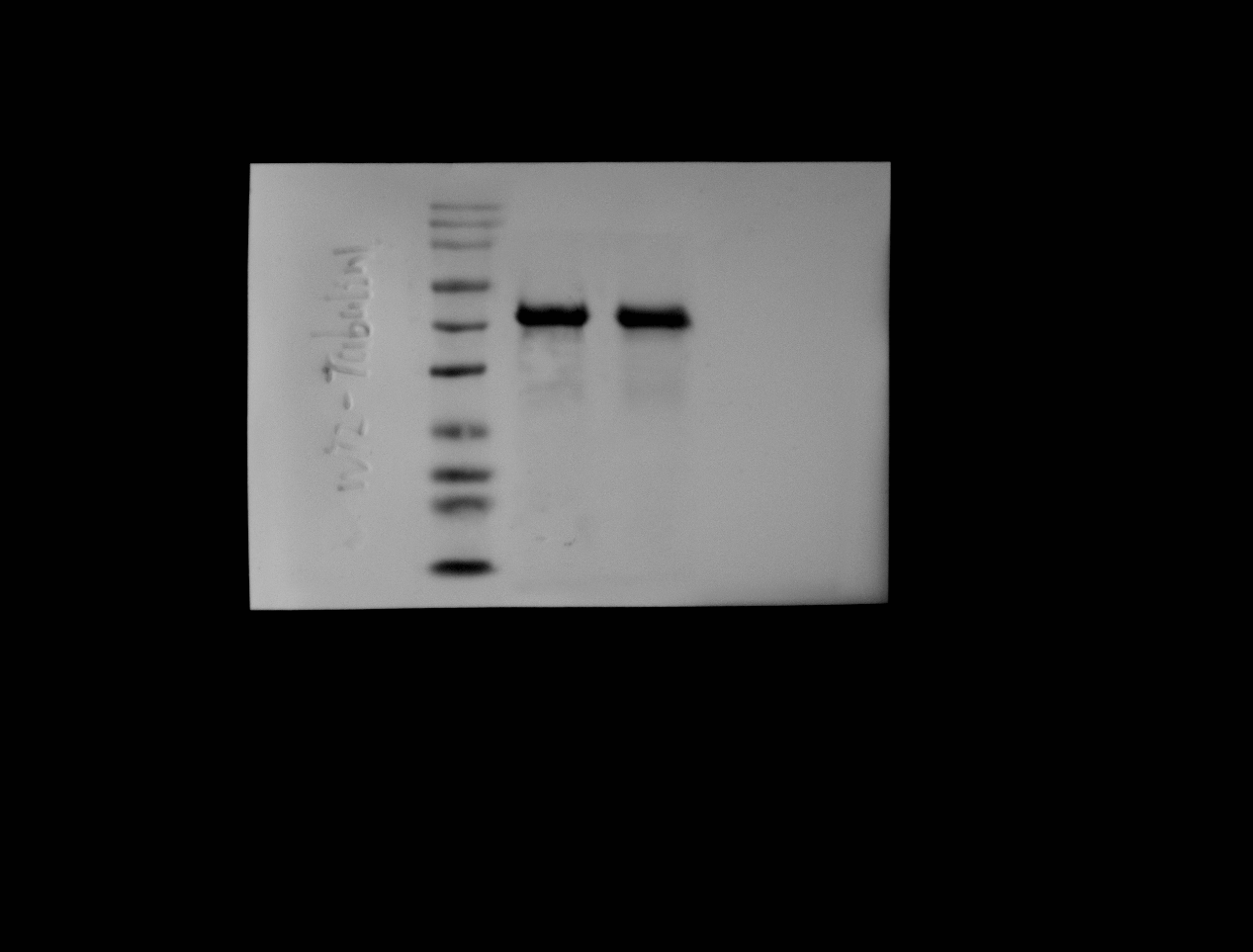
**

**
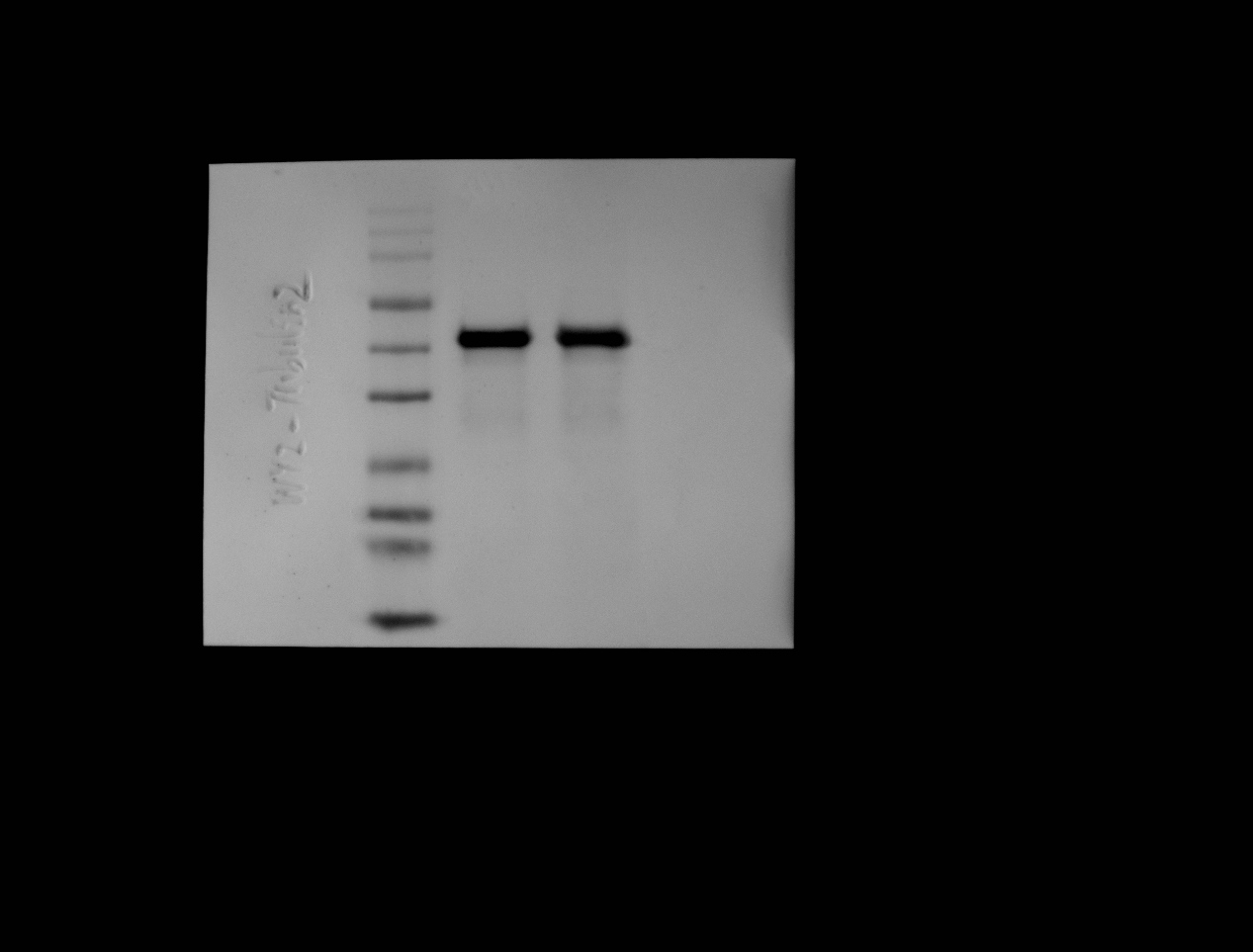
**

**
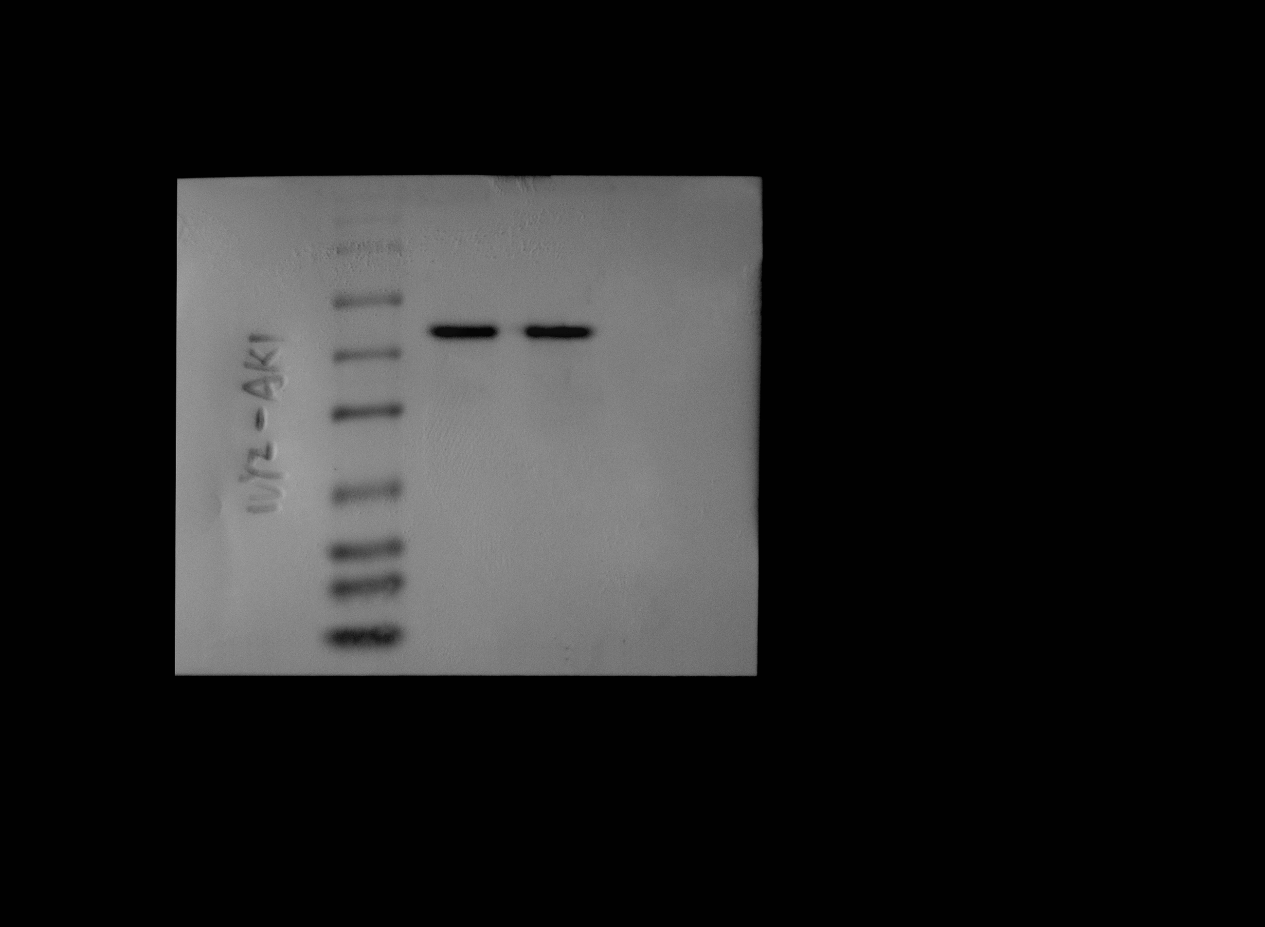
**

**
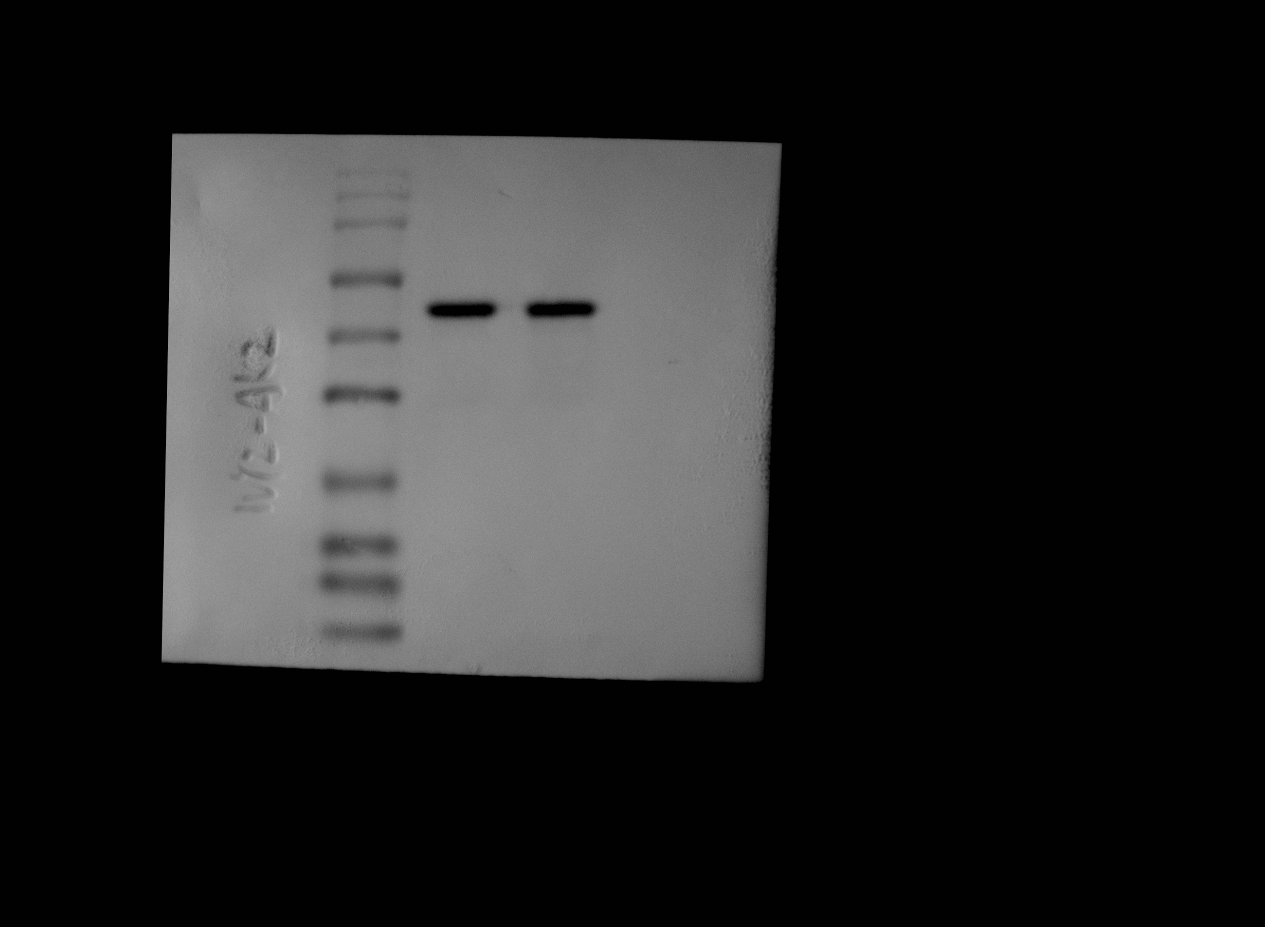
**

**
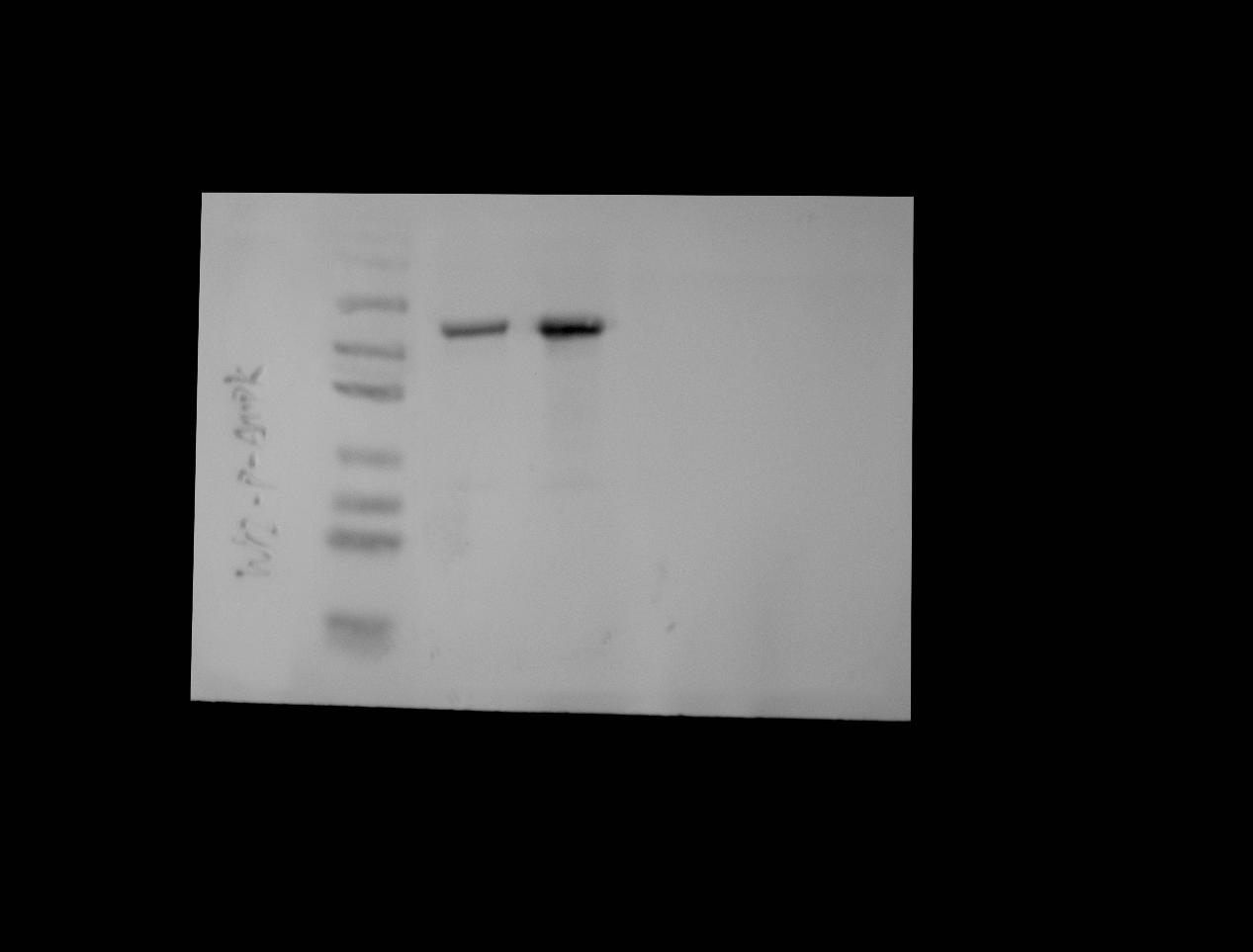
**

**
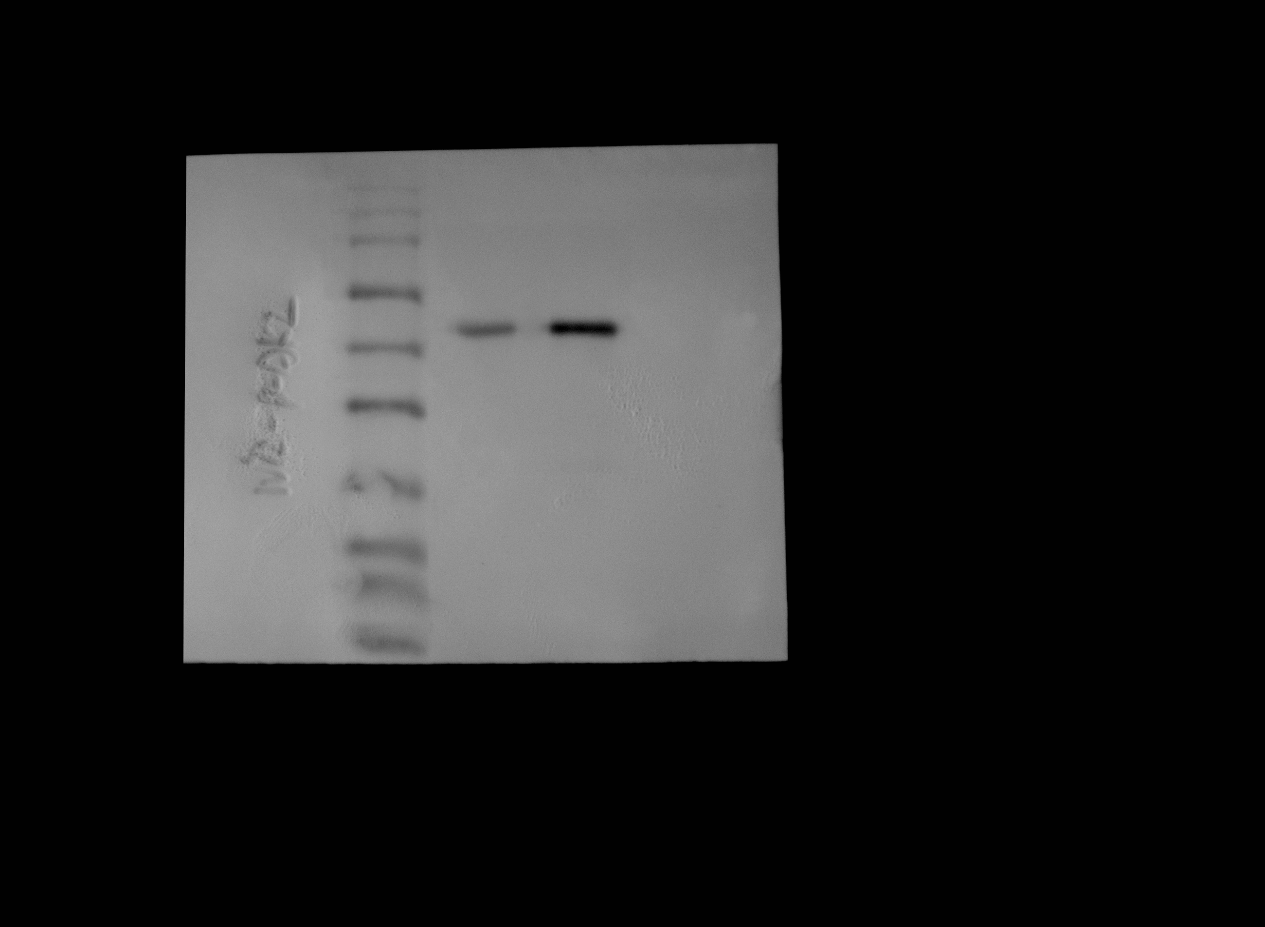
**

**
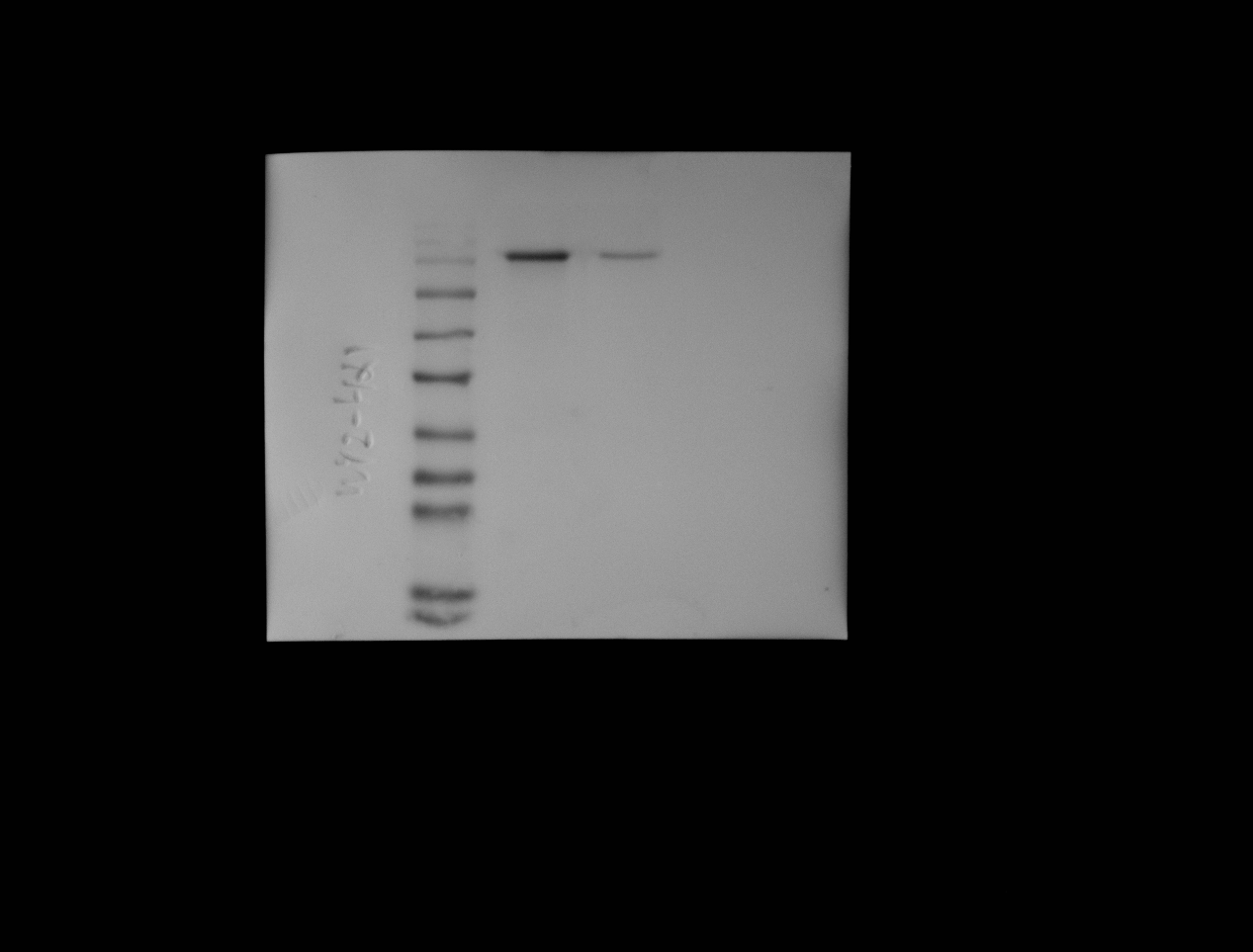
**

**
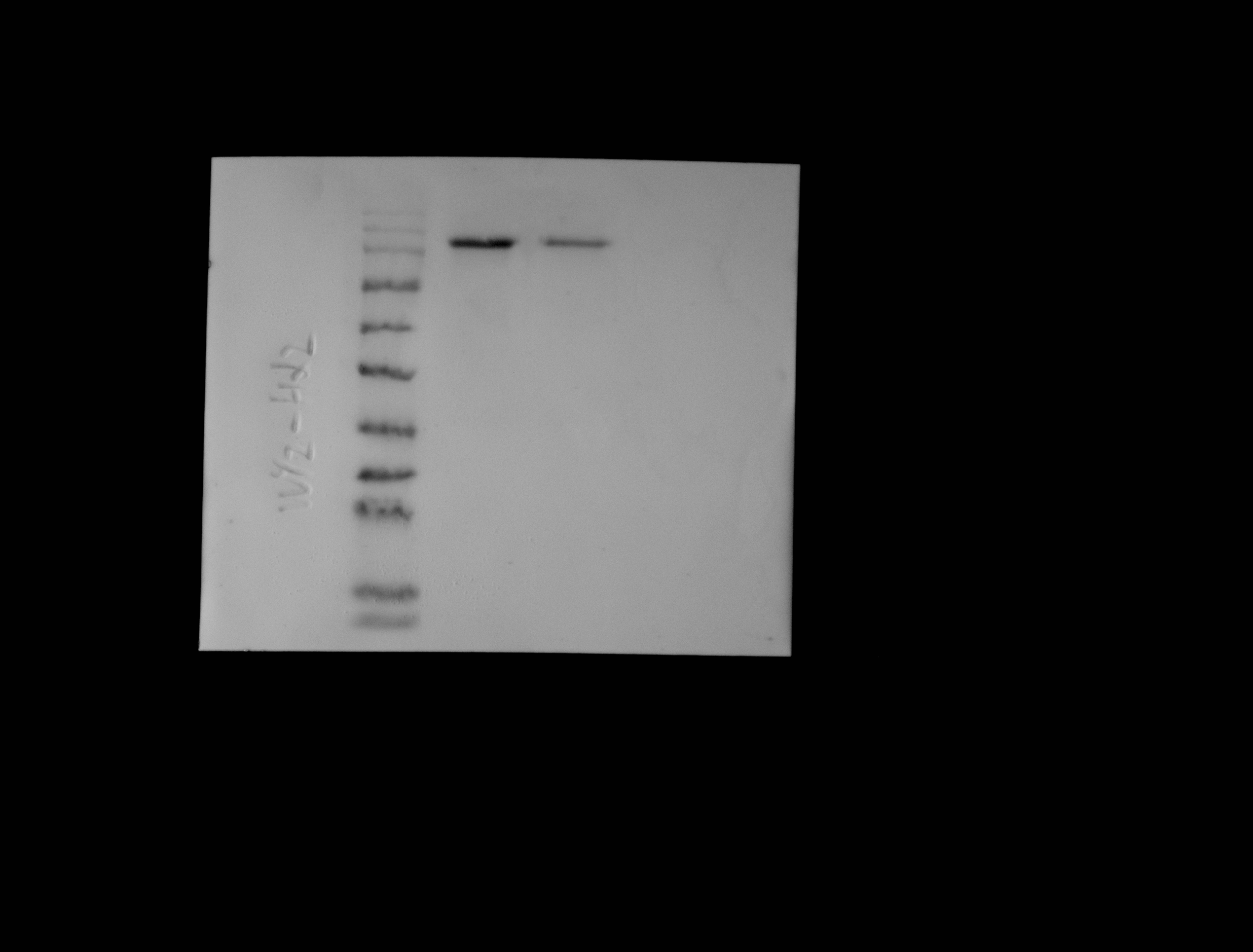
**

**
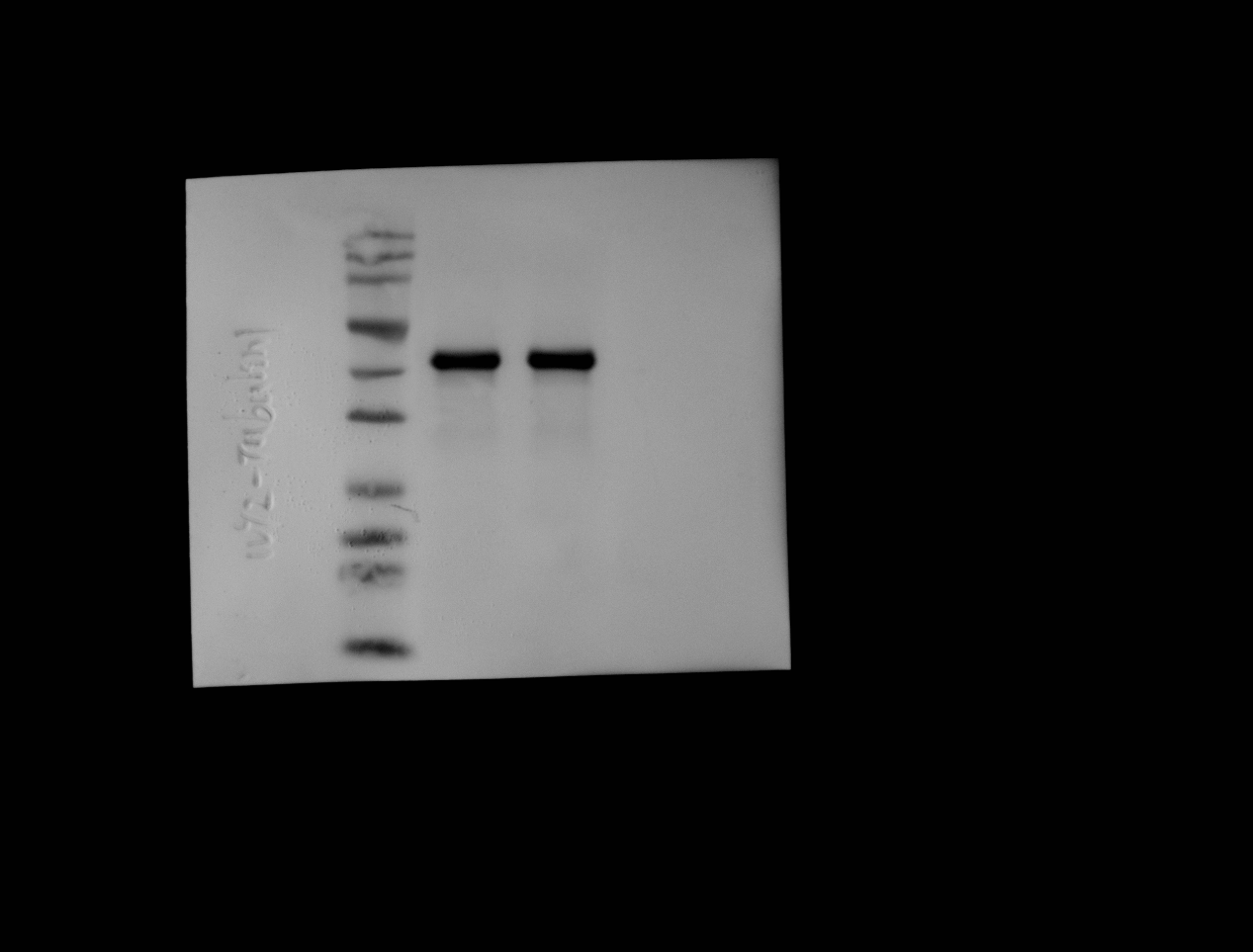
**

**
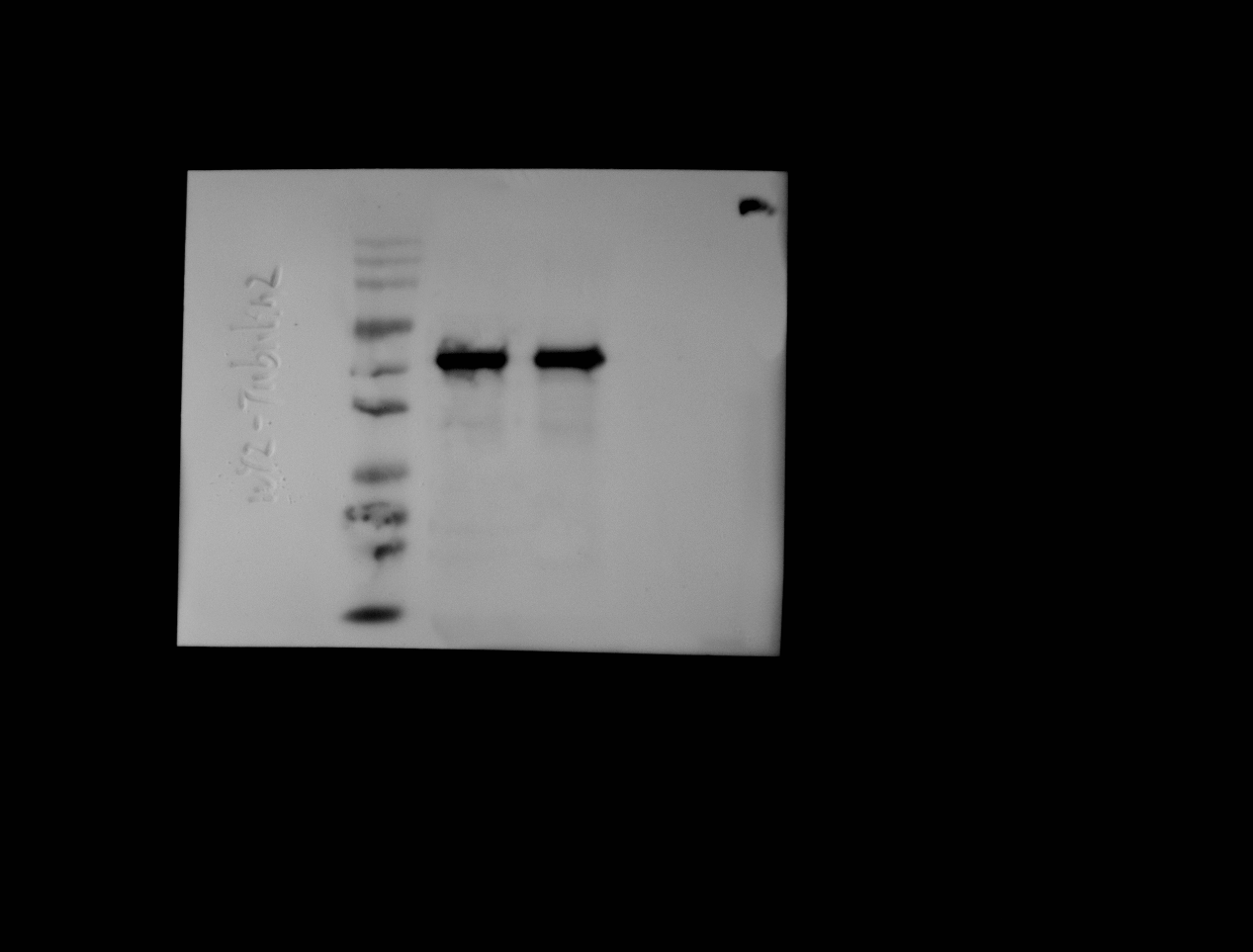
**


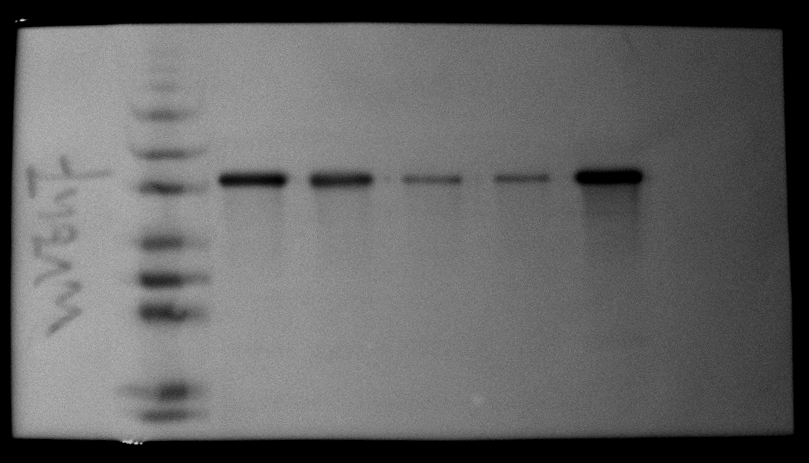


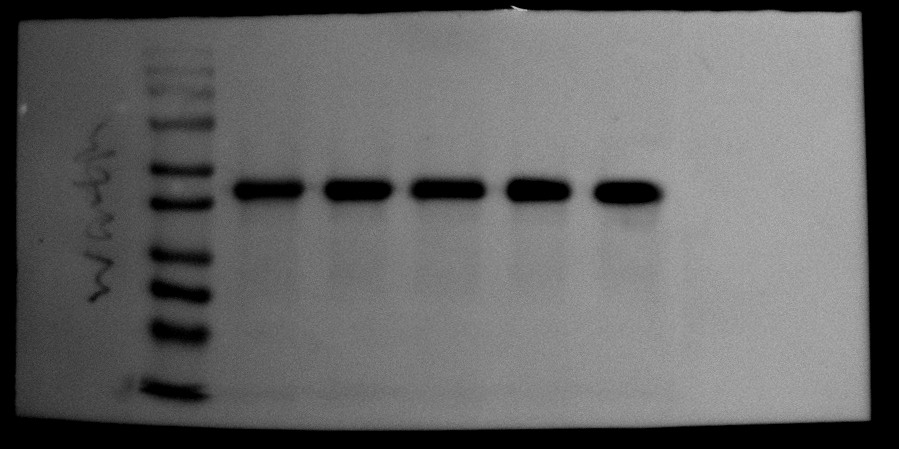


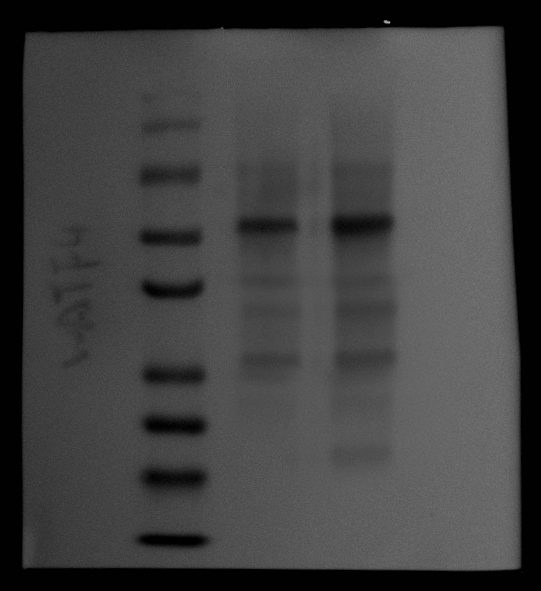


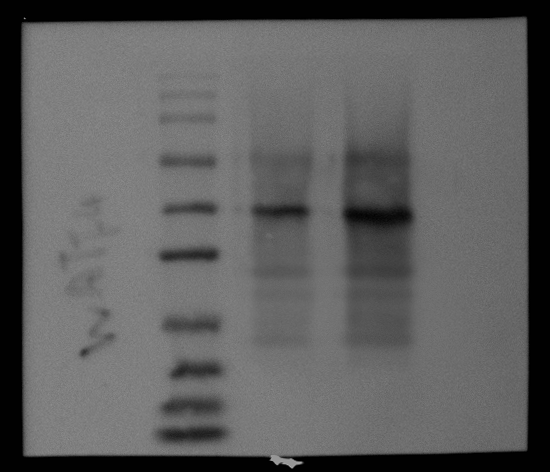


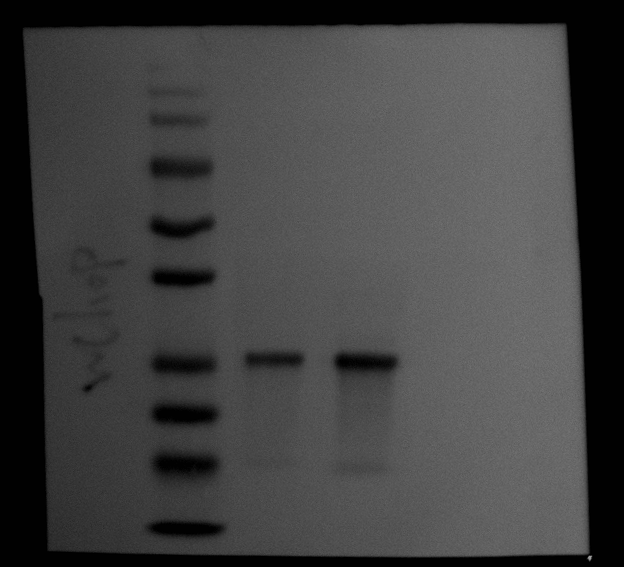


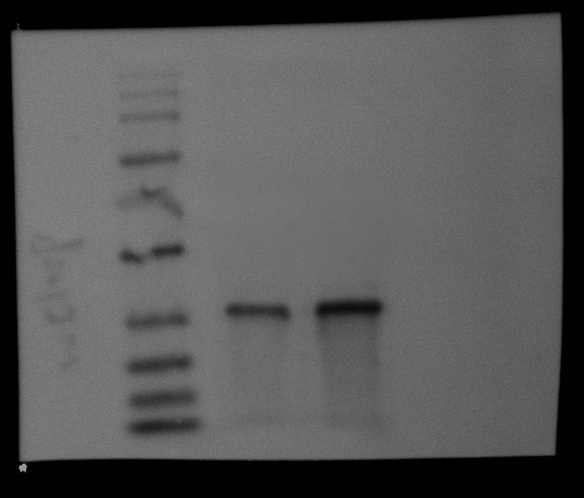


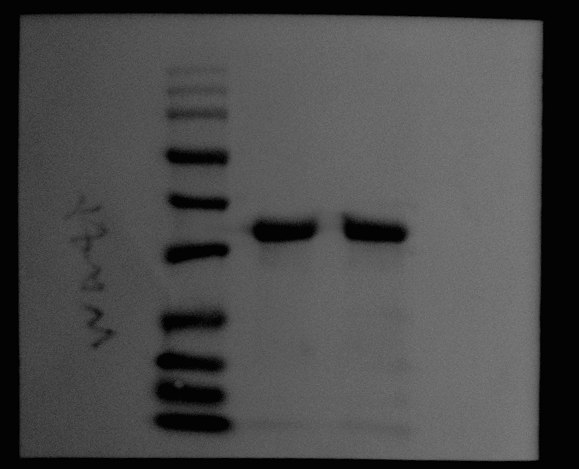


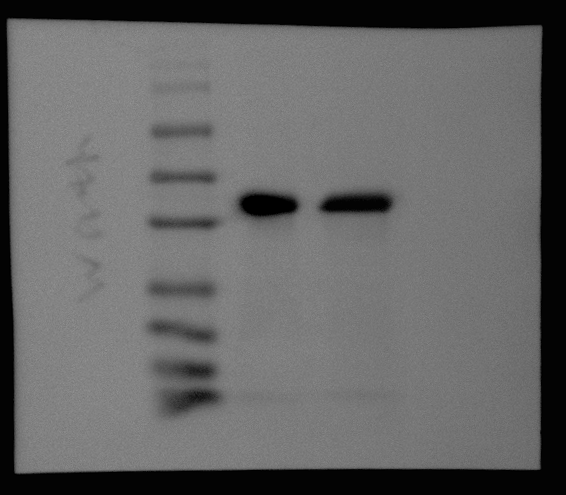


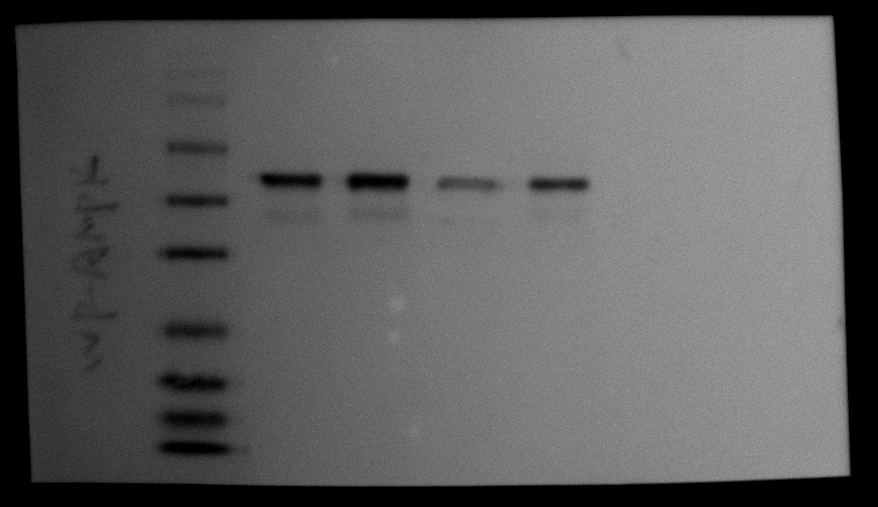


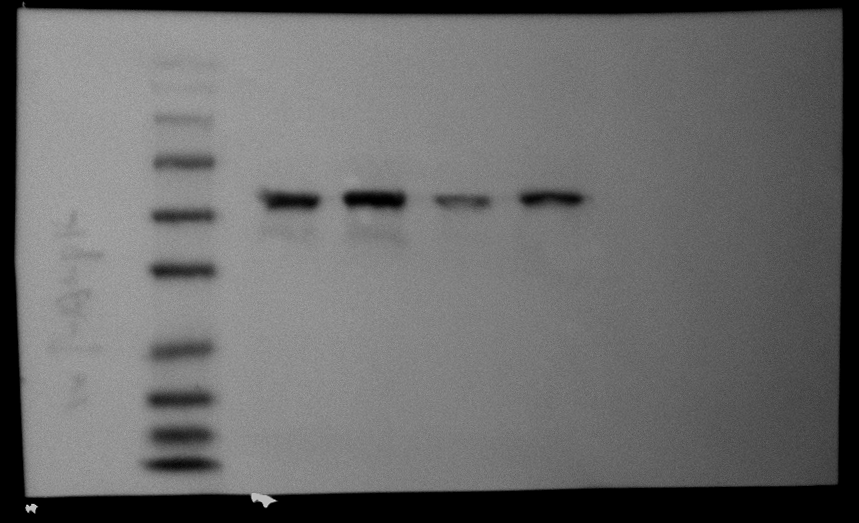


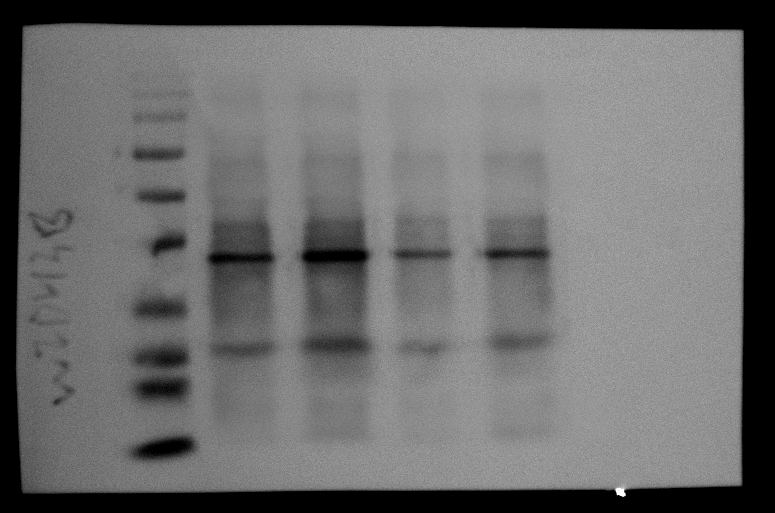


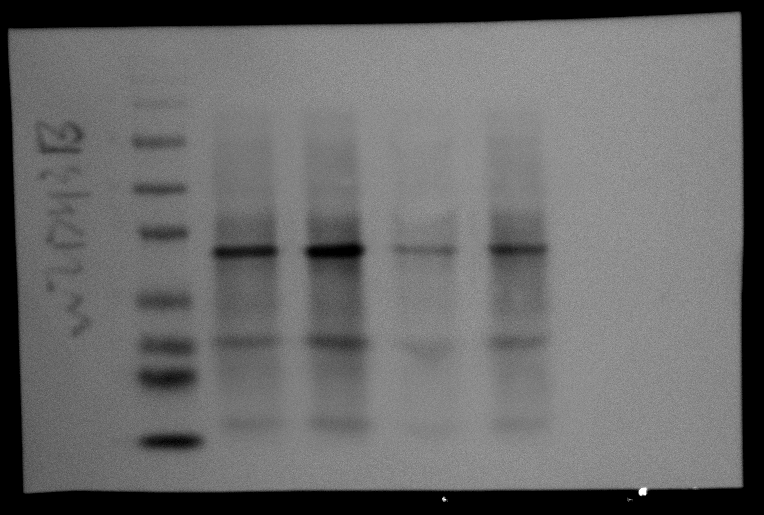


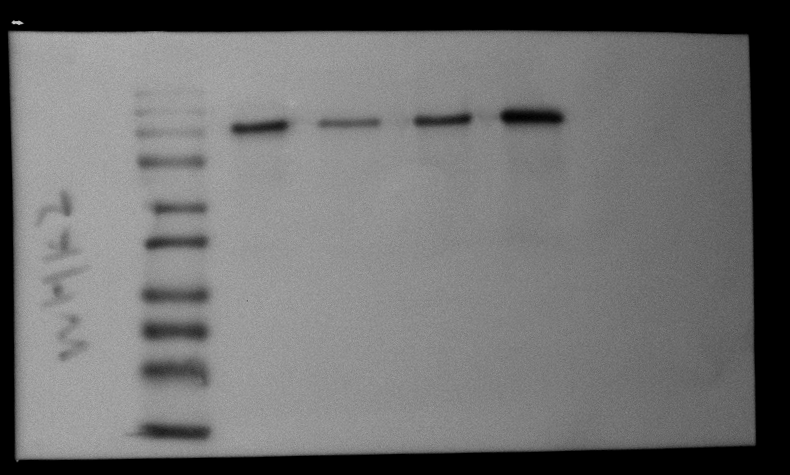


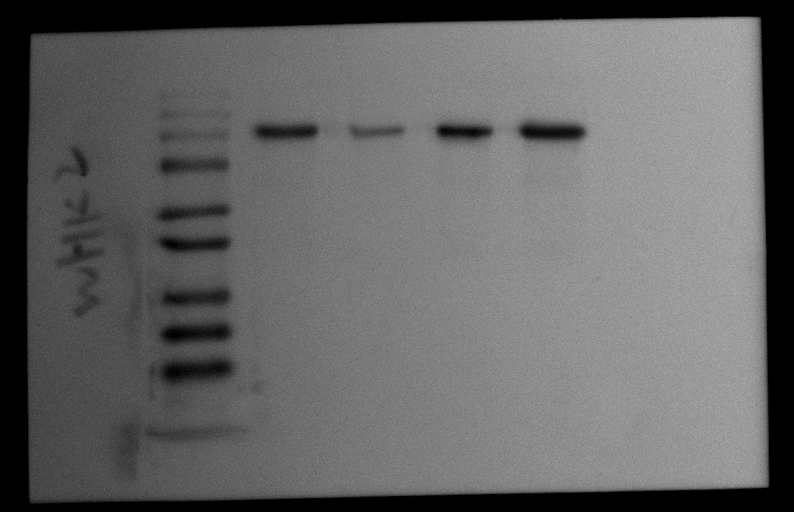


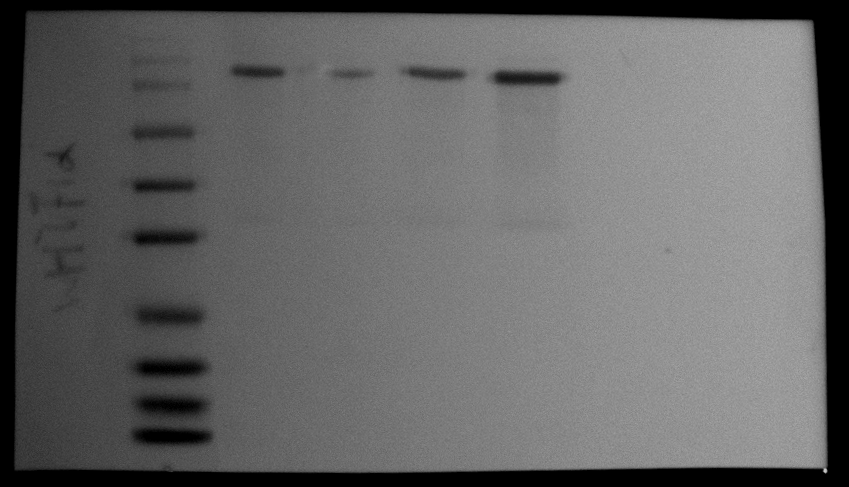


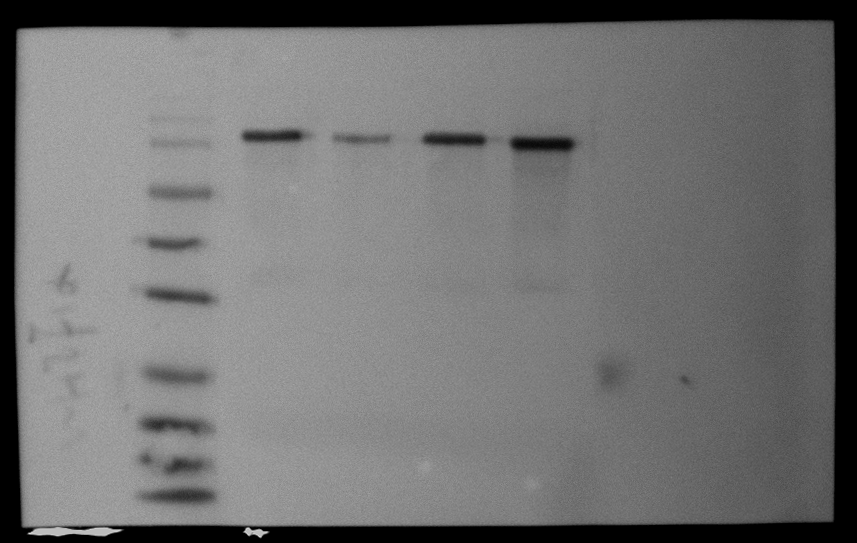


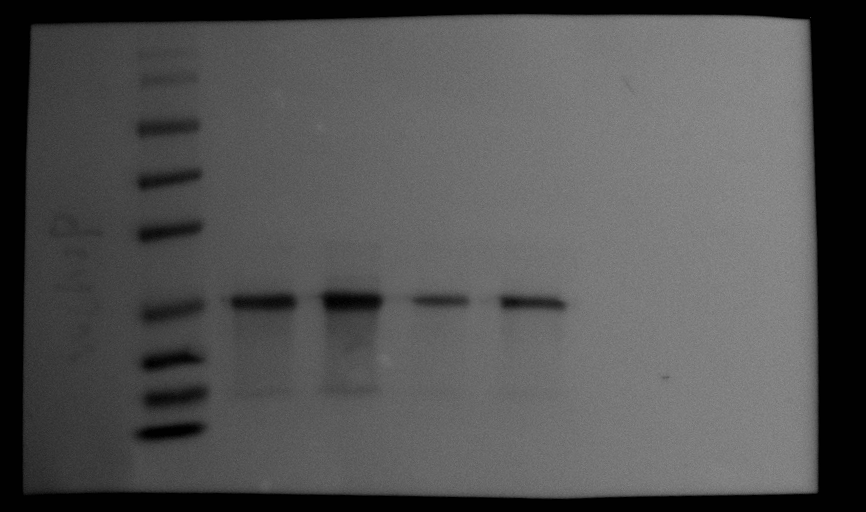


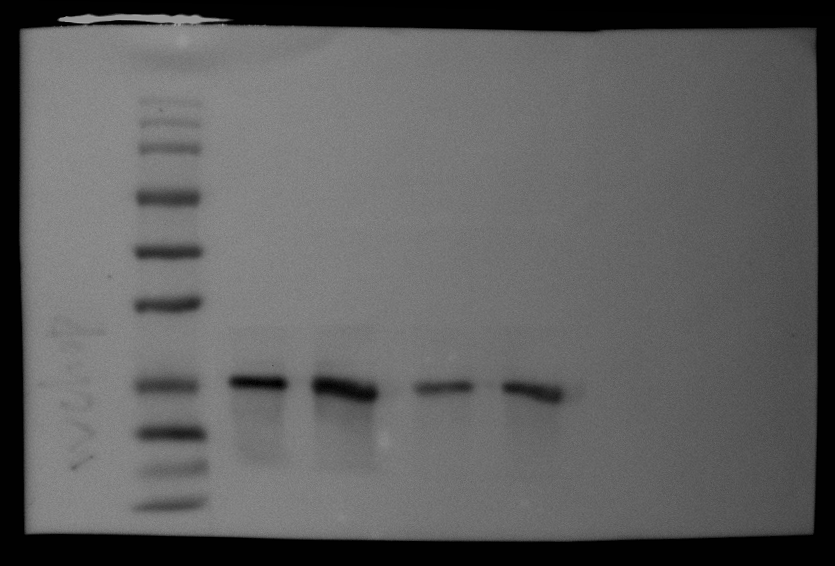


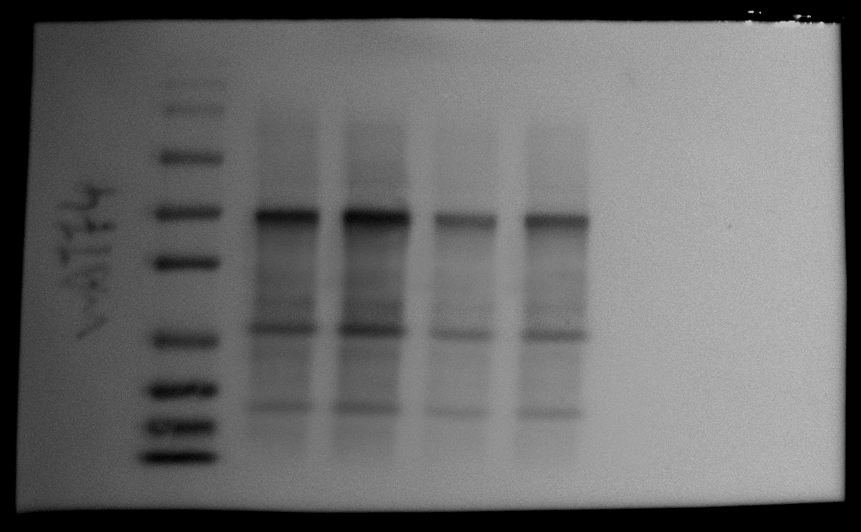


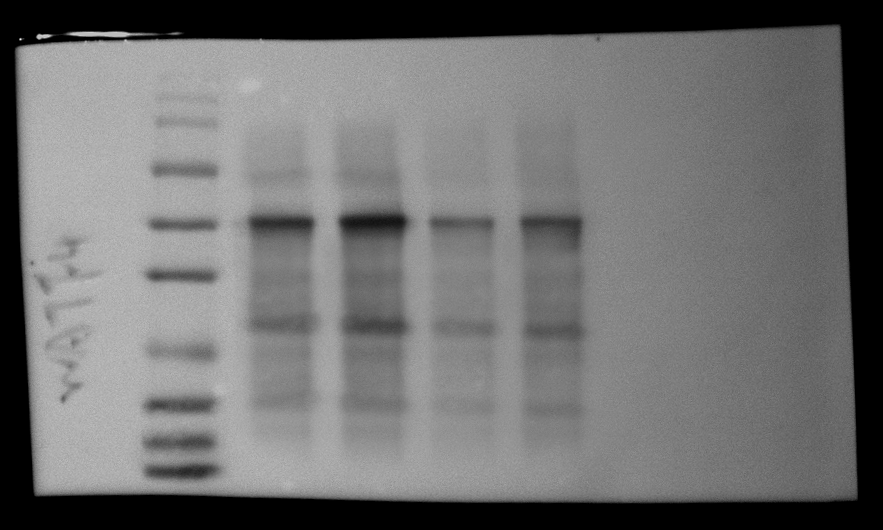


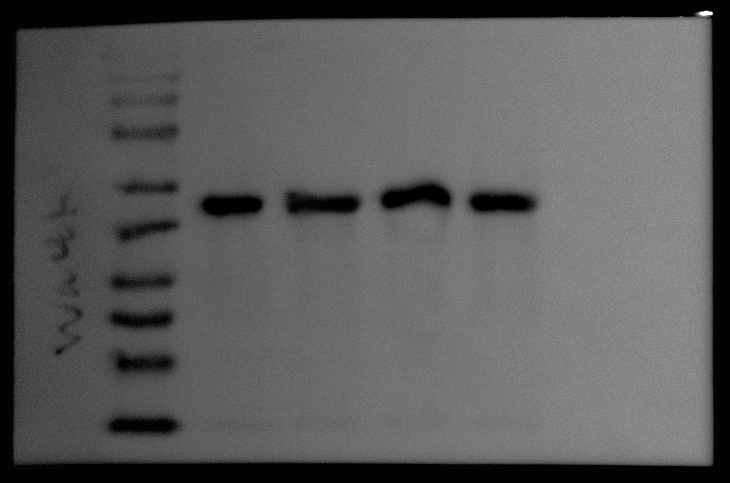


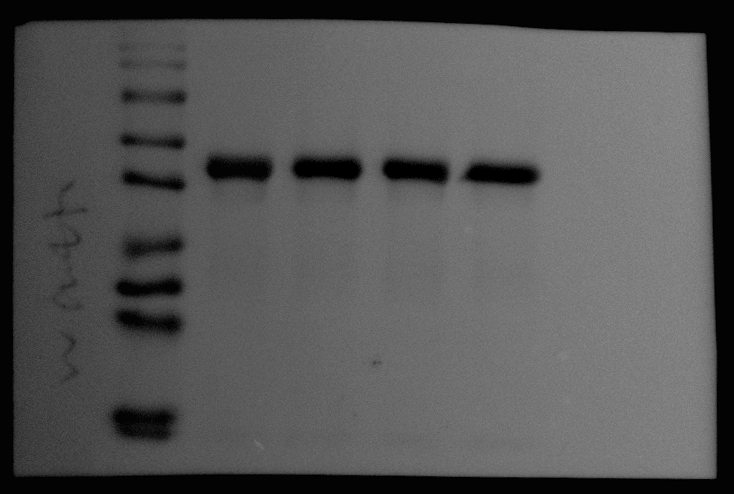


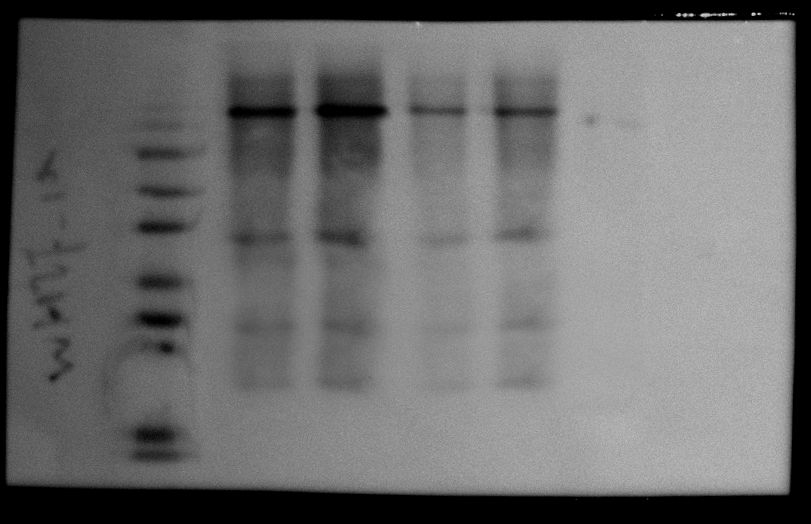


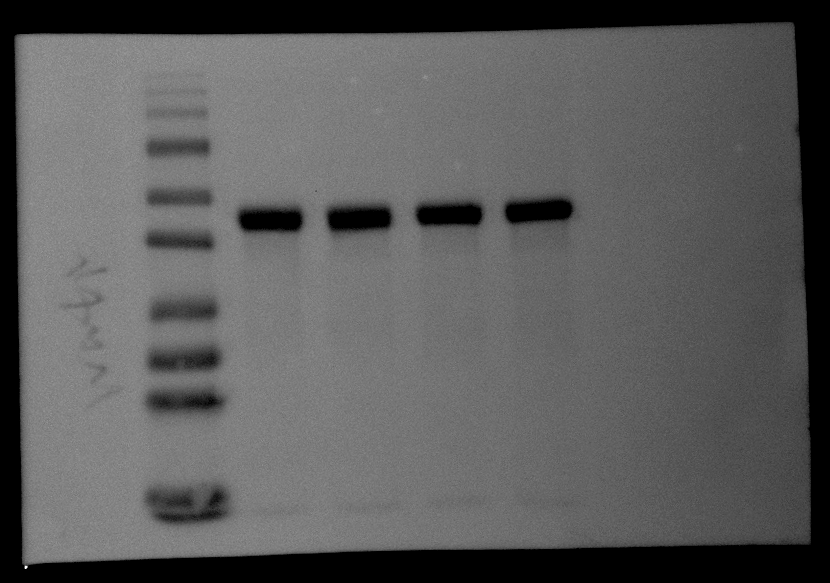


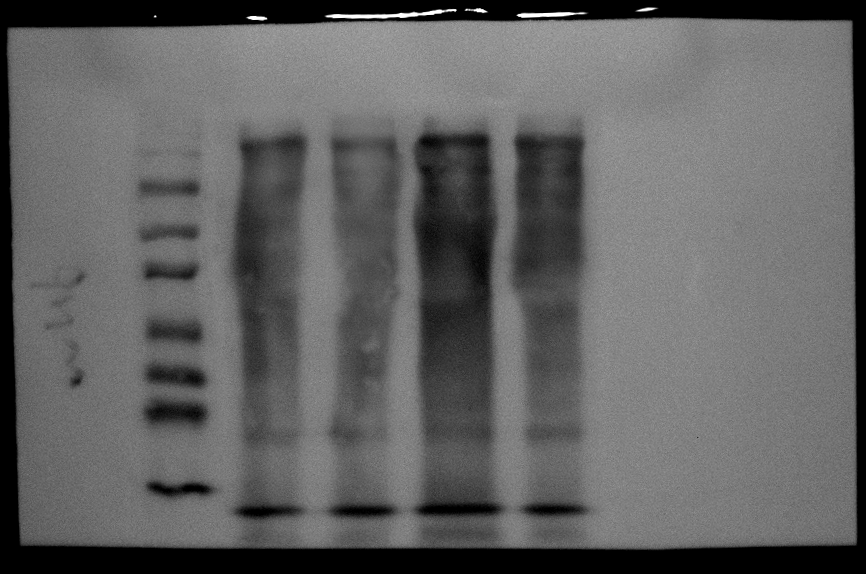


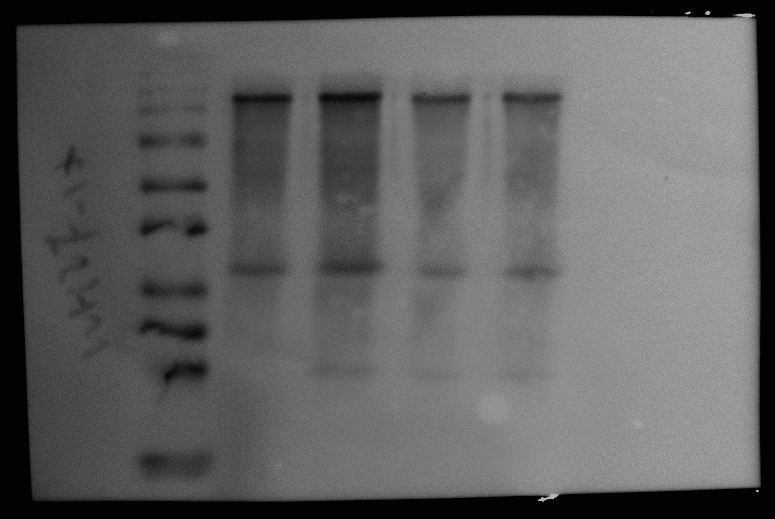


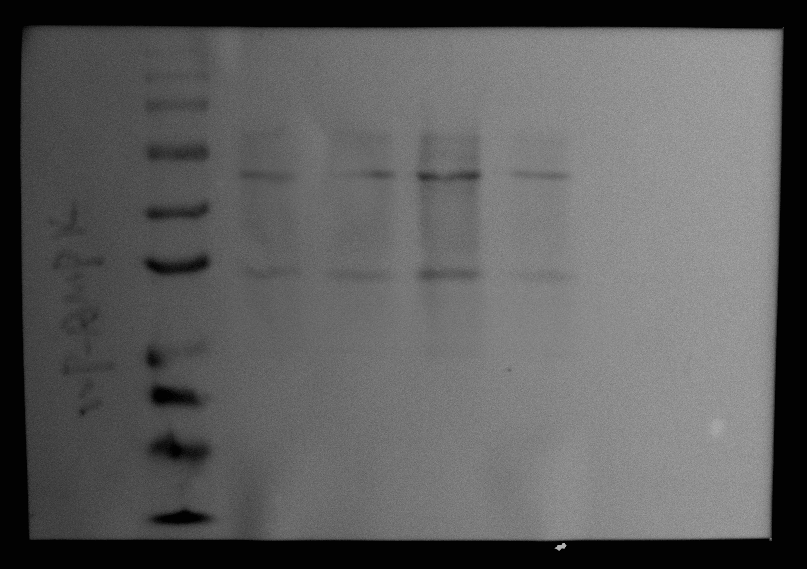


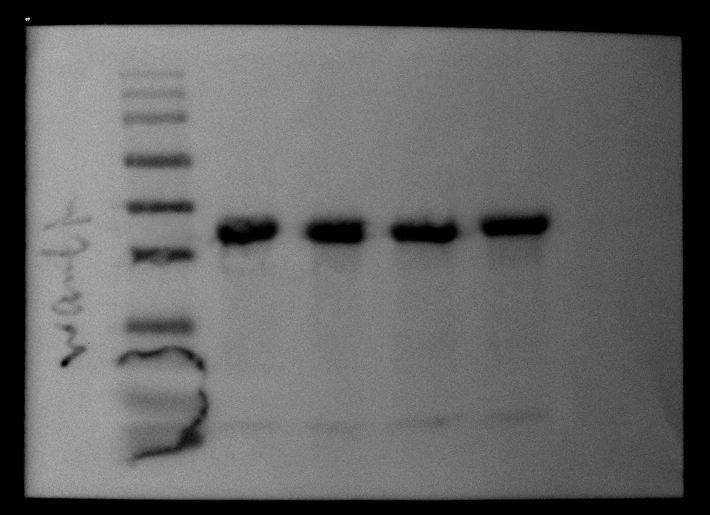


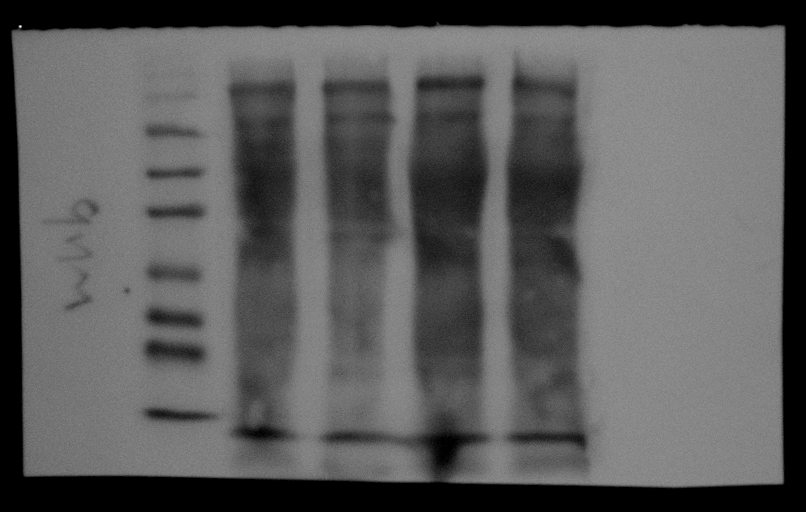


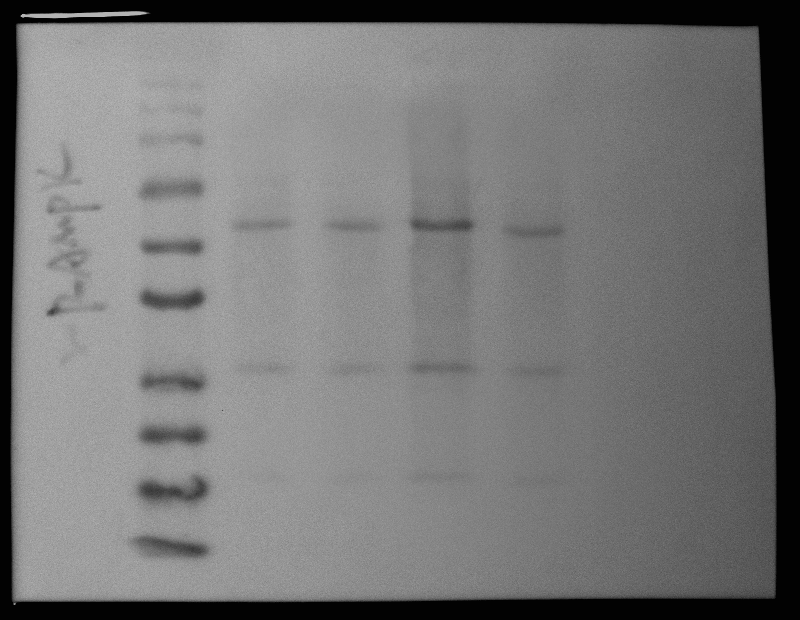


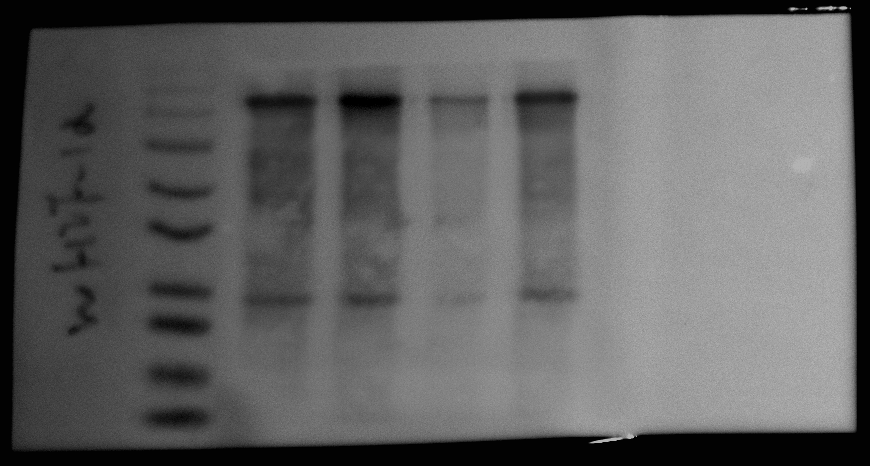


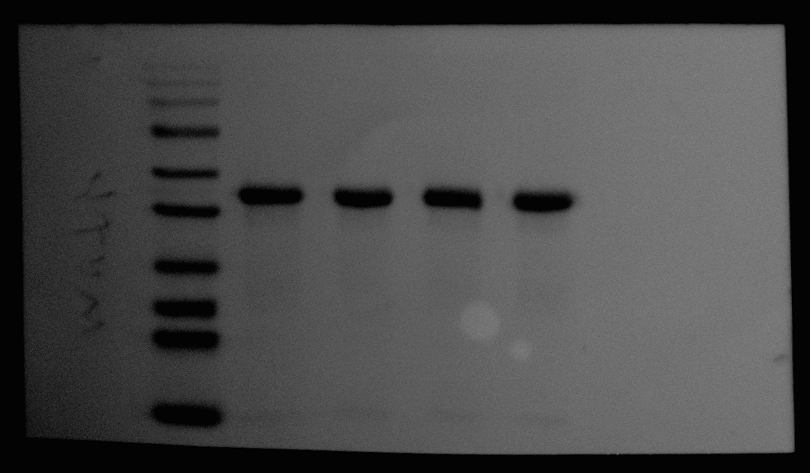


**
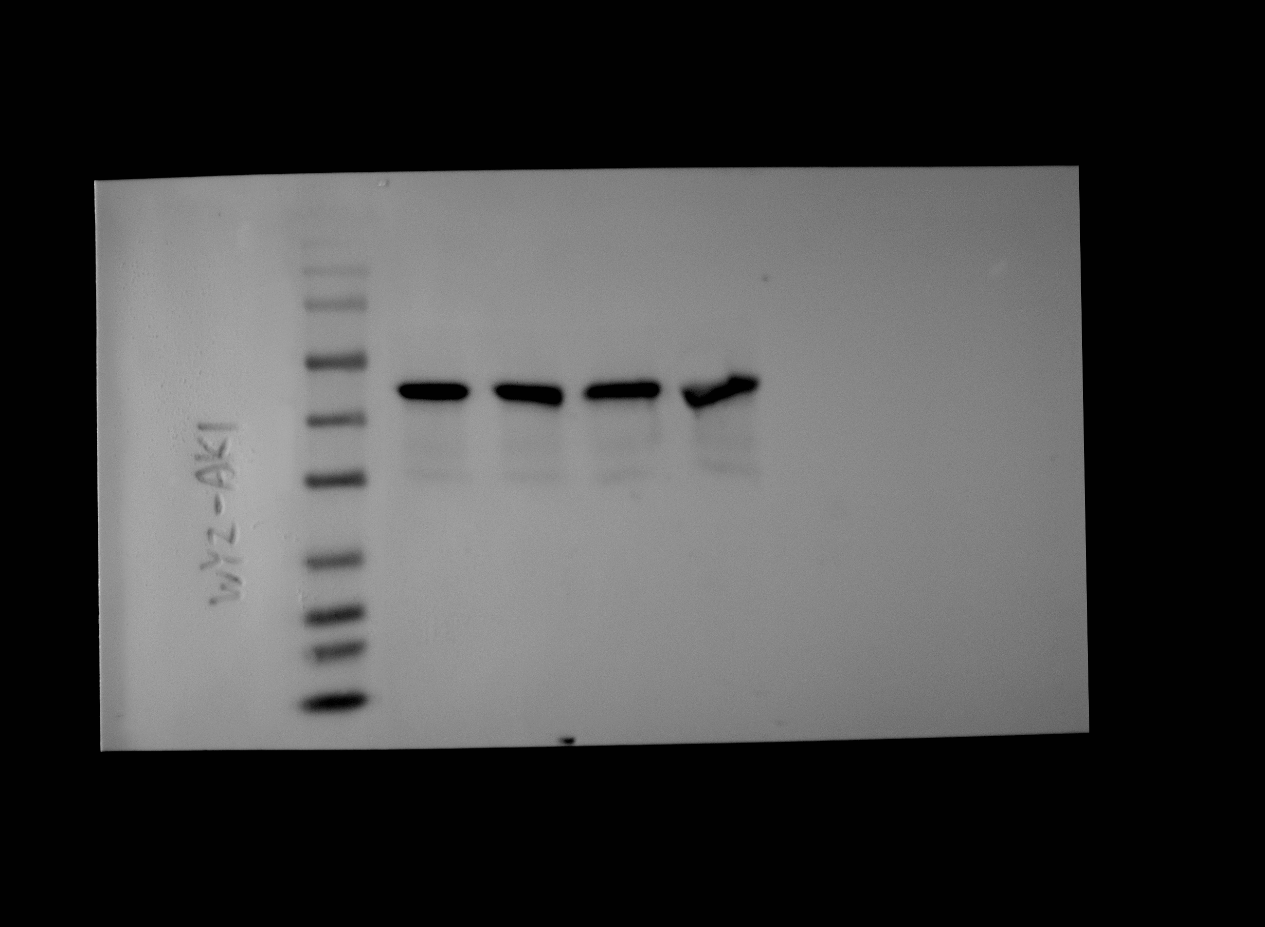
**

**
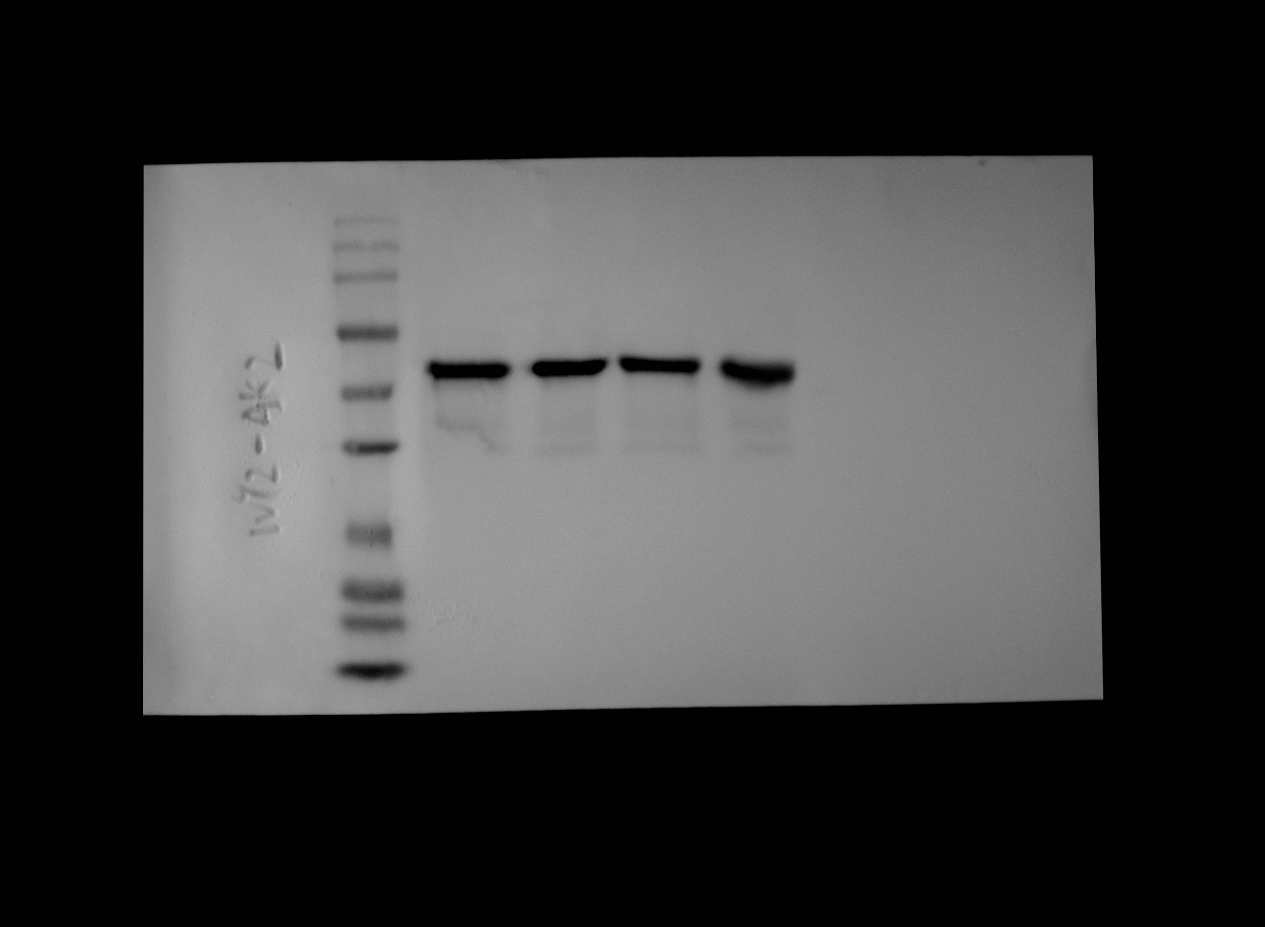

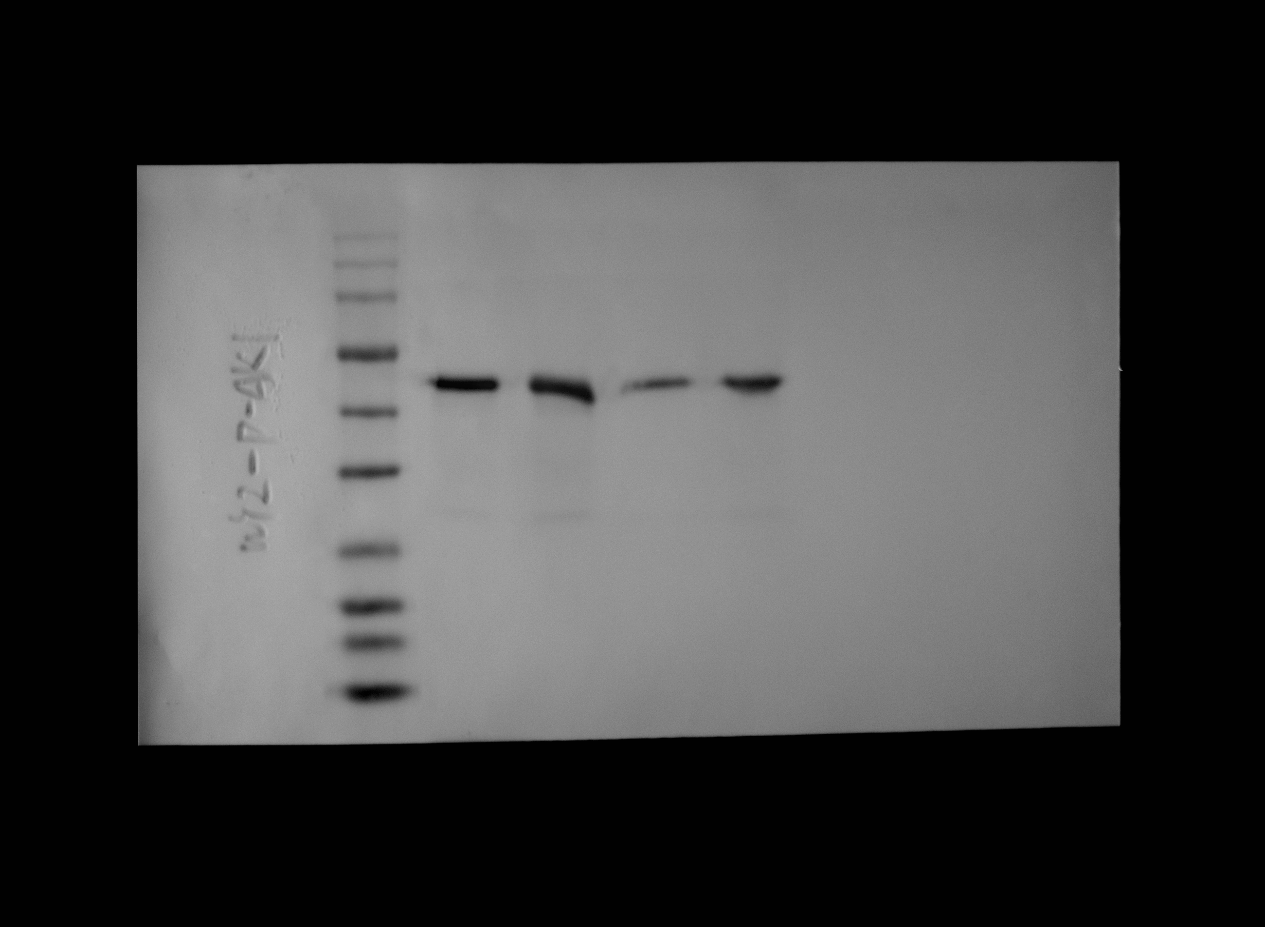
**

**
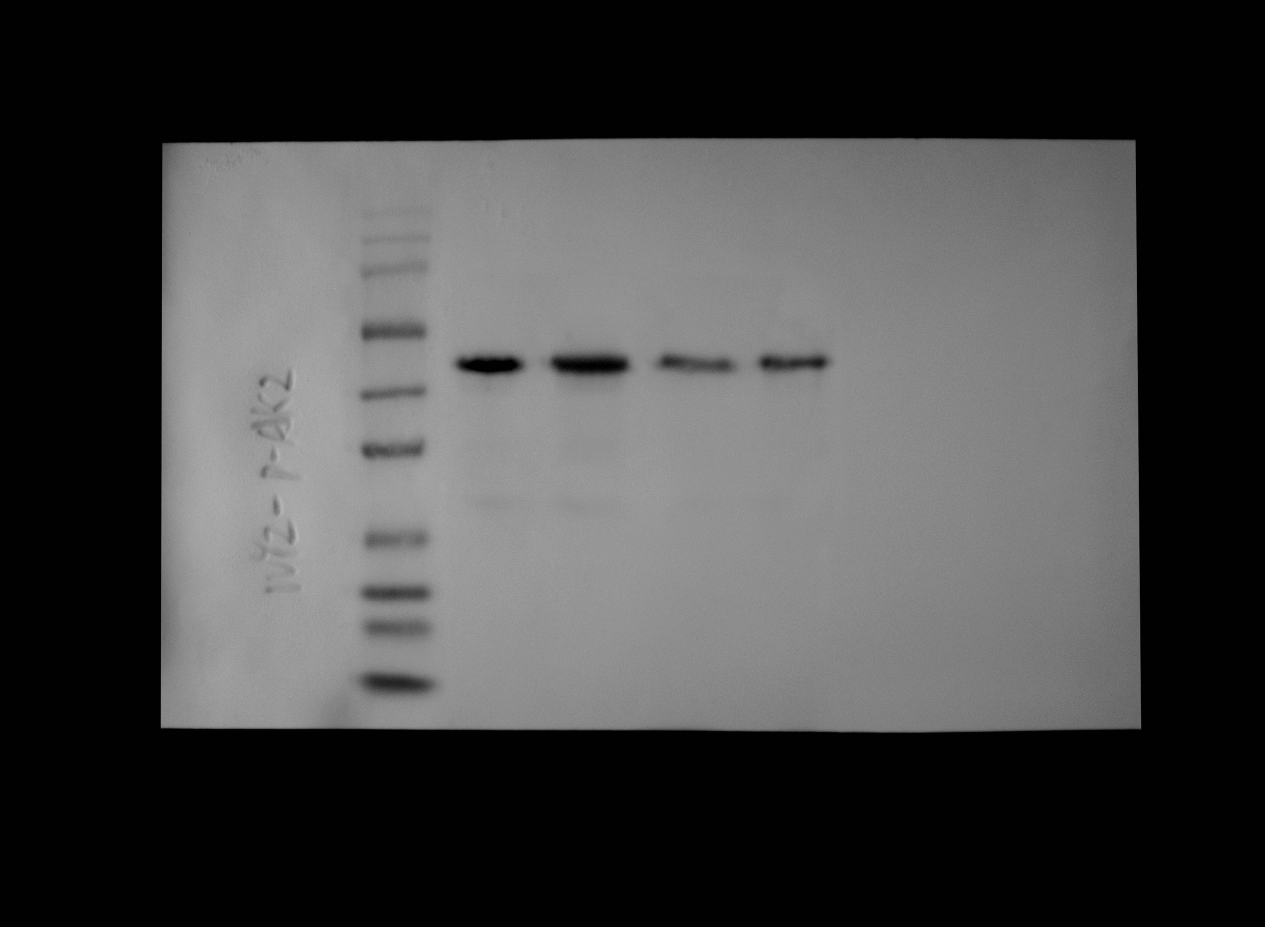
**

**
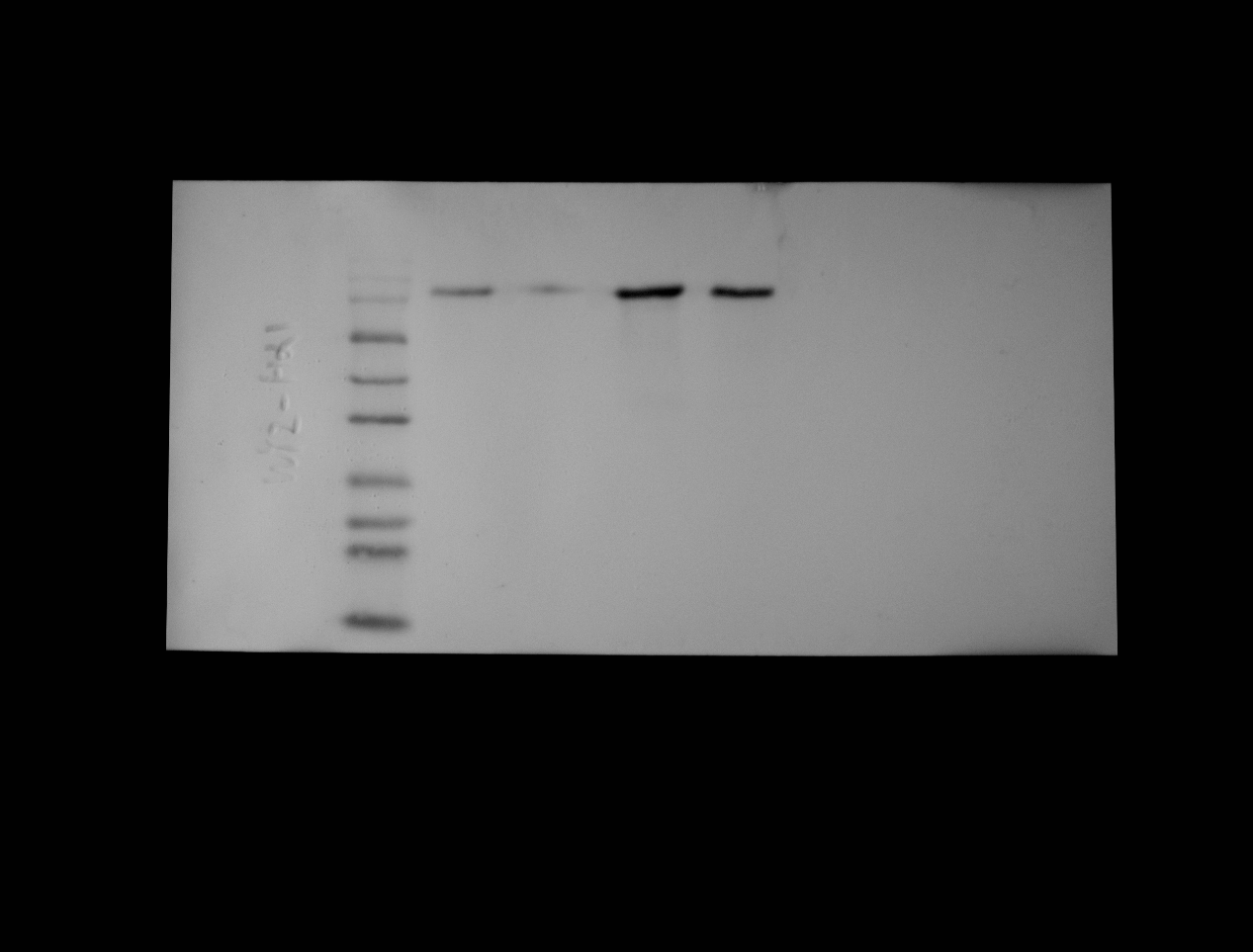
**

**
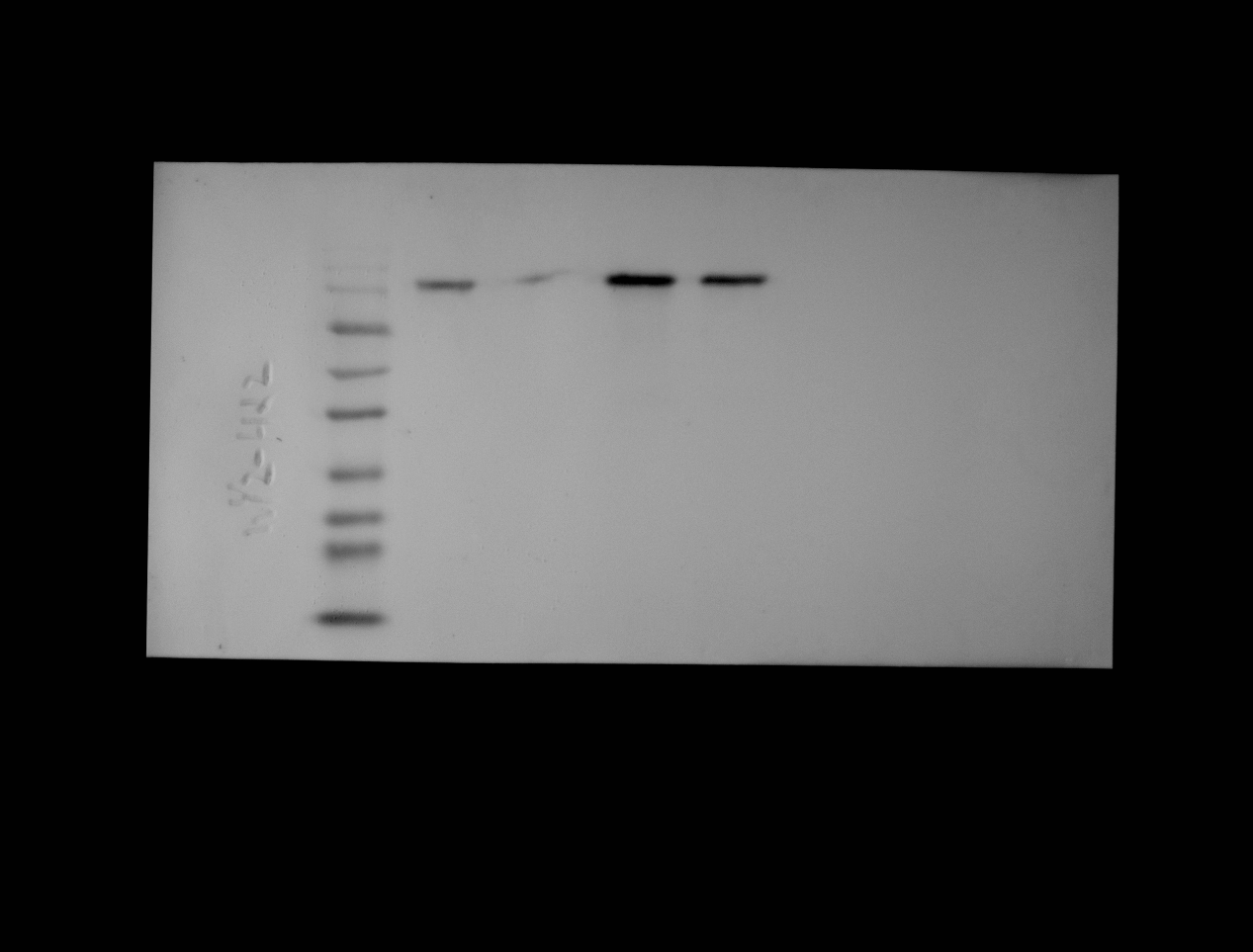
**

**
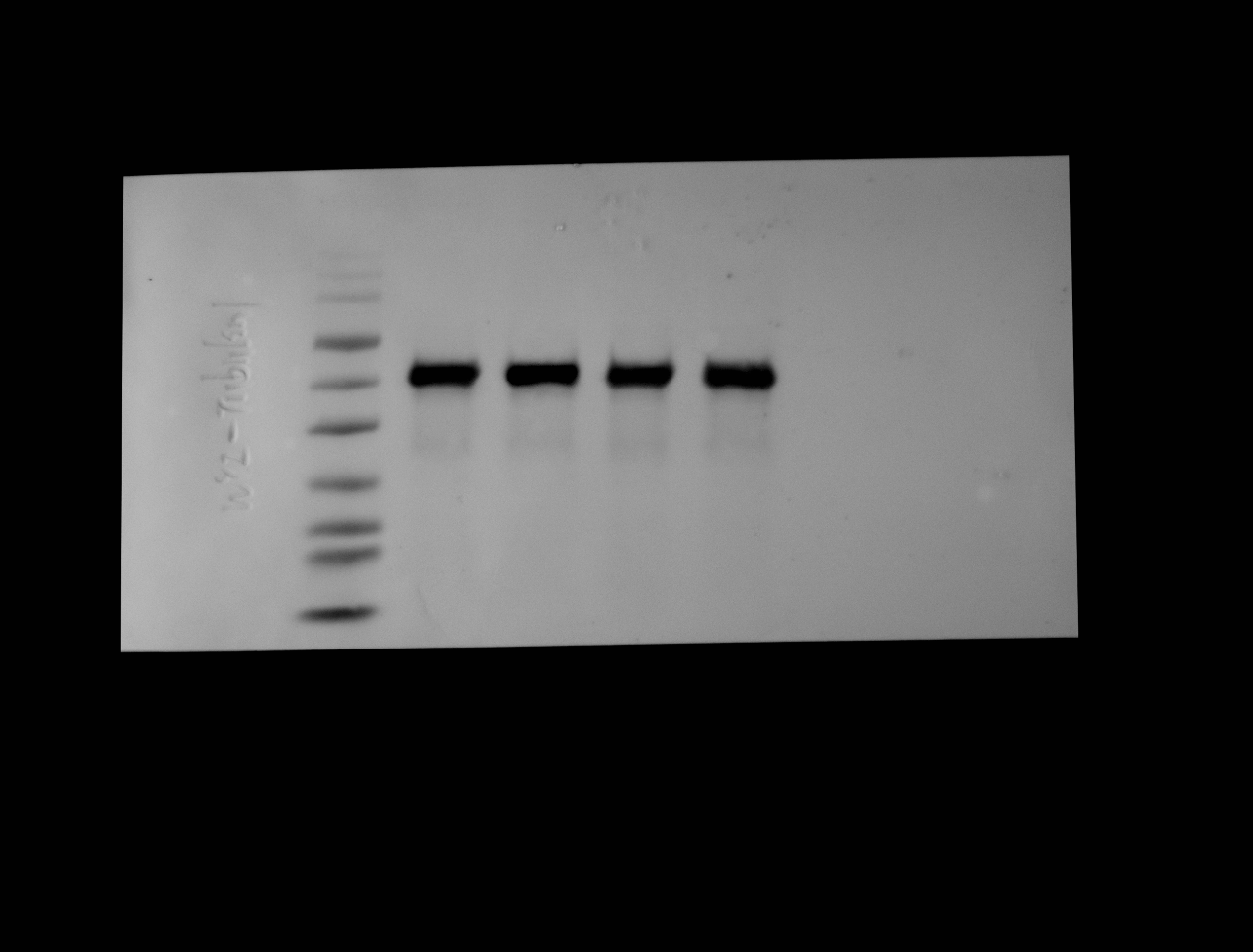
**

**
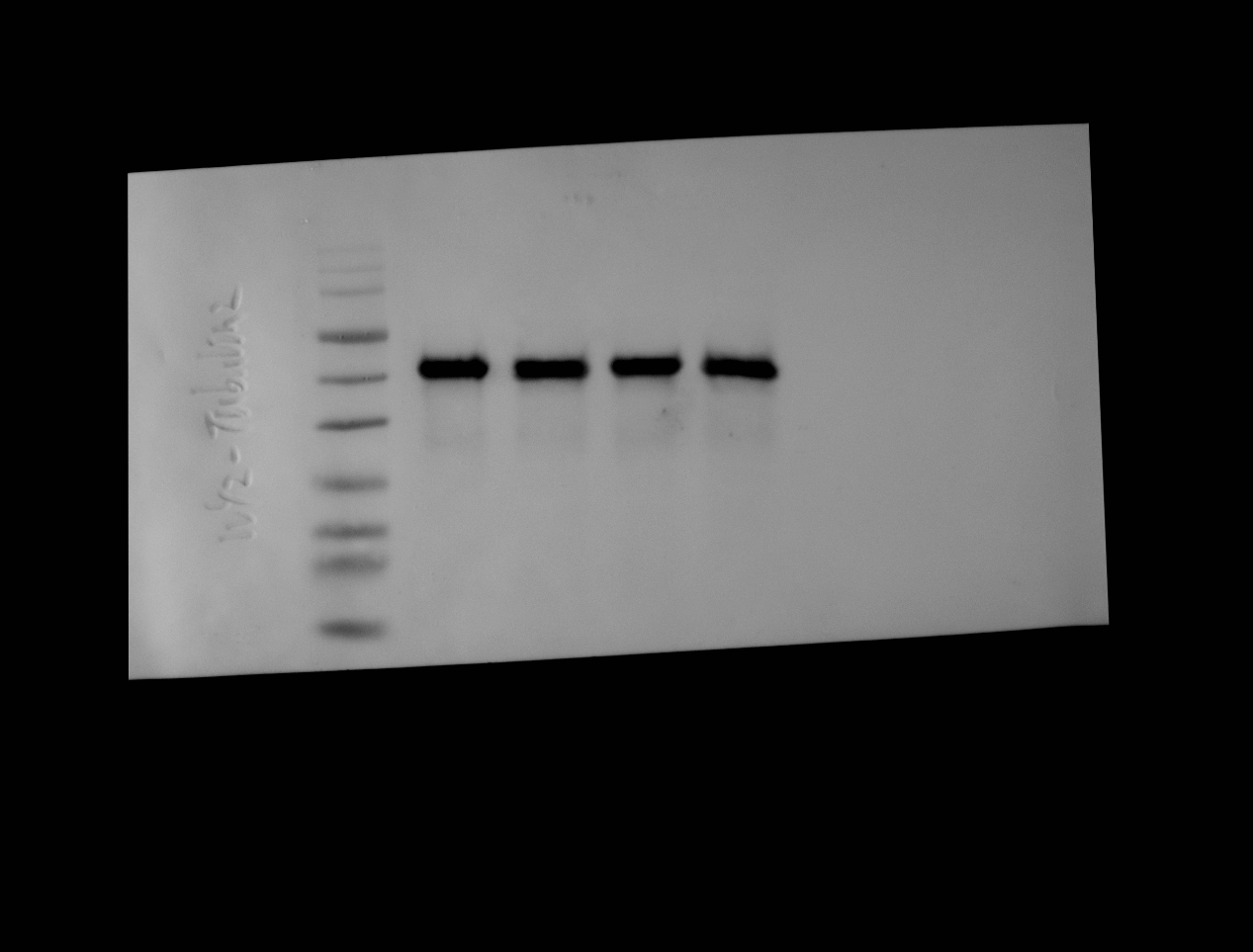
**

**
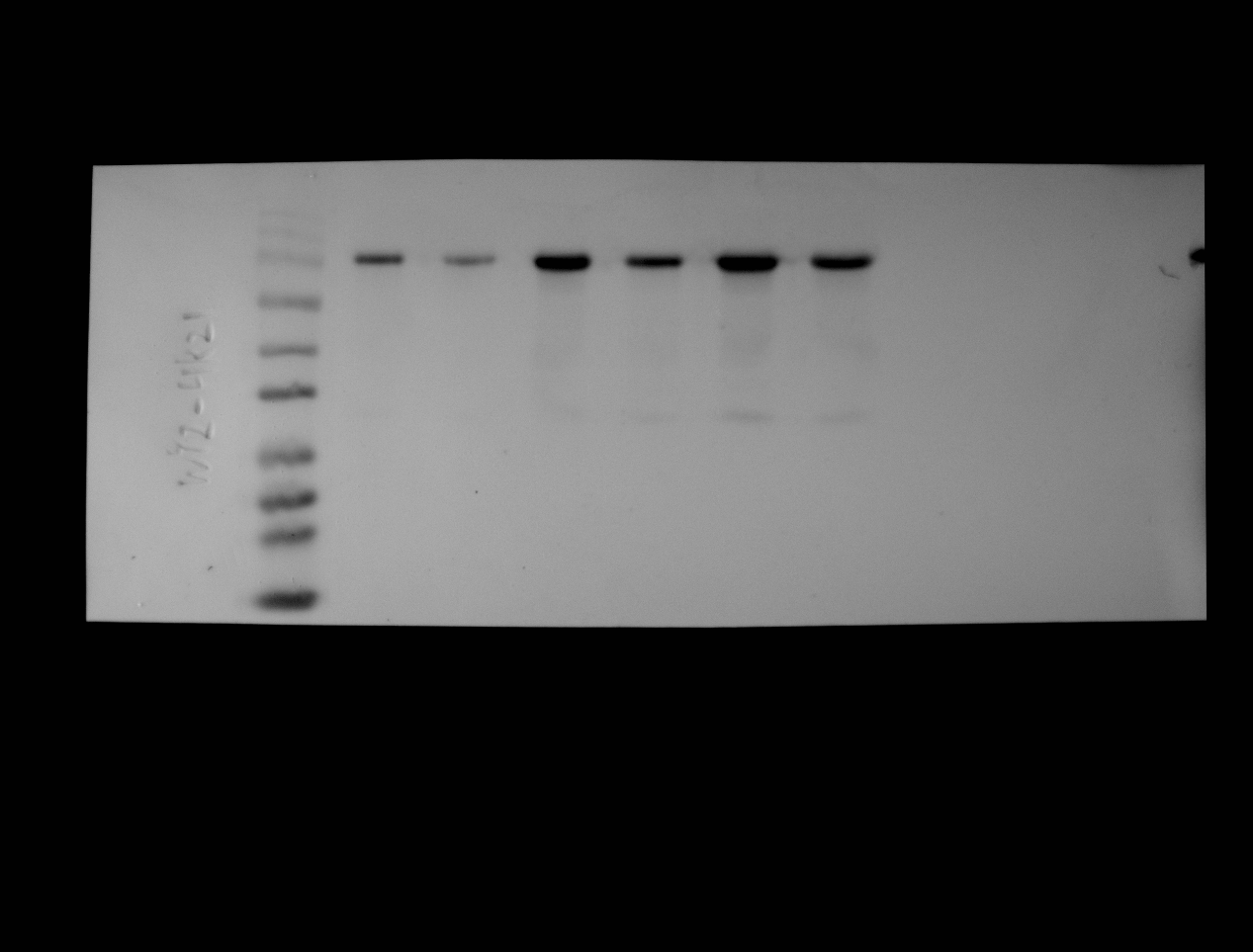
**

**
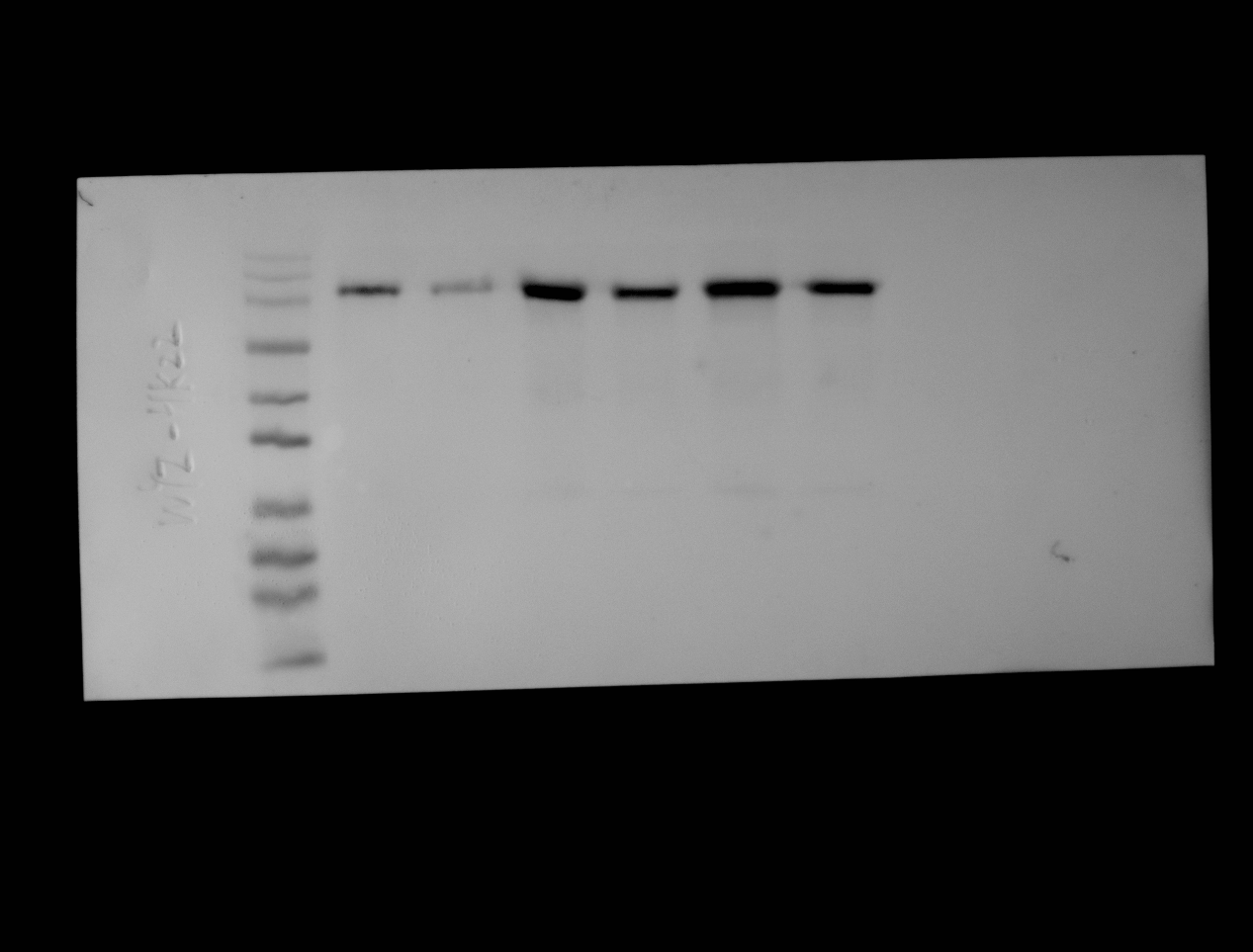
**

**
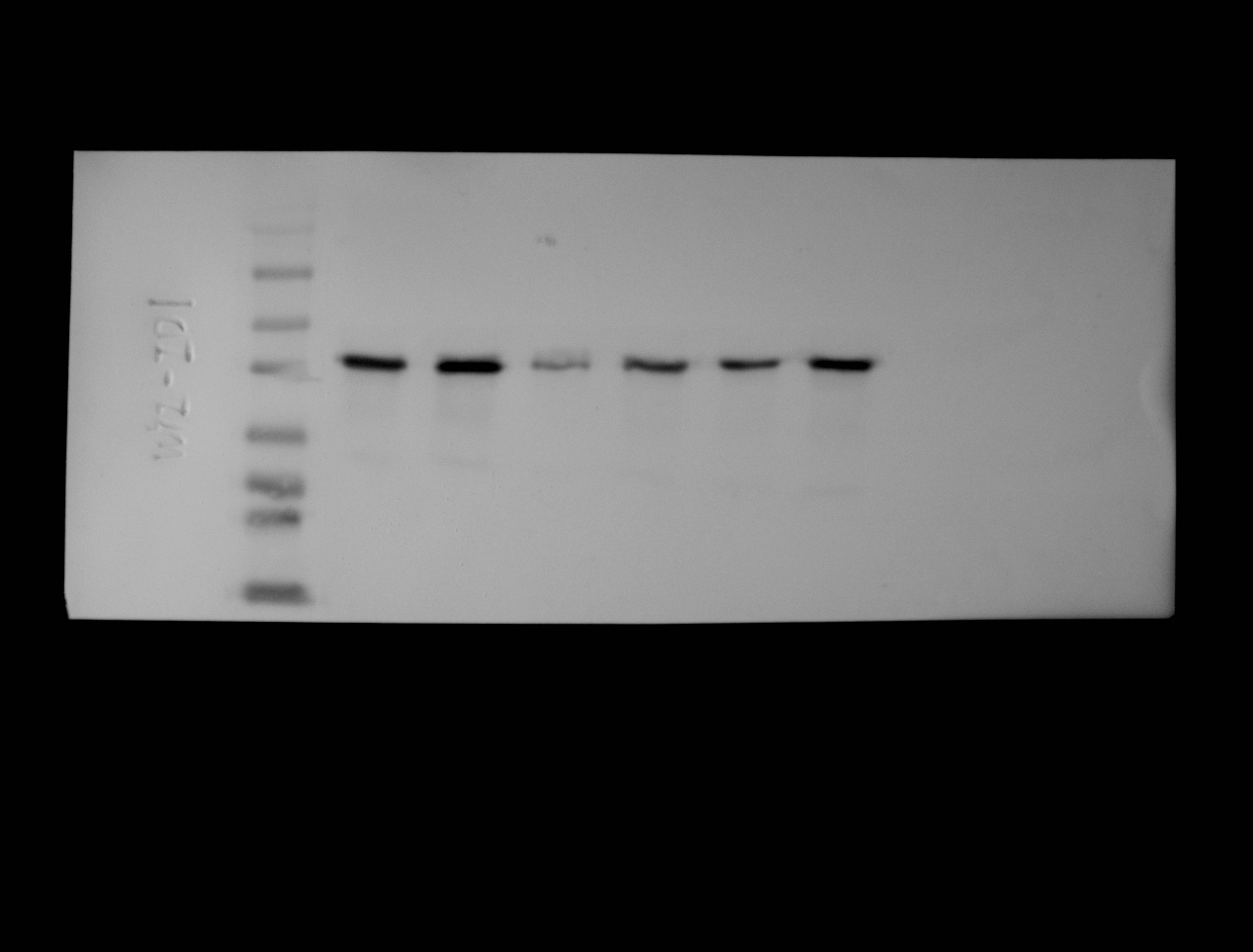
**

**
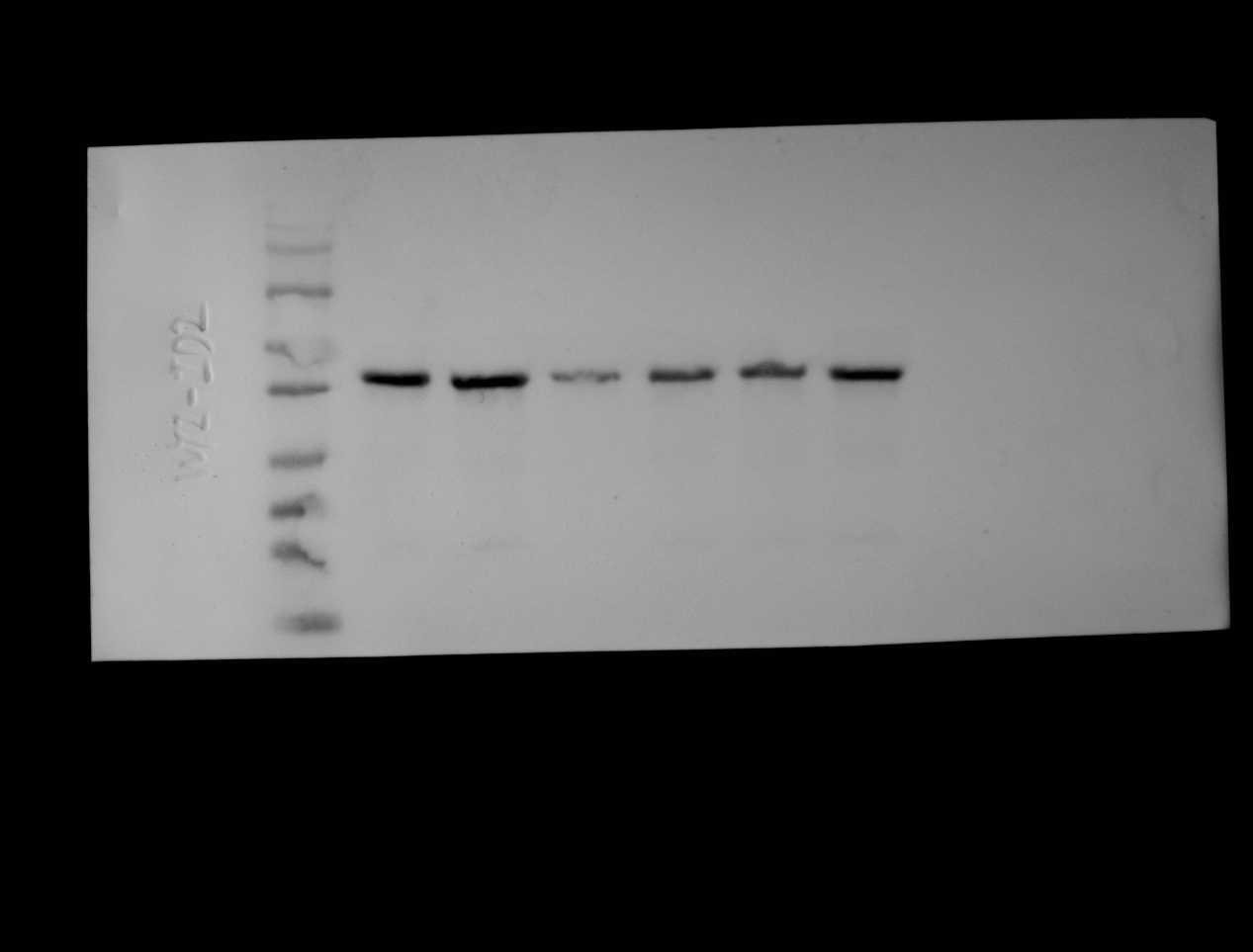
**

**
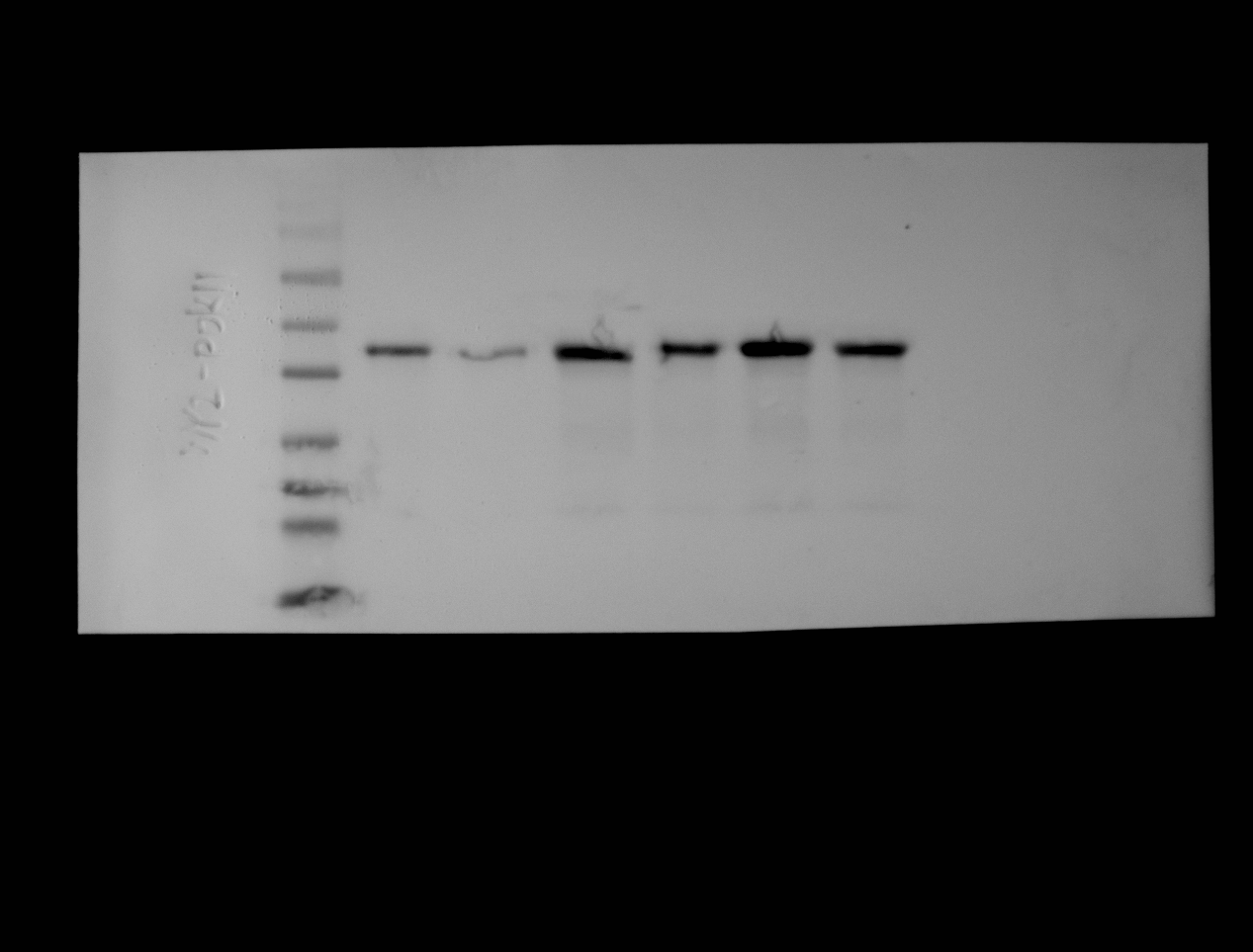
**

**
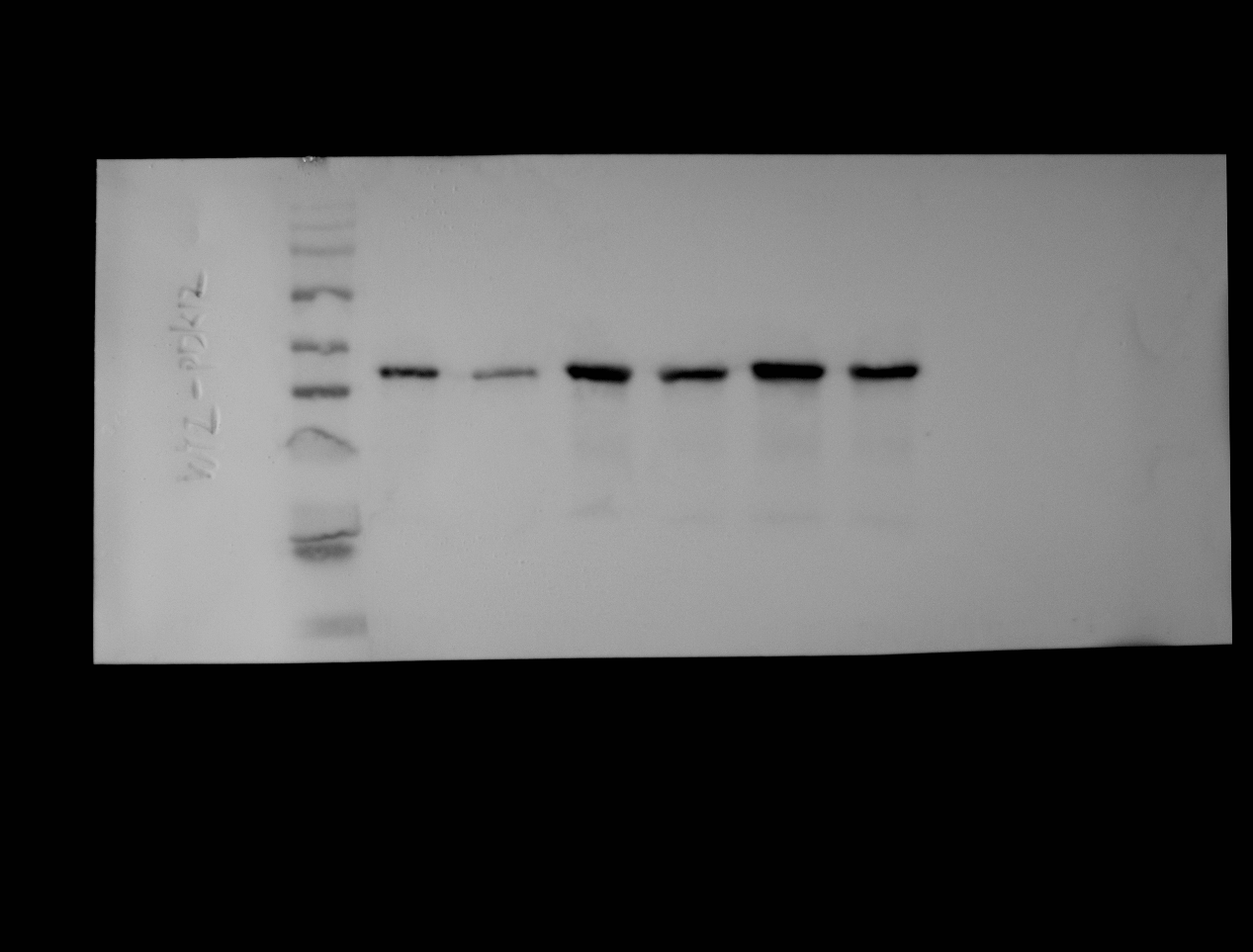
**

**
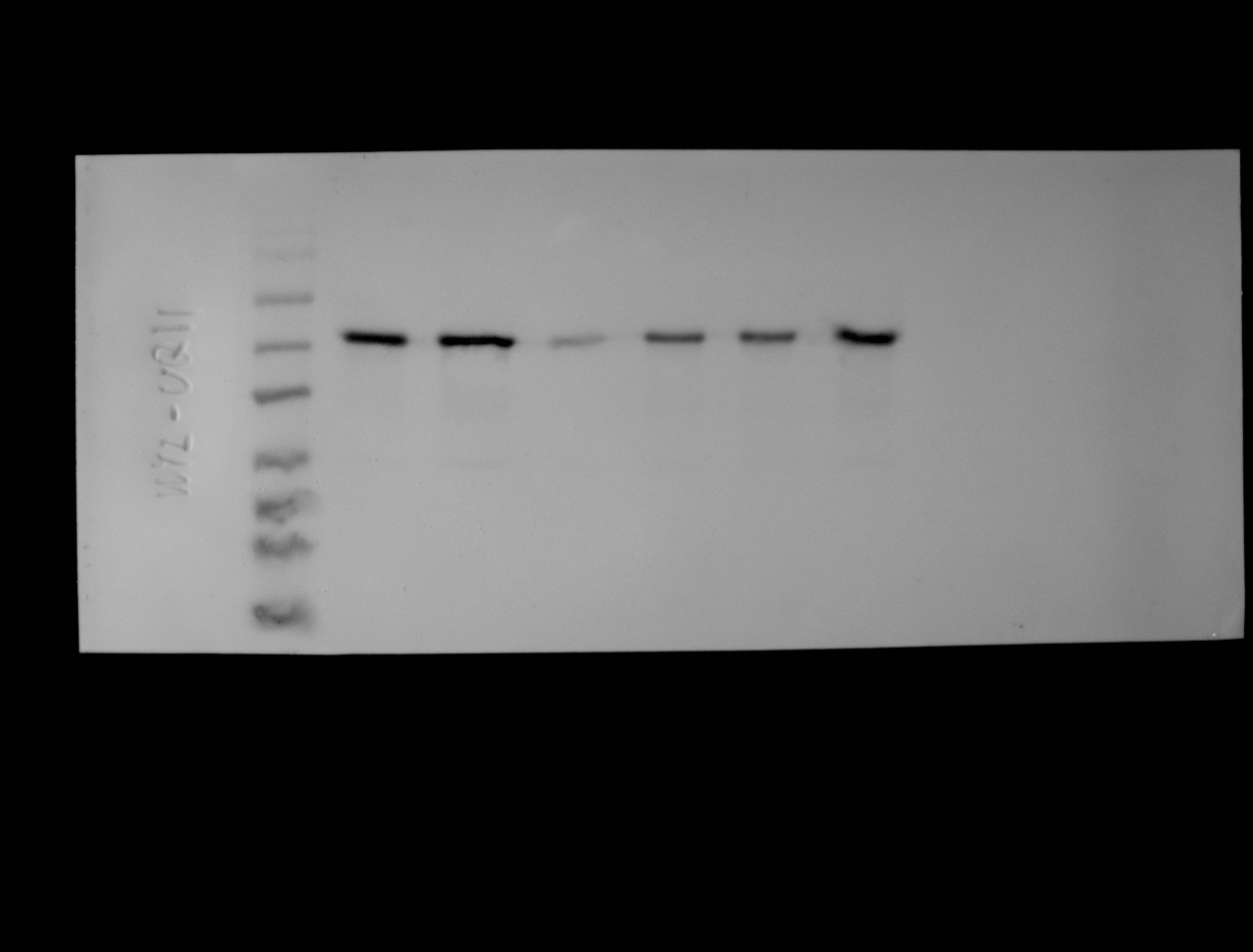
**

**
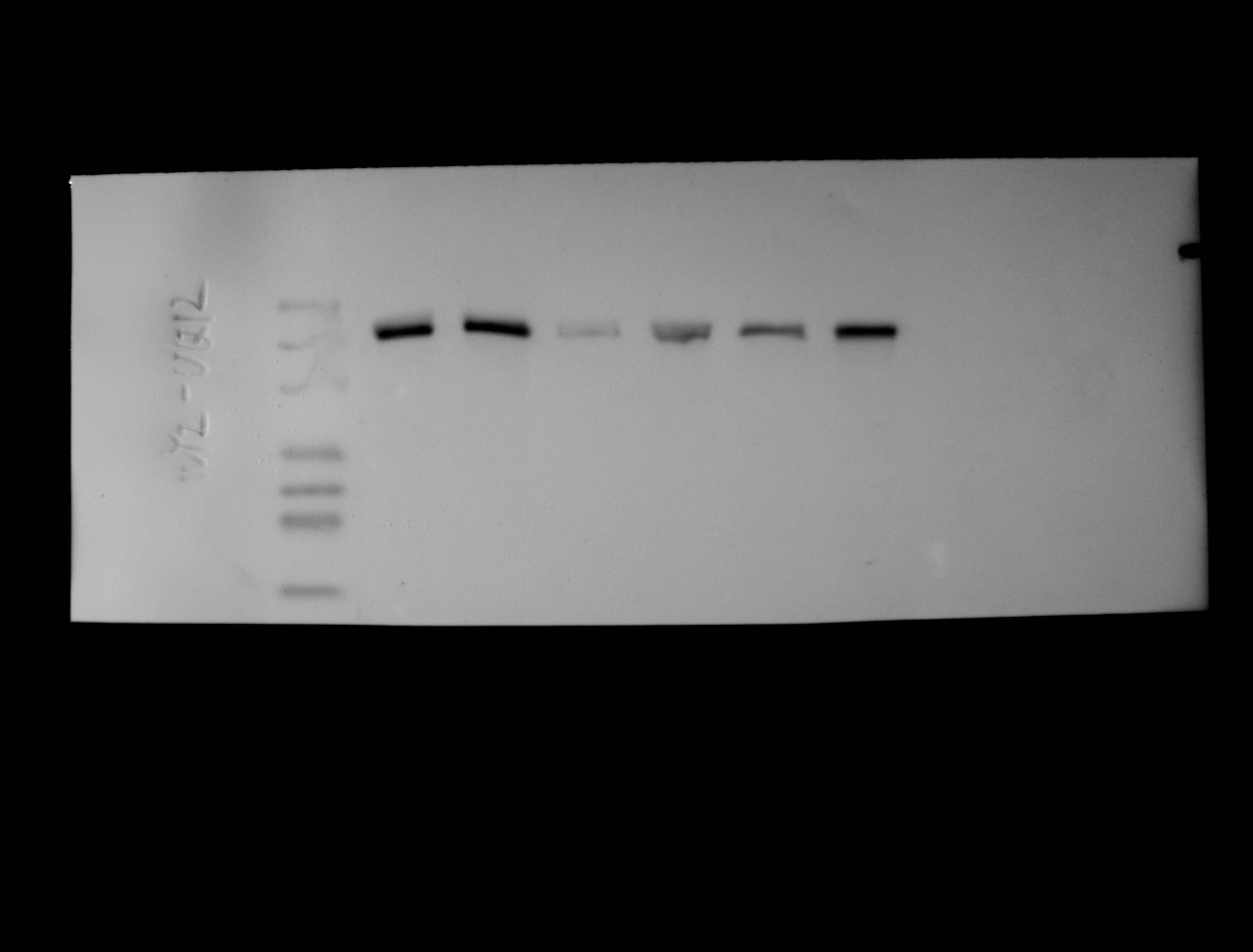
**

**
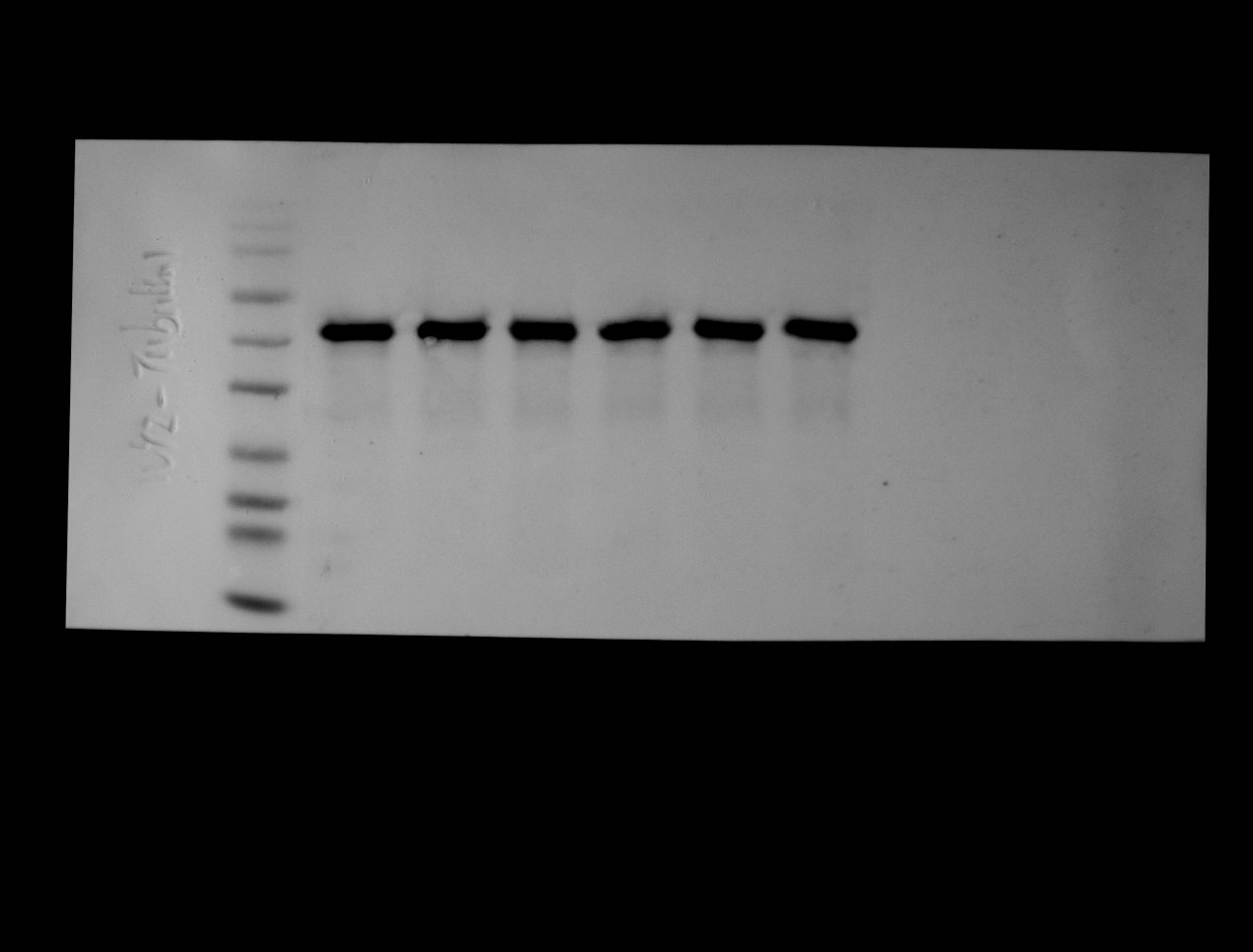
**

**
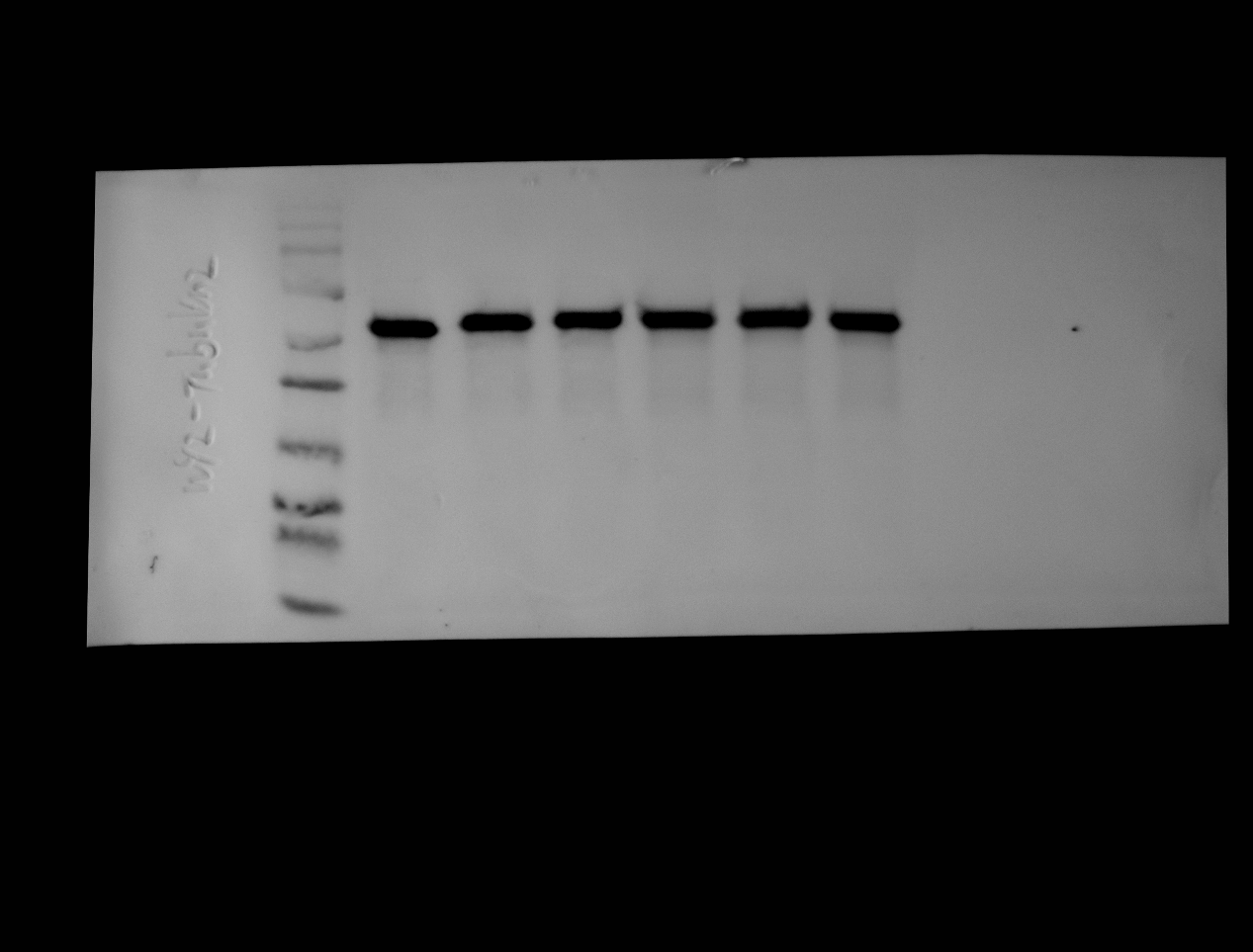
**

**
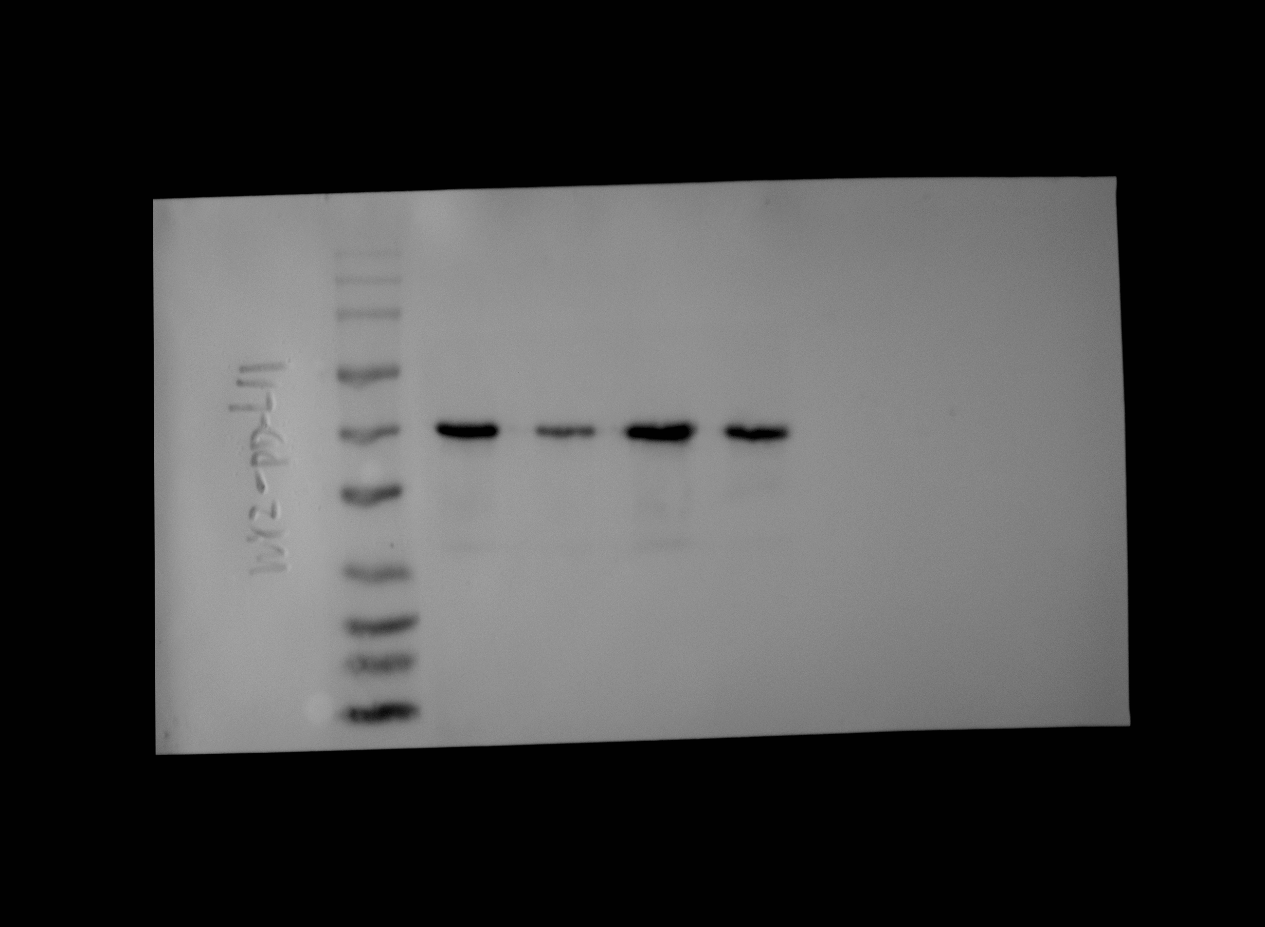
**

**
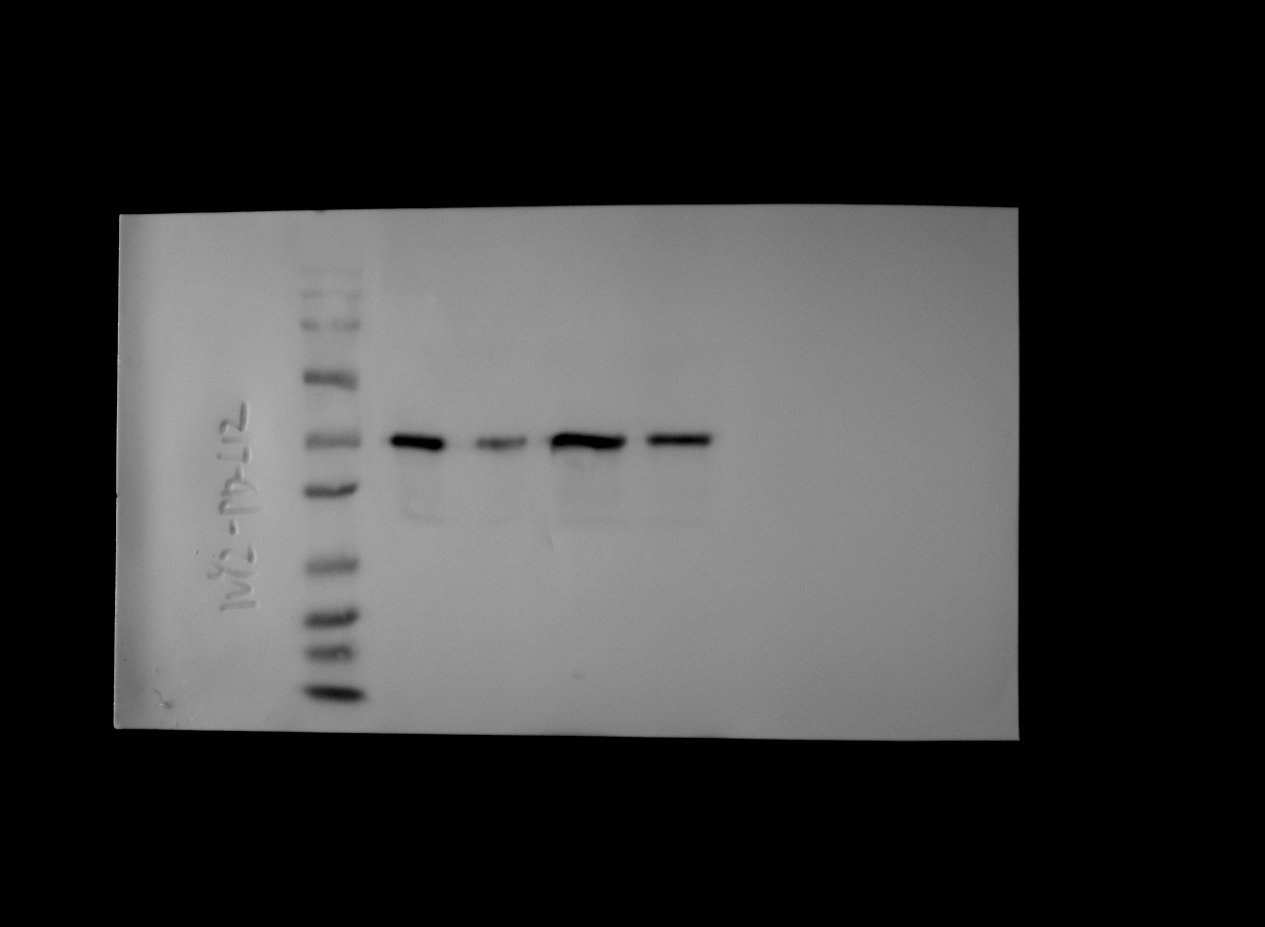
**

**
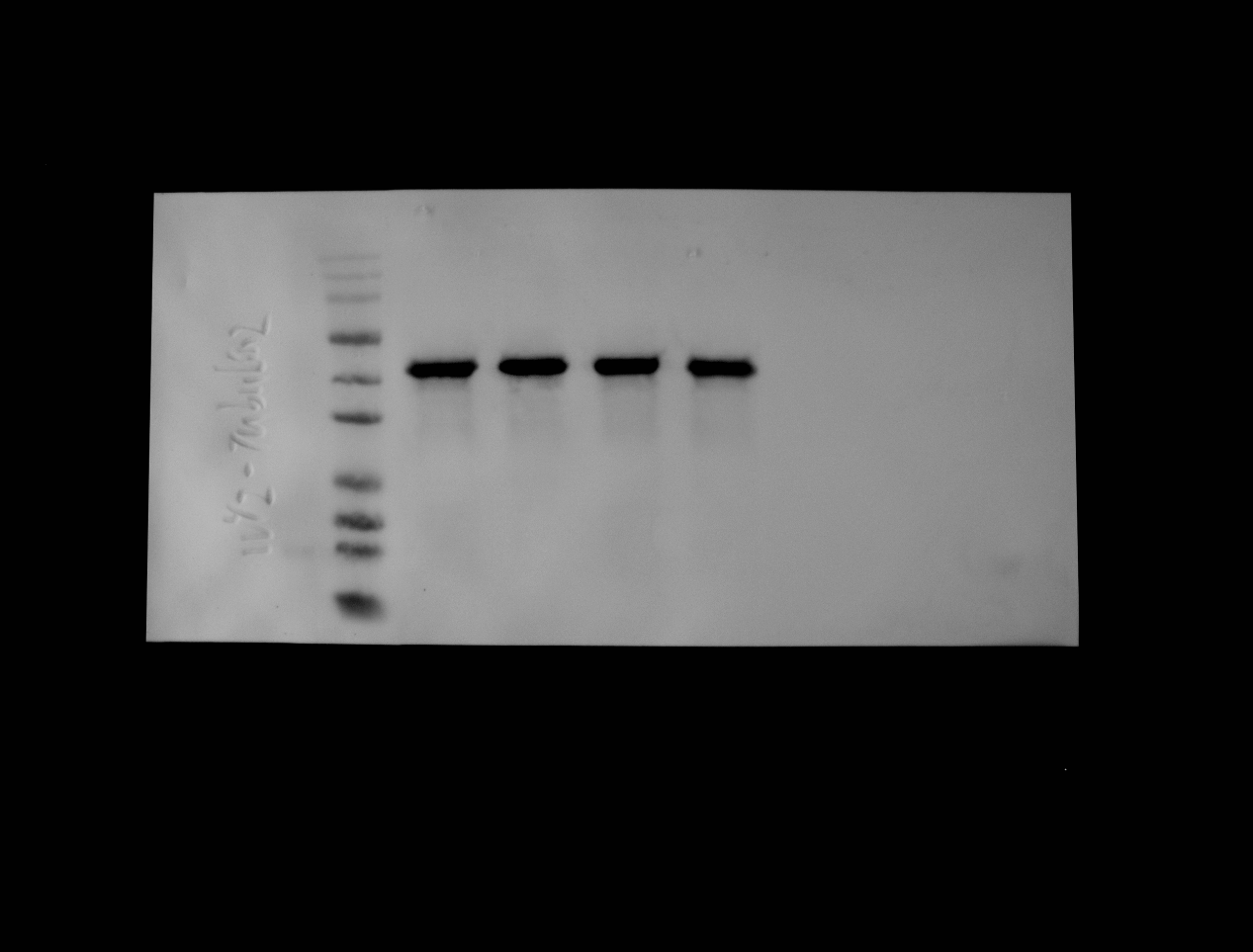

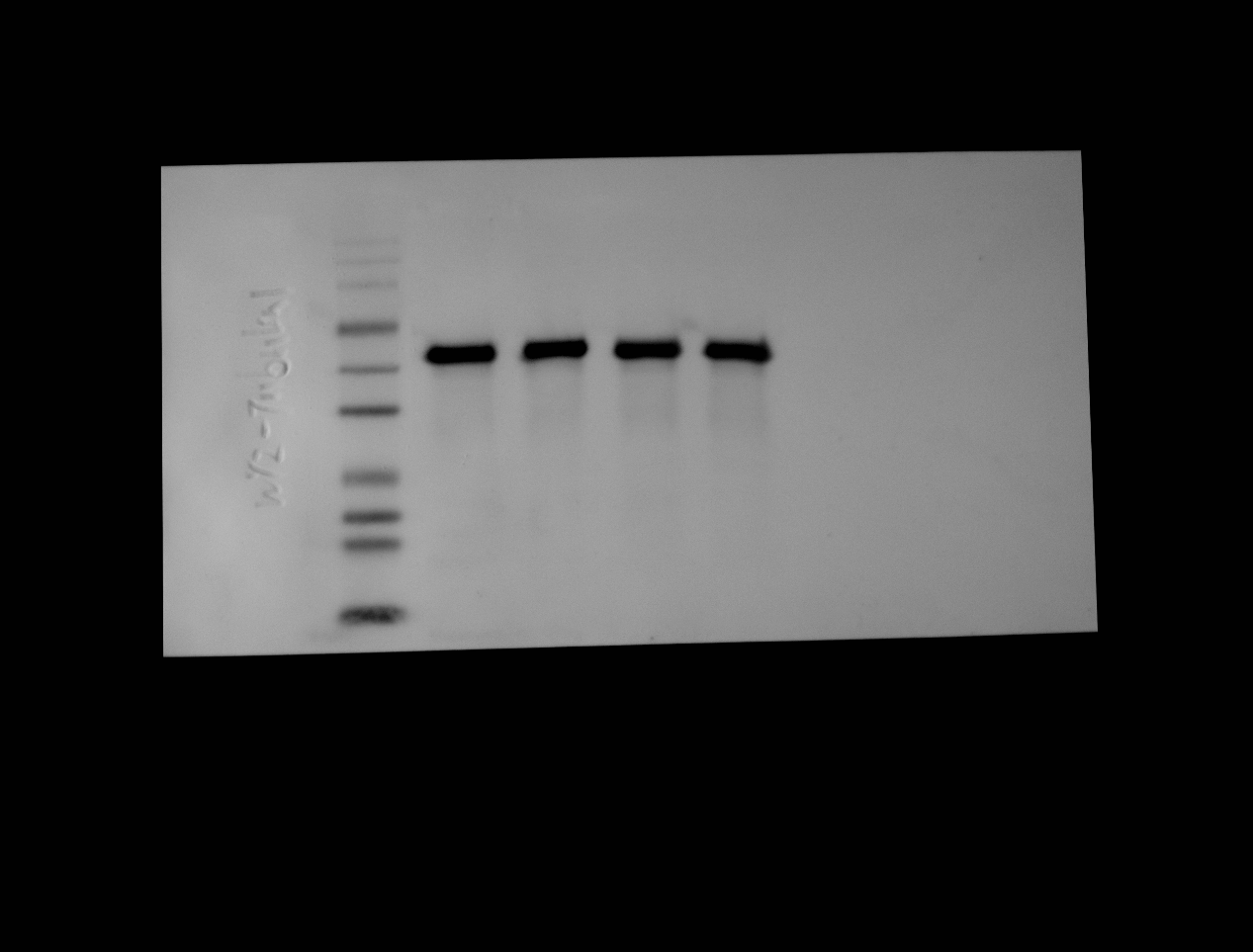
**


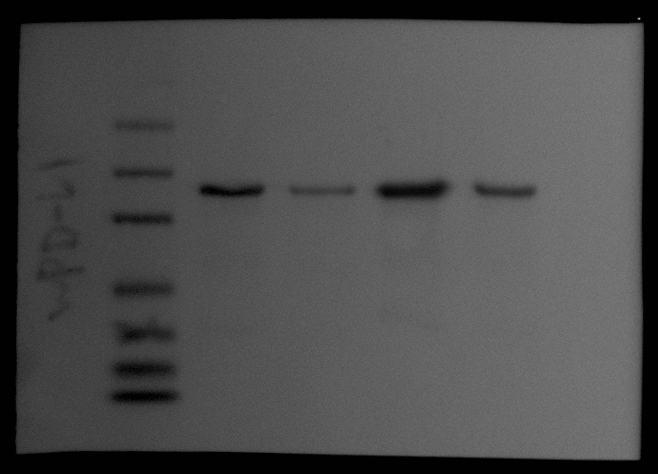


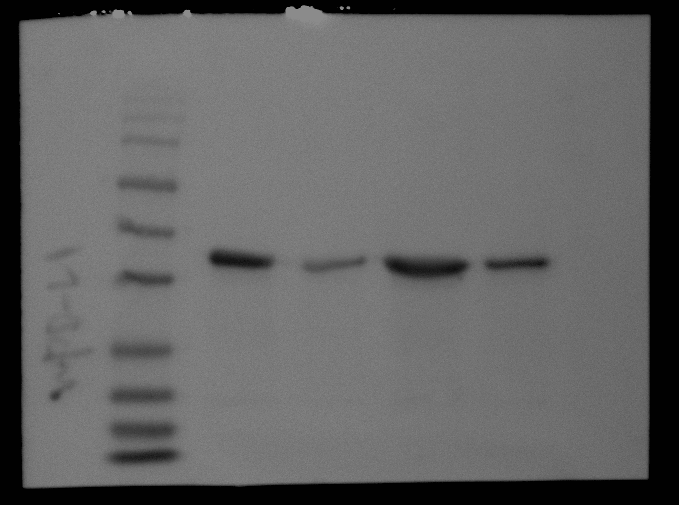


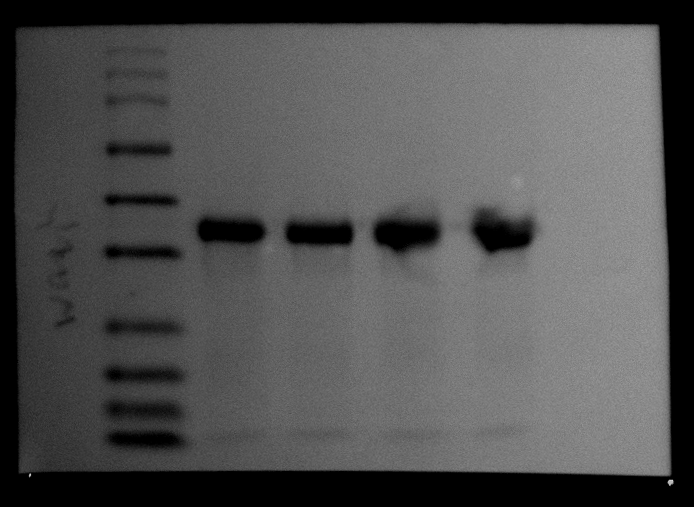


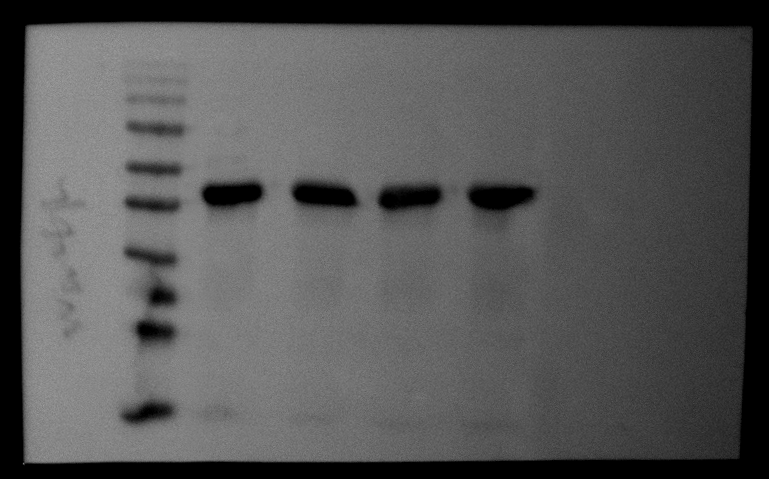

Supplement: Supplementary file 2 — Supplementary Material 2 [file 13046_2025_3477_MOESM2_ESM.docx]
